# Cascade Dynamics of Multiple Molecular Rotors in a MOF: Benchmark Mobility at a Few Kelvins and Dynamics Control by CO_2_

**DOI:** 10.1021/jacs.1c03801

**Published:** 2021-08-13

**Authors:** Jacopo Perego, Charl X. Bezuidenhout, Silvia Bracco, Giacomo Prando, Luciano Marchiò, Mattia Negroni, Pietro Carretta, Piero Sozzani, Angiolina Comotti

**Affiliations:** ⊥ Department of Materials Science, University of Milano − Bicocca, Via R. Cozzi 55, 20125 Milan, Italy; § Department of Physics, University of Pavia, Via Bassi 6, 27100 Pavia, Italy; † Dipartimento di Scienze Chimiche, della Vita e della Sostenibilità Ambientale, University of Parma, Parco Area delle Scienze 17/a, 43121 Parma, Italy

## Abstract

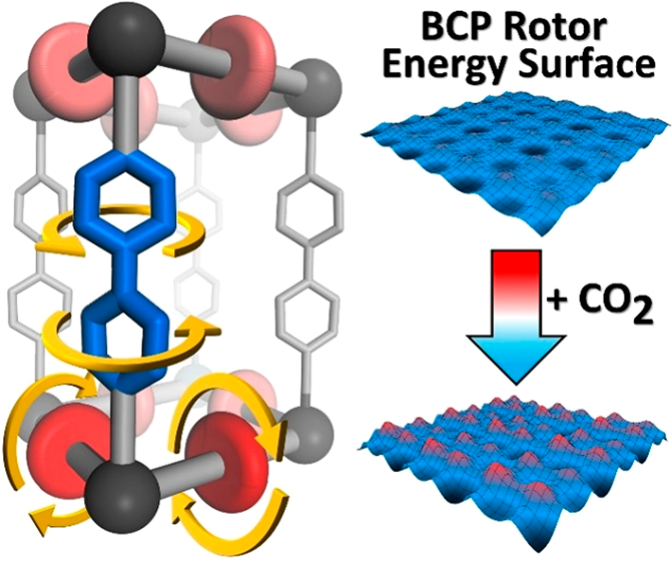

Achieving sophisticated juxtaposition of geared molecular rotors with negligible energy-requirements in solids enables fast yet controllable and correlated rotary motion to construct switches and motors. Our endeavor was to realize multiple rotors operating in a MOF architecture capable of supporting fast motional regimes, even at extremely cold temperatures. Two distinct ligands, 4,4′-bipyridine (bipy) and bicyclo[1.1.1]pentanedicarboxylate (BCP), coordinated to Zn clusters fabricated a pillar-and-layer 3D array of orthogonal rotors. Variable temperature XRD, ^2^H solid-echo, and ^1^H T_1_ relaxation NMR, collected down to a temperature of 2 K revealed the hyperfast mobility of BCP and an unprecedented cascade mechanism modulated by distinct energy barriers starting from values as low as 100 J mol^–1^ (24 cal mol^–1^), a real benchmark for complex arrays of rotors. These rotors explored multiple configurations of conrotary and disrotary relationships, switched on and off by thermal energy, a scenario supported by DFT modeling. Furthermore, the collective bipy-ring rotation was concerted with the framework, which underwent controllable swinging between two arrangements in a dynamical structure. A second way to manipulate rotors by external stimuli was the use of CO_2_, which diffused through the open pores, dramatically changing the global rotation mechanism. Collectively, the intriguing gymnastics of multiple rotors, devised cooperatively and integrated into the same framework, gave the opportunity to engineer hypermobile rotors (10^7^ Hz at 4 K) in machine-like double ligand MOF crystals.

## Introduction

The mechanics of motion in solids has been attracting increasing interest from the perspective of designing organized molecular rotors, motors, and machines, the goal being to control their functions and properties, such as the commutation of light into mechanical work, dielectric and optical properties, and ferroelectricity.^1−6^ Different strategies have been addressed for the targeted construction of dynamic materials, including the use of self-assembly principles, host–guest compounds, and hybrid materials.^7−15^

Molecular rotors, which have been successfully engineered to be assembled into dynamic structures, were designed by obeying to a few criteria.^12,16^ The flexibility about pivotal bonds as a consequence of a low rotational energy profile has been accomplished by the use of carbon–carbon covalent bonds with marked sigma character. Adjacent bonds and groups can influence rotational flexibility, for example, pivotal bonds connected to triple bonds can favor fast dynamics, while extended electronic conjugation between the rotors and the neighboring groups, as may happen in *p*-phenylene units and carboxylic groups, can be detrimental. Frustrated-symmetry match between the symmetry of molecular rotors and adjacent groups is extremely effective in increasing the number of minima along a complete turn, flattening the energy profile upon rotation. Adequate free volume for molecular group rotation is a key parameter that is successfully fulfilled by the exploitation of low density and porous materials.^16−22^ All these strategies enabled the exquisite engineering of barriers to group-revolution of bicyclopentane-based rotors in the solid-state, down to the energy limit of a few calories per moles and motional rates of 10^7^ Hz even at a few kelvins.^23,24^

Within the realm of porous materials, MOFs are outstanding for their synthetic versatility and the opportunity they provide to design modular structures, while preserving crystalline order and periodicity.^25,26^ Moreover, MOFs have been shown to support extensive dynamics without disrupting the primary architecture. For this reason, MOFs were successfully employed to insert rotors in the frameworks as ligands bridging the metal ions or cluster nodes.^27−30^ The project to assemble multiple fast rotors of increasing complexity within a single ordered framework is further challenging, since the framework not only preserves their peculiar mobility but enhances their synergistic potential for realizing a cooperative smart organization with emerging functions. Pillared-MOFs offer this opportunity being composed of two juxtaposed ligands, which can be designed as molecular rotors. Additionally, due to their structural flexibility and tunability, they did show superior properties and have been successfully proposed for reversible gas-capture and water sorption from the air.^31−35^

Herein, we engineered bicomponent MOFs built from two distinct ultrafast and interacting molecular rotors of diverse chemical nature and symmetry (saturated vs unsaturated moieties and 2-fold vs 3-fold symmetry). They are organized as 2D layers comprising bicyclopentane dicarboxylate (BCP) units and bipyridine pillars forming a multidynamical architecture, wherein the rotors experience sequential motional behavior activated at distinct temperatures. The dynamics of bipyridine rotors are coupled with framework dynamics through a hydrogen-bonded network, which switches at low temperature to the ordered state and consequently triggers the hyperfast motion of bicyclopentane dicarboxylate in the 2D sheets. BCP rotors are set at a proper distance to generate multiple configurations of geared and antigeared rotators in a cascade of dynamical processes. Trajectories and energy barriers were determined by ^2^H solid-echo NMR and ^1^H *T*_1_ relaxation times combined with DFT calculations. In the lowest energy configuration, BCP rotors explore an exceptionally low activation energy of 100 J mol^–1^ (24 cal mol^–1^) and a unique full-range of rotational frequencies (10^3^–10^8^ Hz), forbidden at such low temperatures to most molecular rotors. DFT calculated a flat-energy landscape, achieving the intensely pursued goal of collective unhindered continuous rotation in solid matter. The extreme mobility makes the porous crystal sensitive to external stimuli such as low-pressure CO_2_ gas, which can selectively modulate the multiple rotational phenomena at will. Surprisingly, variable pressure *in situ* PXRD detected the effect of gas molecules entering the porous crystals and CO_2_-induced framework ordering coordinated with rotor dynamics. The inclusion of CO_2_ greatly affected the mechanism, speed, and activation energy of the rotators.

## Results and Discussion

The pillared double-rotor MOF was synthesized by self-assembly of two distinct molecular rotors (BCP = bicyclo[1.1.1]pentane-1,3-dicarboxylate; bipy = 4,4′-bipyridine) and Zn ions under solvothermal conditions at 85 °C in DMF/MeOH mixture. The highly crystalline powder was filtered and washed with fresh solvent (SI). Activation under a high vacuum at 140 °C effectively removed guest molecules, generating permanent porosity and providing excess free volume that could sustain fast rotational motion and dynamics in the solid state (named FTR-P1, pillared free trigonal rotor). Infrared spectroscopy showed a shift of the C–O stretching band from 1664 to 1596 cm^–1^, which signifies the coordination of BCP carboxylate groups to Zn ions. ^13^C and ^1^H MAS NMR demonstrated the purity of the samples and complete guest removal. TGA showed the robustness of the compound up to 330 °C. The open porosity of samples was proven by CO_2_ adsorption isotherms at 195 K yielding a maximum adsorbed amount of 1.7 mmol/g (about 4 molecules per unit cell), which corresponded to the filling of the accessible free volume estimated by the crystal structure (Figure S15).

### Order and Disorder in the Crystal Structure

**Figure 1:**
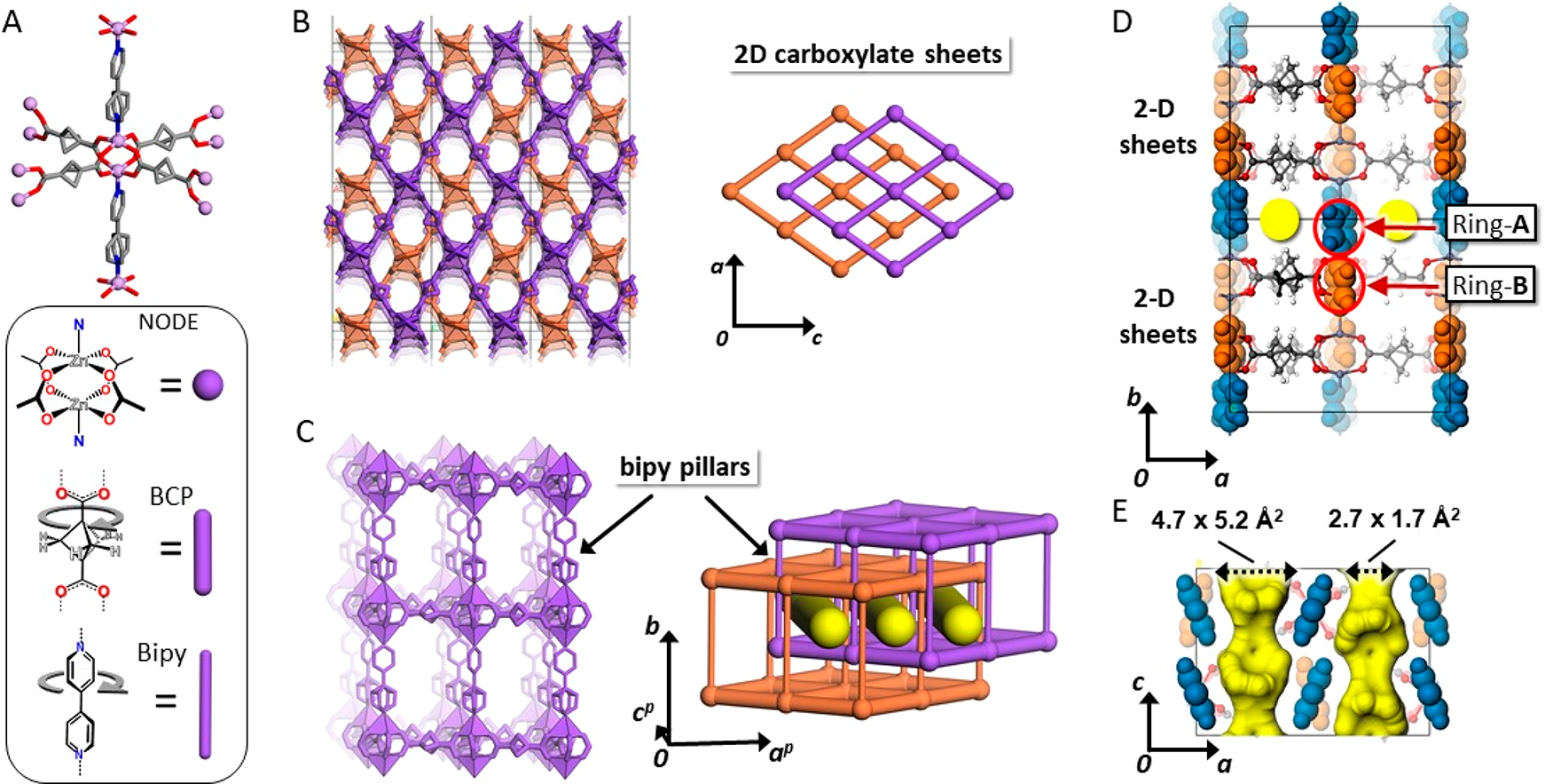
(A) Zn-paddlewheel node and molecular structures of BCP and bipy linkers. (B) (left) Crystal structure of FTR-P1 showing the two interpenetrated networks. The metal atoms are represented as solid tetrahedrons. (right) Depiction of the two independent networks, orange and violet. (C) (left) Connections between linkers and nodes within a single network (bipy and BCP in violet). (right) Two interpenetrated networks (highlighted in violet and orange colors) forming channel-like cavities. (D) Crystal structure highlighting ring A facing the channel and ring B in the 2D layer highlighted in blue and orange, respectively. (E) Open channels and ring A highlighted in yellow and blue, respectively.

The activated material was subject to variable temperature XRD, as both single crystals and powders. Single-crystal XRD data collection was performed between 275 and 110 K. The crystal structure is formed by two independent and interpenetrated 3D networks. The 3D networks consist of 2D sheets comprising BCP carboxylate ligands coordinated to the Zn cations in a paddle wheel fashion, forming a rhombic geometry (Figure 1), which are pillared by bipy ligands, generating the 3D framework with **pcu** topology.^36^ The interpenetrated crystal structure exhibits 1D open channels running parallel to the *c* axis, which are decorated by the bipyridine aromatic units. The channel cross-section ranges from 4.7 × 5.2 Å^2^ to 2.7 × 1.7 Å^2^, confirming the generation of an ultra-microporous material. The aromatic rings of bipy units can be differentiated into distinct moieties; one is exposed to the empty cavities (ring A), while the other is situated within the rhombus of the 2D layers (ring B) (Figure 1D).

**Figure 2:**
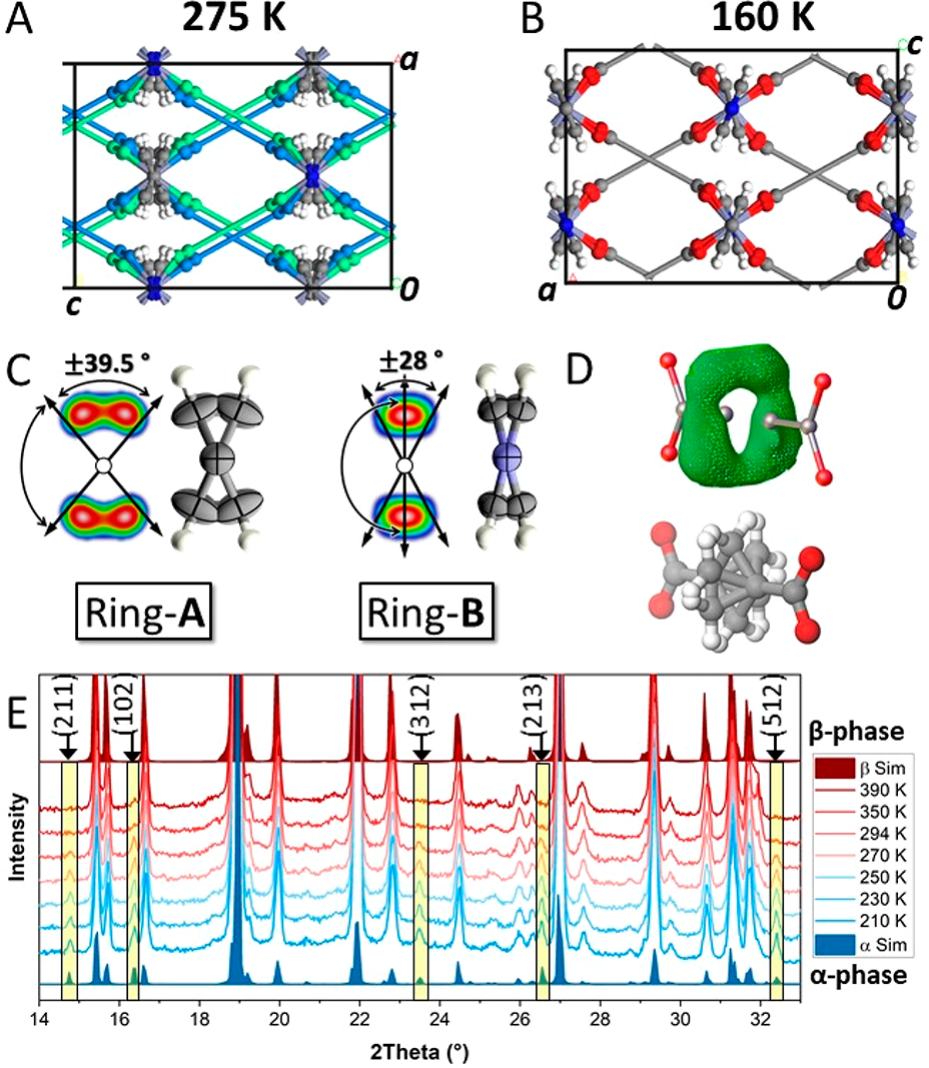
Crystal structures of FTR-P1 with (A) symmetry related BCP dicarboxylated axis (β-phase, blue and green rods) and (B) ordered BCP axis (α-phase, gray rods). (C) Diffused electron density and thermal ellipsoids with 80% probability of bipy rings as viewed along the main rotation axis. (D) Diffused electron density with a toroidal shape about the main BCP axis, showing the dynamics of the BCP rotor. (E) VT-PXRD patterns as a function of temperature showing the transition from the low temperature to high temperature phase. New distinct PXRD peaks appear in the α-phase as indicated by the Miller indices. For comparison, the simulated profiles derived from SCXRD resolution of β- and α-phases are displayed.

The crystal structure at high temperature exhibited extensive disorder on both bipy and BCP ligands, suggesting the presence of distinct rotors. The crystal structure (space group *Cmma*) shows 2D disordered sheets due to two symmetry-related locations of the main BCP-axis (50% occupancy) shifted parallel to one another (Figure 2A). This disorder can be generated by a rotation of the paddle-wheel node around its axial coordination axis. In fact, the bipyridine ring exposed to empty cavities (ring A) displays orientational disorder of its aromatic rings over 2-sites (±39.5°) with a symmetry site-occupancy of 50% and forms C–H···O interactions with the disordered carboxylate oxygen atoms of the other network (Figure S18, D···A distance = 3.42 Å, D–H···A angle of 170.4°). The rotation of the paddle-wheel weakens the C–H···O interactions and prompts the rotation of ring A and or vice versa, generating a “gymnastics” between the bipy ring A and the metal-carboxylate 2D layer (Figure 2A,C). Meanwhile the bipy ring B, which lies among the BCP units and within the 2D layer, experiences wide oscillations up to ±28° (Figure 2C). At 160 K, an ordered phase of lower symmetry (space group *Pcca*) is formed corresponding to either of the disordered networks present in the high-temperature structure (Figure 2B).

Furthermore, a diffused electron density describing a toroidal shape about the main molecular axis of BCP and corresponding to CH_2_ moieties is observed: a preliminary indication for the presence of flat energy profiles explored by the methylene crown (Figure 2D). Consequently, the disorder in both ligands suggests the presence of two types of rotators (bipy and BCP) in the crystalline porous architecture.

The phase transformation from the low temperature phase (denoted α-phase) to the high temperature phase (β-phase) could be followed accurately by variable temperature powder X-ray diffraction patterns (VT-PXRD). Upon heating, diffractograms collected between 210 and 390 K show the progressive disappearance of the peaks diagnostic of the low-symmetry phase (Figure 2E). In fact, the marked difference between the two phases is due to the systematic extinctions associated with the cell centering (extinction *hkl* with *h* + *k* = 2*n* + 1 in *Cmma*, but not extinct in *Pcca*), while the remaining peaks are virtually unaltered because the unit cell parameters do not change. The peaks of systematic extinctions are identified in the experimental pattern and occur without overlapping with the remaining Bragg reflections. The profiles were processed by two-phase Rietveld refinement and interpreted as the contribution of two independent structures with distinct relative ratios (Figure S25 and Table S2; the errors in the ratio of phase determination range from 1.8% to 3.1%).

Consistently, DSC traces, described later on, indicate a phase transition that spans over a wide range of temperature wherein the low temperature phase coexists with that at high temperature in the intermediate temperature range. This transition involves the second-coordination bonds and a moderate enthalpy.

### Bipy Rotor Dynamics

**Figure 3:**
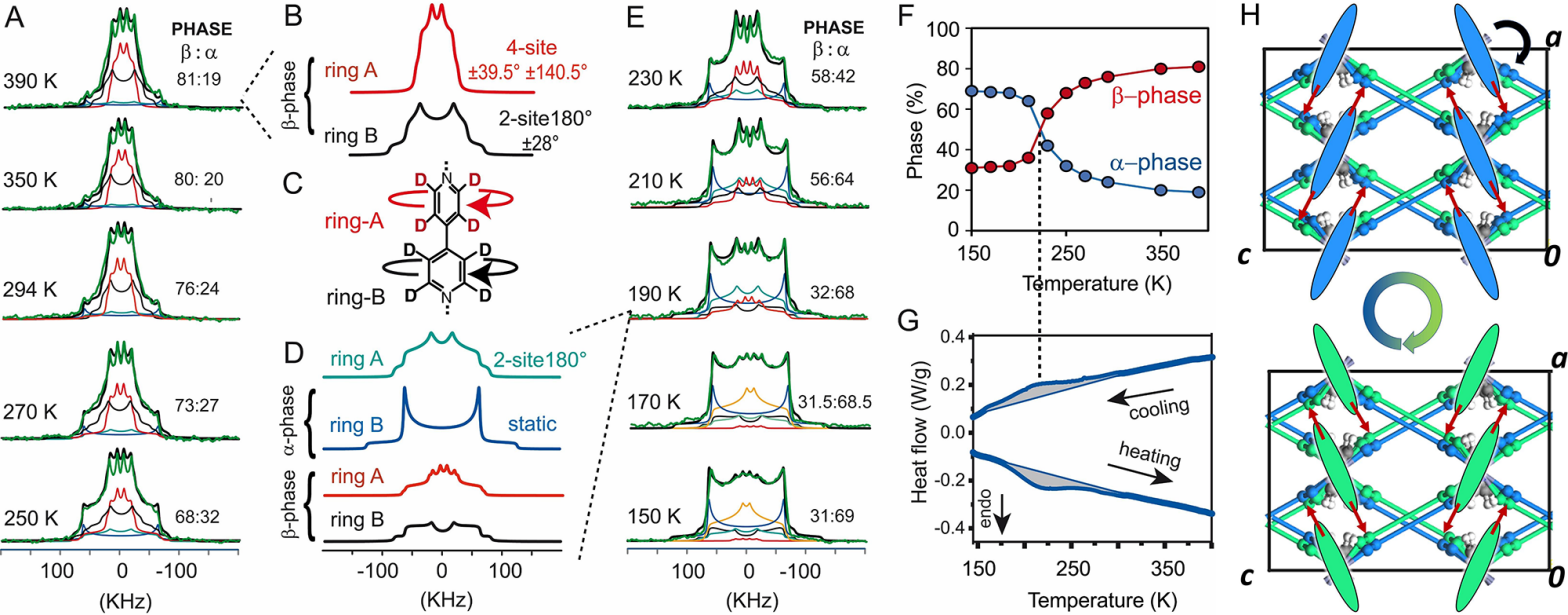
^2^H NMR spectra of FTR-P1d (A) from 250 to 390 K and (E) from 150 to 230 K. Deconvoluted components of ^2^H NMR spectra at 390 K (B) and 190 K (D). (C) Molecular structure of perdeuterated-bipy showing the aromatic rings A and B. (F) Quantification of α- and β-phase from the deconvoluted ^2^H NMR spectra. (G) DSC traces exhibiting the transition from the ordered to the disordered phase. (H) Bipy ring A arrays viewed along the main rotational axis (blue and green pales) alternatively coordinated with the blue and green frame.

Solid state NMR spectroscopy gives insight into the dynamics of materials: in particular, spin–lattice relaxation times (*T*_1_) and ^2^H NMR are among the most informative methods for discovering molecular rotors and their motional properties such as the rotational frequencies, associated energies, and mechanism of motion (SI).^37^ To infer these properties, we synthesized an analogous MOF with BCP and perdeuterated bipyridine (FTR-P1d) to selectively study the dynamics of bypiridine molecules by ^2^H spin–echo NMR. FTR-P1d exhibits virtually the same properties, as indicated by PXRD trace and CO_2_ isotherm (Figures S16 and S22). Variable temperature ^2^H solid-echo NMR spectra were interpreted as the summation of contributions of the two distinct phases and the two independent A and B rings (Figure 3A–E and SI).^38,39^ The β-form is the main contributor in the high temperature spectra (293–390 K): the rings facing the channels (ring A) experience fast reorentational jumps (about 5 × 10^6^ Hz) among 4 conformational minima with a 4-site jump mechanism (reorientational angles of +39.5°, +140.5°, −140.5°, and −39.5°) (Figure 3A). These ring jump-angles match the SC-XRD observations (Figure 2C). The 4-site jump mechanism comprises breaking and reforming of the C–H···O interactions coupled with the rotational dynamics of the paddle-wheel. The ring B, which resides within the 2D layer, essentially underwent a fast 180° flip reorientation coupled with ±28° jumps, accounting for the shrinking of the profile at high temperatures. The ±28° jumps are in agreement with the diffused electron density from X-ray diffraction data (Figure 2C).

In the spectra below 210 K (Figure 3E), the more ordered α-phase prevails, as highlighted by the emergence of two singularities separated by 124.7 kHz width due to a fully static arrangement of confined ring B within the NMR time scale (<10^3^ Hz). However, the ring A, which is exposed to the channels, experiences only a fast 2-site 180° flip reorientation and no 4-site reorentations. The 4-site reorientation mechanism can only be supported by rotational dynamics of the paddle-wheels, which become ordered at temperatures below 210 K (Figure 2B).

These sophisticated dynamics are strongly modulated by temperature, changing both the α- vs β-phase content ratio (Figure 3F) and reorentation frequencies of each phase (Figure 3A,E). In particular, upon lowering the temperature, the ring A in the β-phase shows restricted motion, progressively reducing the 4-site mechanism and favoring simple oscillations of ±39.5° but still maintaining fast frequencies of 10^8^ Hz. Conversely, in the α-phase, ring A maintains its 180° flip reorientation with a frequency of 4 × 10^6^ Hz, even at temperature as low as 150 K. Interestingly, in the α-phase, the differentiation between the two bipy rings reaches an extreme: highly mobile rotor (ring A) and a static ring (ring B) are observed, while both rotors are mobile in the β-phase.

From the deconvoluted profiles, the α- and β-phase ratios are depicted in the diagram of Figure 3F: the maximum rate of change for the transition is observed at 220 K. This transition from the ordered to the disordered phase with increasing temperature mirrors the DSC trace, which shows an endotherm with a maximum at 220 K (Δ*H* = 21–23 J/g) (Figure 3G).

Low rotational energy barriers of 1.2 and 2.0 kcal/mol were estimated for rotor A, exposed to the channels, in the β- and α-phases, respectively. Rotator B exhibits a higher barrier of 3.9 kcal/mol in the β-phase and a “static” arrangement (*k* < 10^3^ Hz) in the α-phase (Figure S31). The energy barriers are lower than those reported in the literature for bipy in solid-solution MOFs.^29^

By applying the Eyring equation, we can extract the entropy change from the ground to the excited state. Rotator A in the disordered β-phase shows a negative value of −26 cal mol^–1^ K^–1^ and a rotational frequency at infinite temperature *K*_0_ = 3 × 10^7^ Hz, which is much lower than that expected from the inertial mass of a single rotator (2 × 10^12^ Hz);^40^ therefore, cooperative reorientation must be invoked. Essentially, the pillaring bipy rotors operate collectively in a concerted manner with neighboring rotors and the framework. These dynamics facilitate the breaking and re-formation of the C–H···O interactions between the two networks as ring A reorientates (Figure 3H).

### BCP Rotor Dynamics down to 2 K

**Figure 4:**
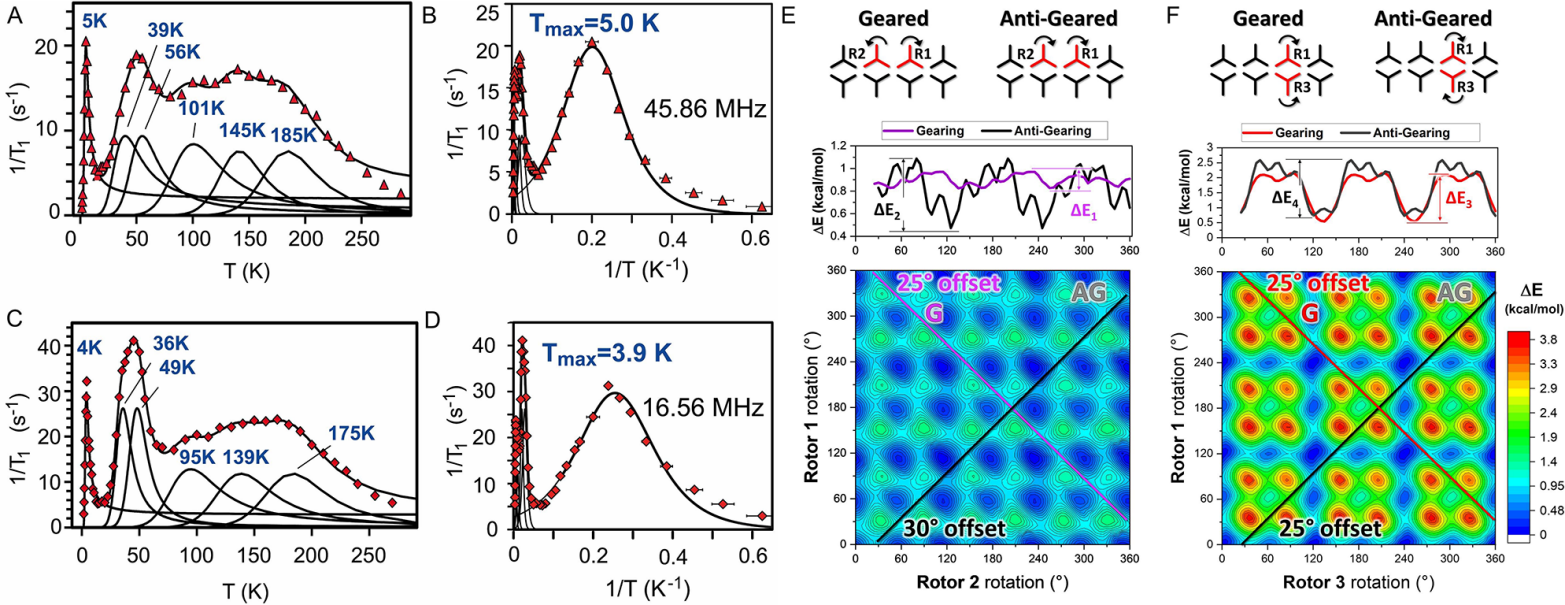
^1^H relaxation rates as a function of temperature (A, C) and reciprocal of temperature (B, D) at two distinct magnetic fields. The error bars on 1/*T*_1_ are smaller than the symbols. (E, F) 2D DFT scans for two side-by-side rotors (E) and two crossed rotors (F): (top) graphical illustration of the model indicating the geared and antigeared rotations with the curved arrows; (middle) Δ*E* plot for the most optimal gearing and antigearing rotations; (bottom) 2D contour map with the rotation of rotor pairs R1 and R2 (E) and R1 and R3 (F) on the *X*- and *Y*-axes with the colors indicating the Δ*E*. The color scale ranges from 0 kcal mol^–1^ (blue) to 3.8 kcal mol^–1^ (red). The lines indicate the most optimal gearing (G) and antigearing (AG) pathways on the energy surfaces, with the relative rotor rotational offset indicated for each.

Owing to the aforementioned diffused electron density of the BCP CH_2_ groups, the presence of fast molecular rotatory dynamics are expected (Figure 4). Variable temperature ^1^H *T*_1_ relaxation times of BCP hydrogens were extremely informative regarding the exceptional mobility of BCP rotors, which could not even be quenched at a low temperature of 2 K. The ^1^H *T*_1_ measurements of BCP hydrogens were carried out using a MOF sample containing deuterated bipy ligands (FTR-P1d), which eliminates the contribution of the bipy moieties to the ^1^H spectrum generating a single narrow resonance of BCP methylenes. Thus, ^1^H *T*_1_ relaxation times would not be affected by signal overlapping and spin-diffusion due to bipy.

The measurements, collected at distinct observation frequencies of 16.56 and 45.86 MHz, are reported in Figure 4A–D. The relaxation rates plotted as a function of temperature exhibit a complex profile, which can be fitted using six Kubo–Tomita (K-T) functions with maxima at 4, 36, 49, 95, 139, and 175 K. Surprisingly, at very low temperatures from 10 to 1.6 K, it is possible to identify a full K-T profile with a maximum relaxation rate at 4 K, demonstrating extremely high mobility of the BCP molecules in the 10^7^ Hz regime (Figure 4D, expansion at low *T*). Indeed, the activation energy for this process is as low as 100 J mol^–1^ (24 cal mol^–1^). The rotor shows a 6 orders-of-magnitude change in frequency, from 10^3^ Hz to 10^8^ Hz in the small temperature range of 1–7 K, implying that the rotator exhibits a pronounced tendency to thermally activated motion. Such dynamical behavior at low temperatures is only comparable to that of methyl rotation and BCP rotors in Zn-FTR (*E*_a_ = 25.9 J mol^–1^, 6.2 cal mol^–1^).^23,41−43^ The activation energy is much lower than the BCP rotor pivoted in between two ethynyl groups (*E*_a_ = 6.813 J mol^–1^, 1630 cal mol^–1^)^44^ and BCO in Zn-MOF (*E*_a_ = 770 J mol^–1^; 185 cal mol^–1^).^28^ Above 10 K, the motional process explores a succession of phenomena with gradually increased activation energies of 445, 688, 940, 1816, and 2947 cal mol^–1^ and correlation times (τ_0_) of about 10^–10^ s.

These phenomena were interpreted as various configurations of groups of neighboring rotators with relative rotational directions, co-rotating or counter-rotating. Since individual BCP rotators cannot be differentiated in the unit cell, we resort to accounting for intermolecular interactions among rotators during their rotation. We built a model considering an ensemble of eight vicinal rotators as arranged within the crystal structure (Figure 4E,F).

### DFT Modeling

DFT calculations of the energy profiles were determined for rotor-couples (side-by-side rotators R1–R2 and crossed rotators R1–R3) employing a 2-D potential energy scan of both rotors in the pair (Figures S36 and S37) and an ensemble of eight vicinal rotators as arranged within the crystal structure. Each rotor was scanned about its rotation axis, and the interaction with surrounding BCP units was taken into account. The energy barrier for single rotor rotations was identified to be 0.9–1.0 kcal mol^–1^ and therefore cannot generate lower barriers. The potential energy contour map for a couple of side-by-side rotors, R1 and R2, allowed us to identify the preferential paths comprising the reciprocal motion of the two rotors, yielding the lowest activation barriers (Figure 4E). The purple line with 45° negative inclination indicates the geared synchronous motion of the two rotors (R1 rotating clockwise and R2 rotating counterclockwise). A geared motion with an offset of 25° between the two rotators exemplifies the lowest energy pathway with a 70 cal mol^–1^ energy barrier. Various mechanisms of motion with higher barriers can be explored following different paths on the energy surface, such as the antigeared mechanism, indicated by the black line (45° positive inclination), with an energy barrier of 310 cal mol^–1^ and an offset of 30°.

The simultaneous rotation of the crossed rotators, R1 and R3 (Figure 4F), produces higher barriers of 750 cal mol^–1^ and 925 cal mol^–1^ for the geared and antigeared mechanisms, respectively. The rotors have more kinetic energy at higher temperatures to explore the rotational energy landscape, thus exploring several other paths that entail higher rotational energy barriers (Figure S38). Consequently, the calculated geared and antigeared rotational barriers for rotors in different configurations perfectly explain the series of experimental energy barriers measured from ^1^H *T*_1_ relaxation times. Remarkably, the lowest energy barrier at very low temperature is generated by the synchronized geared motion of side-by-side rotators.

### Rotor Modulation by CO_2_

**Figure 5:**
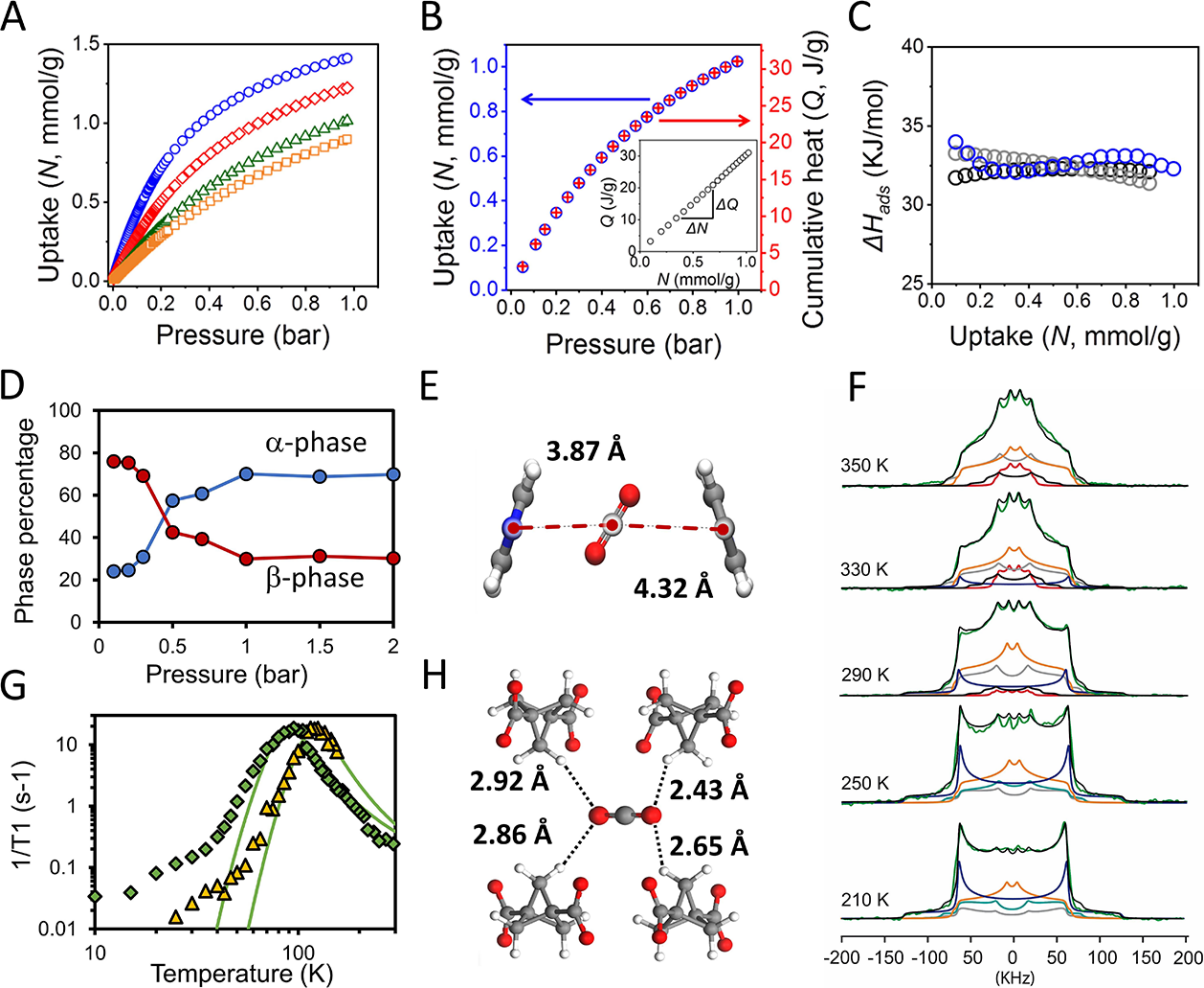
(A) CO_2_ adsorption isotherms at 273, 283, 293, and 298 K (blue circles, red diamonds, green triangles, and yellow squares, respectively). (B) CO_2_ loading (*N*) at 293 K and cumulative heat (*Q*) released on increasing loading with increasing the pressure. The inset shows a plot *Q* vs *N* with the gradient indicated. (C) Isosteric heat of adsorption calculated from isotherms fitted with Langmuir equation (gray circles) and Langmuir–Freundlich equation (light blue circles) compared to heat released from direct measurement at 293 K and increasing pressure (blue circles). (D) Phase percentage at 293 K and on increasing loading up to 2 bar. (E) Crystal structure of FTR-P1d loaded with CO_2_ at 253 K and 1 bar: an individual CO_2_ molecule sitting in the pocket and interacting with bipy molecules. (F) ^2^H NMR spectra of FTR-P1d loaded with CO_2_ (3.5 bar) as a function of temperature (black line). The total simulated profile is highlighted in green. The deconvoluted line shapes for each rotational mechanism are the following: 4-site ±39.5°/±140.5° reorientation for ring A in the β-phase (red line), 2 site 180° and 180°/±28° reorientation for ring B in the β-phase (gray and black lines, respectively), 2 site 180° reorientation for ring A in the α-phase (light blue line), static pattern for ring B in the α-phase (blue line), and 180° ± 39.5° jumps for ring A in both phases (yellow line). (G) ^1^H T_1_ relaxation times of BCP at 45.86 MHz of FTR-P1d loaded with CO_2_ (3.5 bar). The data were fitted by two K-T equations, giving rise to energy barriers of 1.2 and 1.8 kcal/mol (green diamonds and yellow triangles, respectively). (H) Single CO_2_ molecule confined in the pocket, highlighting the interaction with BCP moieties.

We explored the CO_2_ adsorption capability of the MOF at variable temperature (273–298 K) and up to 1 bar: FTR-P1d reaches 83% of the full loading at 273 K (Figure 5). The isotherms follow a Langmuir profile and yield an isosteric heat of adsorption of 32.5 kJ mol^–1^ according to the van ‘t Hoff equation. Independently, we performed a volumetric sorption experiment with direct *in situ* microcalorimetric measurements of the heat released during CO_2_ absorption (for the methodology refer to SI).^45,46^ The experiment was performed at 293 K up to 1 bar, obtaining a heat of adsorption of 32.4 kJ mol^–1^, which validates the isotherm derived from the isosteric heat of adsorption. Notably, the CO_2_ isotherm profile perfectly matches the cumulative heat profile for the adsorption process (Figure 5B), that is, the cumulative heat of adsorption (*Q*) increases linearly with CO_2_ loading (*N*). This suggests that the enthalpy of adsorption would remain virtually constant over a wide pressure range and is ascribed to the microporous nature of the MOF. In fact, the constricted 1D channels are progressively occupied site-after-site and CO_2_ molecules in each site display interactions exclusively with pore walls at the expense of CO_2_–CO_2_ interactions. The matrix–CO_2_ interaction energy, as evaluated by PW-DFT calculations, is *E*_int_ = 32.7 kJ/mol, consistent with the above-reported experimental values.

To further study the MOF–CO_2_ structural relationship, variable pressure PXRD (VP-PXRD, at 293 K) and variable temperature PXRD (VT-PXRD, under 1 bar CO_2_) experiments were conducted. Both experiments unveiled a framework structural change, from the β-phase to the α′-phase (FTR-P1d·*x*CO_2_), upon sufficient accumulation of CO_2_ into the channels. A pressure-induced structural change to the α′-phase occurs under mild conditions with an onset pressure of 0.4 bar CO_2_ at RT (Figure 5D). The CO_2_ loaded crystal structure, produced by Rietveld refinement of the PXRD pattern at 253 K, established that each CO_2_ molecule in the channel is surrounded by two bipy rings (ring A) and four BCP ligands. The CO_2_ molecules are disordered over two equivalent crystallographic positions, tilted by an angle of about 34° with respect to the channel-axis and arranged parallel to the bipy rings at a short Ring_centroid_^···^C_CO_2__ distance of 3.87 Å (Figure 5E). Upon desorbing CO_2_, the crystal structure reverts back to the original disordered β-form.

In this scenario, we further highlight, by frequency-sensitive methods, how CO_2_ interactions with both rotors affect their dynamics in the 10^3^–10^9^ Hz frequency regime. ^2^H solid echo NMR spectra of the FTR-P1d sample under 3 bar of CO_2_ as a function of temperature (Figure 5F) showed a drastic reduction in rotational frequencies of bipy rings compared to those occurring at the same temperature in the empty FTR-P1d. Additionally, the mechanism can be modulated by CO_2_ inclusion: at room temperature the full-turn is suppressed for ring A (facing the channels) and only jumps of ±39.5° are preserved over the whole temperature range, demonstrating its restricted dynamics. Only at high temperatures 4-site reorientation and 2-site 180° flips are present, but the activation energy is increased to 5.2–5.3 kcal mol^–1^, owing to the protrusion of the rings temporarily into the channels, now occupied by CO_2_ molecules. This represents a penalty paid by the rotators A of 3.5 kcal mol^–1^ with respect to the empty sample. The effectiveness of CO_2_, loaded from gas phase, in hampering the mobility of bipy rings is confirmed by the preparation of an adduct obtained by crystallization with DMF, which showed a further increase of α′-phase as detected by ^2^H NMR (Figure S68).

A dramatic change in the ^1^H *T*_1_ measurements for BCP rotors occurs, showing suppression of multiple phenomena observed in the empty sample. The dynamic behavior is simplified, and the relaxation pattern can be fitted by two K-T equations (peak temperatures at 95 and 125 K). Two motional phenomena with derived energy barriers of 1.2 and 1.8 kcal/mol and correlation times (τ_0_) of about 1.8 × 10^–11^ s and 8.8 × 10^–12^ s, respectively, are recognized (Figure 5G). The interaction of CO_2_ with the BCP rotors dominates their rotational energy landscape since CO_2_ forms short C–H···O contacts with all the rotors, as highlighted in the crystal structure of the loaded sample (Figure 5H). Molecular mechanics calculations, using CO_2_ loaded FTR-P1 under periodic boundary conditions, confirm the increase in activation energy for all rotational phenomena previously observed at lower activation energies in the empty compound (SI). These results clearly demonstrate the ease of manipulating the dynamics of two distinct rotors by an external stimulus due to the porosity of the MOF, which enables diffused-in molecules to act directly on the rotators.

## Conclusions

Two arrays of fast rotors were engineered in the crystalline structures of pillared MOFs, FTR-P1 and its deuterated analogue FTR-P1d. The assembly of fast molecular rotors generates a sophisticated dynamical scenario due to the interplay of distinct mechanical behavior and diverse motional regimes. The rotors operate in spatiotemporal succession, covering a temperature range from 390 K down to 2 K, with a multiple *pyrotechnic* motional evolution, while exploring systematically ultrafast dynamics (large amplitude jumps, coordinative oscillations, multiple gearing and antigearing rotation).

The discovery by PXRD and solid-echo ^2^H NMR of a disorder-to-order phase transition explains the intriguing framework-rotor modulation: above the phase transition bipy rotors are correlated to the framework dynamics in a collective swinging dance, while below the phase-transition bipy rings experience individual rapid 180° flip rotation in the static framework. At low temperature, the BCP rotor undergoes fast rotation by running over smooth energy landscape: single and multiple coordinated geared/antigeared rotations are switched-on in succession, as they explore discrete energy levels. Strikingly, a hyperfast geared motional mechanism with an energy barrier as low as 100 J mol^–1^ (24 cal/mol) was recognized in BCP rotor at temperatures between 1.6 and 10 K, showing the potential of the low-density crystals for converting the very low thermal energy into rotary motion. The active manipulation by chemical stimuli, such as DMF or CO_2_, which diffused from the liquid or gas phase into the porous matrix, resulted in the selective control over rotary dynamics.

Tuning juxtaposition and symmetry of hyperdynamic linkers in MOFs is a platform for the development of new coordinated rotors, switches, and machines in solids, the perspective being to realize stimuli-responsive materials with minimal energy dissipation.

## Supporting Information Available

Experimental details, including synthesis, crystallographic characterization, gas adsorption measurements, NMR results, molecular mechanics, and DFT calculations, (PDF)

## Accession Codes

CCDC 2069996–2069998 and 2070080 contain the supplementary crystallographic data for this paper. These data can be obtained free of charge via www.ccdc.cam.ac.uk/data_request/cif, or by emailing data_request@ccdc.cam.ac.uk, or by contacting The Cambridge Crystallographic Data Centre, 12 Union Road, Cambridge CB2 1EZ, UK; fax: +44 1223 336033.
