# Supplementary material for: Cascade Dynamics of Multiple Molecular Rotors in a MOF: Benchmark Mobility at a Few Kelvins and Dynamics Control by CO2
Source: J Am Chem Soc. 2021 Aug 13;143(33):13082–90. doi: 10.1021/jacs.1c03801 (PMC8413000; doi:10.1021/jacs.1c03801)
Supplement: Supplementary file 1 — ja1c03801_si_001.pdf [file ja1c03801_si_001.pdf]

Cascade Dynamics of Multiple Molecular Rotors in a MOF: Benchmark Mobility at a few Kelvins and Dynamics Control by CO_2

Jacopo Perego,[a] Charl X. Bezuidenhout,[a] Silvia Bracco,[a] Giacomo Prando,[b] Luciano Marchio',[c] Mattia Negroni,[a] Pietro Carretta,[b] Piero Sozzani*[a] and Angiolina Comotti* [a]

[a] Department of Materials Science, University of Milano Bicocca, Via R. Cozzi 55, 20125 Milan, Italy

[b] Department of Physics, University of Pavia, Via Bassi 6, 27100 Pavia, Italy

[c] Dipartimento di Scienze Chimiche, della Vita e della Sostenibilità Ambientale, Università degli studi di Parma, Parco Area delle Scienze 17/a, Parma, Italy.

INDEX

EXPERIMENTAL p. 3

SYNTHESIS p. 15

OPTICAL MICROSCOPY p. 16

THERMOGRAVIMETRIC ANALYSIS (TGA) p. 18

INFRARED SPECTROSCOPY (FT-IR) p. 18

^{1}H NMR IN SOLUTION p. 19

DIFFENTIAL SCANNING CALORIMETRY (DSC) p. 21

N_2 ADSORPTION ISOTHERMS p. 22

CRYSTAL STRUCTURES OF FTR-P1 AND FTR-P1d p. 23

^{13}C SOLID STATE NMR OF FTR-P1 AND FTR-P1d p. 31

^{2}H NMR OF FTR-P1d p. 39

^{1}H T_1 NMR RESULTS OF FTR-P1d p. 45

POTENTIAL ENERGY SCANS FOR THE BCP CONFORMATIONS AND ROTORS IN FTR-P1 p. 47

CO_2 ADSORPTION AND ADSORPTION-COUPLED CALORIMETRY IN FTR-P1 p. 49

POWDER X-RAY DIFFRACTION AND PXRD STRUCTURAL RIETVELD REFINEMENT of FTR-P1d·4CO_2 p. 63

VARIABLE TEMPERATURE POWDER X-RAY DIFFRACTION (VT-PXRD) UNDER 1 BAR CO_2 ATMOSPHERE p. 65

^{2}H NMR RESULTS FOR FTR-P1d·*x*CO_2 p. 68

PXRD AND ^{2}H NMR OF FTR-P1d*DMF p. 74

MOLECULAR MECHANICS (MM) 2D SCANS p. 75

COMPARISON BETWEEN DFT AND MOLECULAR MECHANICS 2D SCANS p. 77

RIETVELD REFINEMENT OF PXRD PROFILES p. 88

REFERENCES p. 95

EXPERIMENTAL

Optical microscopy. Optical images were acquired on a Leica DMLM optical microscope equipped with a Leica DFC280 color digital camera. Powder samples in DMF/MeOH mixtures were deposited on a glass slide with a Pasteur pipette and dried in air before measurement.

Thermogravimetric analysis (TGA). TGA were performed using a Mettler Toledo Star System 1 equipped with a gas controller GC10. Samples were previously outgassed overnight at 140 °C under high vacuum (10^{-3} mmHg) to remove adsorbed species. The experiments were conducted applying a thermal ramp from 30°C to 1000°C and a scan rate of 10 °C /min in dry air.

Differential scanning calorimetry (DSC). DSC were performed on a Mettler Toledo Stare DSC1 equipped with liquid nitrogen cooling system. Samples were previously outgassed overnight at 140 °C under high vacuum (10^{-3} mmHg) to remove adsorbed species. The experiments were run under nitrogen atmosphere in standard 40 µl Al pans. High temperature DSC measurements were performed under N_2 flow (80 mL/min) from 20 °C to 450 °C at a scan rate of 10 °C/min. Low temperature measurements were performed between – 130 °C and 130 °C (143.15 K and 403.15 K) at a scan rate of 10 °C/min.

Volumetric adsorption properties. N_2 adsorption isotherms at 77 K and CO_2 adsorption isotherms at 195 K, 273 K, 283 K, 293 K and 298 K were collected up to 1 bar using Micromeritics analyzer ASAP2020 HD. Samples were previously outgassed overnight at 140 °C under high vacuum (10^{-3} mmHg) to remove adsorbed species.

Gravimetric adsorption isotherms. Gravimetric CO_2 adsorption isotherms at (253 K, 273 K, 293 K) were collected on an Hiden Isochema up to 2 bar.

Infrared spectroscopy. Micro-FTIR spectra were measured on a Nicolet iN10 Infrared Microscope (Thermo Fisher Scientific) equipped with a liquid nitrogen cooled detector, working in reflection mode. The sample was degassed at 140 °C before the measurement and deposited on a gold substrate for analysis. Spectra were collected with a resolution of 4 cm^{-1} between 700 cm^{-1} and 4000 cm^{-1}.

Single Crystal X-ray crystallography. A summary of data collection and structure refinement for FTR-P1d is reported in Table S1. Single crystal data were collected with a Bruker D8 PhotonII area detector diffractometer (Mo Kα: λ = 0.71073 Å). Complete datasets were obtained by merging

several series of exposure frames collected at different temperatures between 275 and 110 K.[1] An absorption correction was applied with the program SADABS.[2] The structure were solved with ShelxT[3] and refined on F^2 with full-matrix least squares (ShelxL[4]), using the Olex2 software package.[5] According to variable temperature X-ray diffraction analysis, and DSC analysis, the system experiences a phase transition around 220 K. The inspection of the dataset collected at 275 K shows the presence of systematic extinctions consistent with a C lattice ($hkl_{extinct}$: h+k=2n+1). On the other hand, the data collection at 110 K (using the same unit cell metric) shows only a systematic weakness for the same class of reflections, pointing to the presence of a primitive lattice. The overall analysis of the systematic extinctions points to the *Cmma* space group (β phase) for the dataset collected at 275 K, and to the *Pcca* space group (α phase) for the 110 K dataset, respectively. For both α and β phases the unit cells were reoriented in order to adhere to the conventional space group notation (*Cmma* or *Pcca*). In the α phase, the central carbon atoms of the bicyclo(1.1.1)pentane (BCP) were found disordered in two positions, which were refined with 0.83(anisotropic)/0.17(isotropic) site occupancy factors. Moreover, one of the aromatic ring of the bipyridine was found disordered over two sites, which were refined with 0.74/0.26 site occupancy factors. The β phase exhibits the BCP fragment severely disordered by rotation along the vector linking the carboxylic functions. Six distinct images, belonging to two groups related by symmetry, could be refined. Within each group, the C and H atoms were refined with 0.12/0.16/0.22 site occupacy factors (0.5 overall for each group). The C atoms of BCP were refined with isotropic displacement parameters. In the β phase, the two aromatic rings of the bipyridine are disordered over two equivalent sites according to the presence of a crystallographic plane of symmetry. The hydrogen or deuterium atoms were placed at their calculated positions. CCDC deposition numbers 2069996, 2069997 and 2069998 for FTR-P1d at 110, 160 and 275 K, respectively, contain the supplementary crystallographic data for this paper.

Powder x-ray diffraction diffraction (PXRD). Powder X-ray diffraction experiments were performed on a Rigaku Smartlab SE equipped with a Cu Kα radiation (Kα1 = 1.540598 Å, Kα2 = 1.544426 Å, Kα ratio 0.5, Kαav = 1.541874 Å) operating at 40 KV and 30 mA with a Cu kβ radiation filter and a Hy-Pix 400 2D detector working in 1D detection mode. Data were collected under Bragg-Brentano geometry using a 2.5° incident soller slit and a 2.5° receiving slit over a range for 2θ of 3.0 – 60.0° with a step size of 0.02° and a scan speed of 1.0°·min^{-1}.

Variable temperature experiments were performed using an Anton Parr TTK 600 low temperature chamber operating with liquid nitrogen cooling system. The chamber is connected to a two-stage rotary pump and a gas pressure system and vacuum/pressure gauges to measure the pressure inside the chamber (Figure S1).

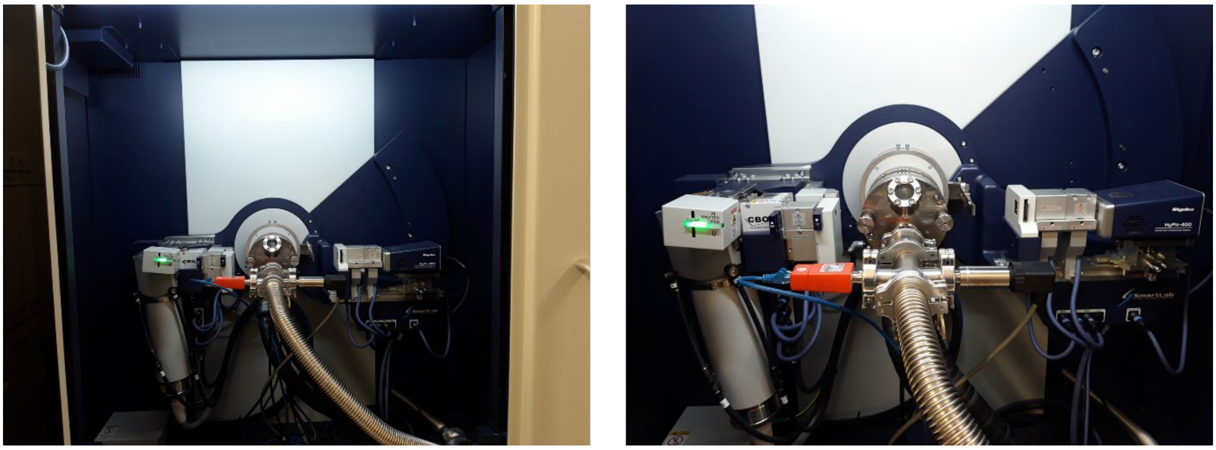

Figure S1. Digital images of the experimental set-up for vacuum/pressure PXRD.

Variable temperature powder X-ray diffraction (VT-PXRD). The sample was activated under high vacuum at 140°C and quickly loaded in the sample holder and inserted into the TTK 600 chamber. The chamber was evacuated (p = 1.5*10^{-2} Torr) and the sample was heated to 140°C. Vacuum measurements were performed under dynamic vacuum (p = 1.0*10^{-2} Torr); VT-PXRD experiments under constant pressure were performed introducing in the chamber a specific pressure of CO_2. A heating ramp with a loop cycle was programmed: the sample was cooled/heated at each target temperature at constant rate (10 K/min), equilibrated for 30 minutes and the PXRD pattern was collected (Scheme S1). The measurement were performed between 390 K and 210 K for vacuum collection and between 390 K and 250 K under CO_2 atmosphere.

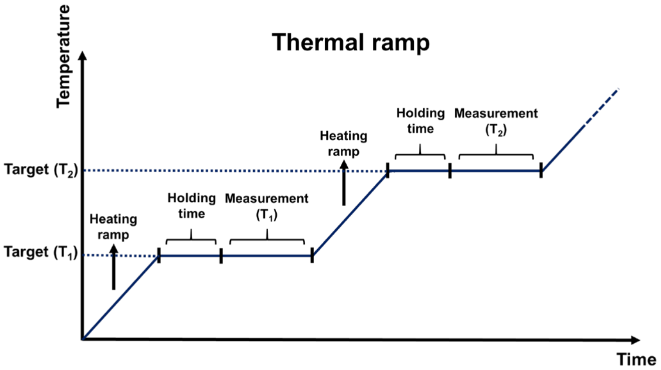

Scheme S1. *In-situ* variable temperature X-ray diffraction (VT-PXRD). The sample was heated under a constant heating rate up to target temperature (heating ramp, 10 K/min) and equilibrated for 30 minutes (holding time) before PXRD pattern collection at constant temperature.

Variable pressure powder X-ray diffraction (VP-PXRD). The sample was activated under high vacuum at 140°C and quickly loaded in the sample holder and inserted into the TTK 600 chamber. The chamber was evacuated (p = $1.5*10^{-2}$ Torr) and the sample was heated to 140°C. The sample was cooled to 293 K with a heating rate of -10 K/min under dynamic vacuum. At 293 K the vacuum was switched off and the first CO_2 amount was dosed inside the system (100 mbar). The sample was equilibrated for 30 minutes before PXRD pattern collection. The pressure was manually increased and the process was repeated for each pressure point (Scheme S2).

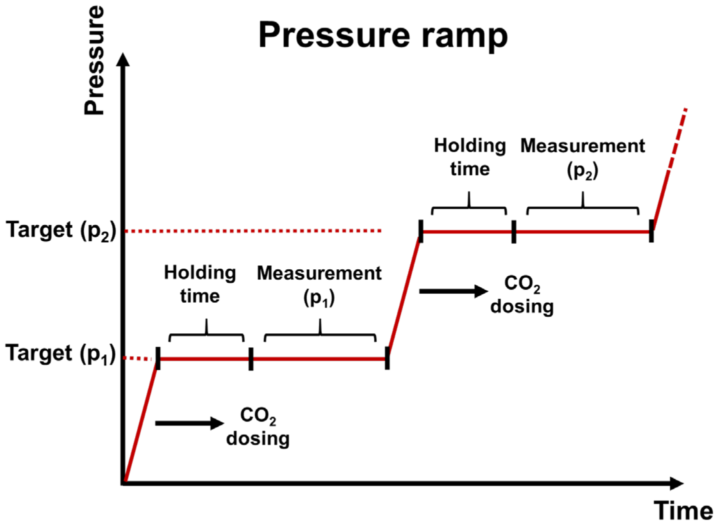

Scheme S2. *In-situ* variable pressure x-ray diffraction (VP-PXRD). A known pressure of CO_2 was loaded inside the TTK 600 chamber. The sample was equilibrated for 30 minutes after each dosing to reach the equilibrium and the PXRD data were collected. The process was repeated for each pressure point.

PXRD Structural Rietveld Refinement conditions. Indexing and Rietveld refinement were performed using the TOPAS-Academic-64 V6 software package.[6] The initial input structure used for the PXRD refinement was generated using the CASTEP code (DFT) within the Biovia Materials Studio software package.[7] The DFT optimizations had the unit-cell restrained to the PXRD indexed cell parameters while all the molecules could be optimized. Optimizations were performed using the GGA PBE functional with Grimme's DFT-D dispersion correction, thresholds for geometry optimization and SCF convergence were chosen as 2×10^{-6} eV. First, the unit-cell parameters where kept fix while all the atoms could optimize. This is followed by Rietveld refinement of the unit-cell parameters with all the atoms' fractional coordinates kept fix throughout the refinement process. The structure is cycled through the DFT optimization and Rietveld refinement until the structure showed no significant changes. The background was fitted and refined using a Chebyshev polynomial with 20 coefficients in the PXRD trace range from 3° to 60° 2theta with baseline shift refinement. Other

corrections include Specimen Displacement, Divergence Sample Length, Absorption with Sample Thickness Shape Intensity and Specimen Tilt. The peaks were fitted using a modified Thompson-Cox-Hastings pseudo-Voigt "TCHZ" profile. Preferred orientation was considered using a sixth-order Spherical Harmonics refinement. The accuracy of temperature controller were ±2° K in all explored range.

Structural two-phase Rietveld refinement was performed on all powder X-ray diffraction patterns of FTR-P1d under vacuum at variable temperatures and FTR-P1d·$x$$CO_2$ at variable pressure/temperature. The Rietveld refinement was carried out considering both phases (α- and β-phase) resulting in the percentage of α- and β-phase for FTR-P1d as function of temperature and for FTR-P1d·$x$$CO_2$ at variable CO_2 loadings.

CCDC deposition number 2070080 for FTR-P1d·4CO_2 contains the supplementary crystallographic data for this paper.

^{13}C Solid State NMR Spectroscopy. ^{13}C solid-state NMR experiments were carried out with a Bruker Avance 300 instrument operating at a static field of 7.04 T equipped with high-power amplifiers (1 kW) and a 4 mm double resonance MAS probe. ^{13}C{^{1}H} ramped-amplitude Cross Polarization (CP)[8] experiments were performed at 293 K at a spinning speed of 12.5 kHz using a recycle delay of 5 s and contact times of 2 and 0.05 ms. The experiments collected at 210 K were performed at a spinning speed of 8 kHz. The 90° pulse for proton was 2.9 μs. Quantitative ^{13}C{^{1}H} Single-Pulse Excitation (SPE) experiments were run using a 90° pulse of 4.6 μs and a recycle delay of 60 s. Crystalline polyethylene was taken as an external reference at 32.8 ppm from TMS. Phase-modulated Lee−Goldburg (PMLG) heteronuclear ^{1}H-^{13}C correlation (HETCOR) experiments coupled with fast magic angle spinning allowed the recording of the 2D spectra with a high resolution in both hydrogen and carbon dimensions.[9] Narrow hydrogen resonances, with line widths on the order of 1−2 ppm, were obtained with homonuclear decoupling during t_1; this resolution permits a sufficiently accurate determination of the proton species in the system. The 2D ^{1}H - ^{13}C PMLG HETCOR spectra were run with an LG period of 18.9 μs. The efficient transfer of magnetization to the carbon nuclei was performed by applying the RAMP-CP sequence. Quadrature detection in t_1 was achieved by the time proportional phase increments method (TPPI). The carbon signals were acquired during t_2 under proton decoupling by applying the two-pulse phase modulation scheme (TPPM).[10] The 2D ^{1}H-^{13}C PMLG HETCOR NMR spectra of PDR-3 were conducted at 298 K under magic-angle spinning (MAS) conditions at 12.5 kHz with contact times of 2, 1, 0.5, 0.1 and 0.05 ms.

Quantitative solid-state ^{1}H SPE MAS NMR spectra were performed with a Bruker Avance III 600 MHz instrument operating at 14.1 T, using a recycle delay of 20 s. A MAS Bruker probe head was

used with 2.5 mm ZrO_2 rotors spinning at 30 kHz. The 90° pulse for proton was 2.9 μs. The ^1H chemical shift was referenced to adamantane.

^2H NMR line shape. The deuterium NMR line shape is sensitive to the molecular motion and the time scale over which the motion occurs. In the absence of molecular motion, the frequency of a given deuteron is ruled by:

$$\omega = \omega_0 \pm \delta(3cos^2\theta - 1 - \eta sin^2\theta cos2\phi)$$

where ω_0 is the Zeeman frequency;

$\delta = \frac{3}{8}\frac{e^2qQ}{\hbar}$ ($\frac{e^2qQ}{\hbar}$ is the quadrupolar coupling constant);

η is the asymmetry parameter and the polar angle θ and ϕ specify the orientation of the magnetic field with respect to the principal axis of the electric field gradient tensor. The parameter η is usually zero for C-D bonds, meaning that the electric field gradient tensor is axially symmetric. If η is taken as zero, the NMR frequencies of the two transitions are given by:

$$\omega = \omega_0 \pm \delta(3cos^2\theta - 1)$$

This means that the frequencies of the NMR lines depend upon the angle θ formed between the C-D bond and the external magnetic field. In isotropic samples, the summation of the signals for all possible orientations gives rise the *static* Pake spectrum (Figure SXa), where the splitting d between the singularities is about 135 kHz for aromatic C-D bonds.

Molecular motion of *p*-phenylene moieties about their *para*-axis causes the lineshape to change in a way that depends on the geometry and time scale of the motion. If motion is considerably fast on the deuterium NMR time scale, the averaged NMR frequency is described by:

$$\omega = \omega_0 \pm \bar{\delta}(3cos^2\theta - 1 - \bar{\eta} sin^2\theta cos2\phi)$$

Where $\bar{\delta}$ and $\bar{\eta}$ are respectively the coupling constant and the asymmetry parameter for the averaged electric field gradient tensor. In this case $\bar{\eta}$ may be different from zero. Fast discrete reorientation by 180° flips around the *para*-axis results in the lineshape shown in Figure SXb. In fact, fast 180° flip yields an axially asymmetric spectrum ($\bar{\eta}$ = 0.6) with the splitting between singularities of about 34 kHz (d/4). In the intermediate regime of motion ($4*10^5$ Hz < k < $4*10^9$ Hz) the frequency of a single

NMR transition fluctuates between two values. For such rate constants deuterium NMR lineshapes can be predicted.[13]

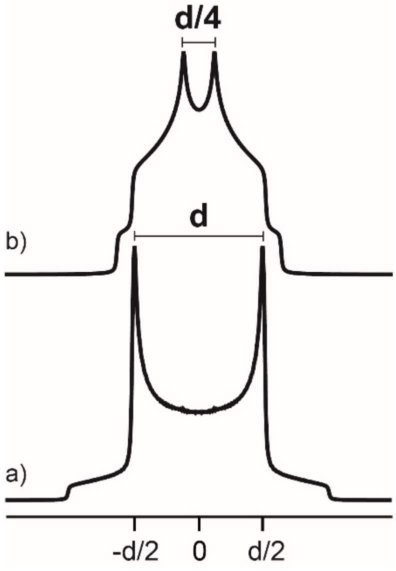

Figure S2. Theoretical deuterium NMR lineshapes for: a) a static C-D bond; b) a 180° phenyl ring flip.

Solid-state ^2H NMR spectroscopy experiments were performed on a Bruker 300 Avance spectrometer operating at a frequency of 46.07 MHz under a static magnetic field of 7.04 T, using a Bruker 5 mm wide-line probe. Fully relaxed spectra (15 s recycle delay) were acquired with the quadrupolar spin–echo pulse sequence, $(\pi/2)x - t_1 - (\pi/2)y - t_2$, with a pulse spacing of $t_1 = t_2 = 50$ μs. The stability and accuracy of the temperature controller (Bruker B-VT2000) were approximately ±0.2 K in all the explored range (150 K – 380 K). Theoretical simulations of ^2H NMR spectra were performed by the program Express 1.0[11] and NMR WEBLAB[12] with a quadrupolar coupling constant of 180 kHz and an asymmetry parameter of $\eta = 0.02$. The used Euler angles were β=61° and γ=0°; α was set accordingly with the motion: 4-site ±39.5°/±140.5° (-39.5°, 39.5°, 140.5°, -140.5°), 4-site ±28°/±152° (-28°, 28°, 152°, -152°), jump ±39.5° (-39.5°, 39.5°) and 2-site 180° (jump angle distribution σ=15°). The single simulations of jump mechanisms were than linearly combined to fit the experimental results.

The experiments were performed on the MOF containing perdeutero-bipy (FTR-P1d) under 150 mm torr of He in a sealed glass vial. The sample with CO_2 was loaded at 3.5 bar at 298 K. At low temperature (210 K) the estimated loading of the sample is equal to 1.70 mmol g^{-1}, corresponding to 95% of full loading. The value is almost constant until 291 K, then there is a progressive decrease in loading reaching the value of 1.13 mmol g^{-1} at 350 K that corresponds to 63% of full loading.

For the interpretation of the ^{2}H NMR spectra, we considered several rotational mechanisms. As an example for the interpretation of the four central singularities separated by 11.91 KHz and 37.86 KHz in experimental ^{2}H NMR spectra, we simulated them considering a 4-site mechanism and k=10^8 Hz as function of dihedral angle values of C-D bonds. The four central singularity values can be generated by considering rotational angles of ±39.5°/±140.5° (a full turn of 0, 79°, 180° and 259°). Other angles generated simulated profiles which do not match the experimental values. In Figure S3 and S4 the ^{2}H NMR lineshapes of different rotational angles at a fixed frequency and viceversa of variable frequencies at fixed rotational angles are reported, showing the high sensitivity of singularity separation values.

For the ±28° jumps we applied a similar procedure: we generated the lineshapes considering several rotational angles and frequencies and then selected the angle which generated the best match with the ^{2}H NMR profile.

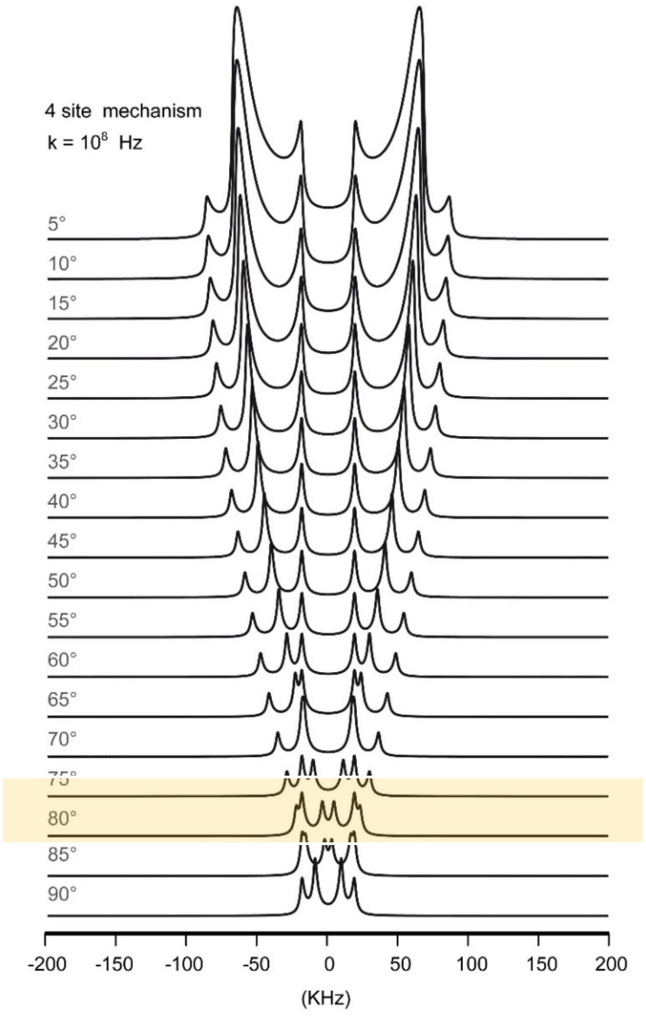

Figure S3. ^{2}H NMR spectra of C-D bond in an aromatic ring considering a 4-site reoreintational mechanism and k = 10^8 Hz. Each profile corresponds to a full turn of rotation with specific angles. As an example the angle value of 80° (orange line) indicates full rotation of C-D bond at ±40° and ±140°.

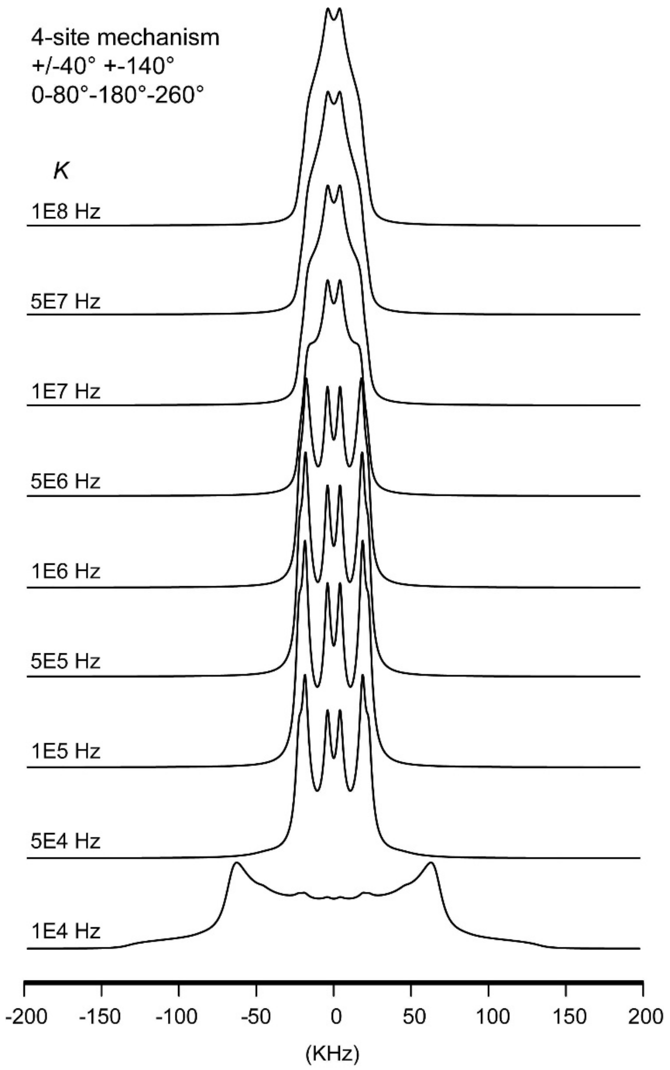

Figure S4. ^{2}H NMR spectra of C-D bond of an aromatic ring, considering a 4-site mechanism at fixed rotational angles and variable frequencies. Each profile corresponds to a full turn of rotation ±40° and ±140° (0-80°-180°-260°).

^{1}H T_1 relaxation times

We performed ^{1}H-NMR measurements using a home-made set-up based on an Apollo spectrometer (TecMag). We generated static magnetic fields $\mu_0H \leq 1.1$ T with an electromagnet (Bruker) and higher field values with a superconducting magnet (Cryomagnetics). We investigated the temperature window 5 K $\leq T \leq$ 300 K using a flux cryostat (Oxford Instruments) with liquid nitrogen and liquid helium as cryogenic liquids. Based on several previous calibrations on this cryostat using a control thermocouple on the probe close to the position of the sample, we associate an uncertainty ± 0.4 K to each temperature value. Additionally, we accessed temperatures 1.6 K $\leq T \leq$ 4.2 K using a static cryostat (International Cryogenics) by vapour pumping over a liquid helium bath. In these conditions,

we kept control on the temperature value by measuring the vapour pressure over the helium bath referring to the pressure-temperature phase diagram of helium. Based on several previous calibrations on this cryostat using a control thermocouple on the probe close to the position of the sample, we associate an uncertainty ± 0.1 K to each temperature value. We prepared an ad-hoc resonant RLC circuit with a coil, used to generate the alternating magnetic field and to detect the ^{1}H-NMR signal inductively, and a combination of fixed and variable capacitors aimed at the optimization of the tuning/matching of the overall impedance at the working frequency. We tailored the coil specifically for the samples – which were sealed in quartz tubes – aiming at the maximization of its filling factor. We quantified the spin-lattice relaxation time T_1 of the sample by means of the following conventional inversion recovery radiofrequency (RF) pulsed sequence.

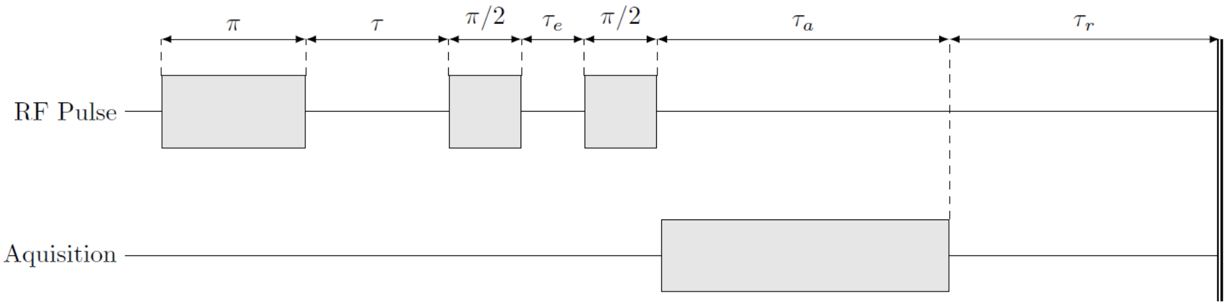

Scheme 3.

Here, π/2 and π represent RF pulses, whose effect is to tilt the nuclear magnetization away from the quantization axis by the indicated angle. Typically, the duration of π/2 pulses was around 2 - 3 μs. τ is a variable time with characteristic values ≈ 20 μs ≤ τ ≤ 100 s, whereas $\tau_e \approx$ 20 - 60 μs. We calibrated the idle time τ_r at all the temperature values in such a way that $\tau_r \geq 4\ T_1$. For each τ value, we numerically integrated the solid spin-echo developing after a time ≈ τ_e from the last π/2 RF pulse and plotted the resulting integral I as a function of τ in order to visualize the recovery of the nuclear magnetization towards the thermodynamical equilibrium condition (see the left-hand panel of Figure S5 for a representative example at fixed temperature for the sample under vacuum). We fitted the experimental data by means of the expression

$$I(\tau) = I(\infty)\left\{1 - 2f\exp\left[-\left(\frac{\tau}{T_1}\right)^{\beta}\right]\right\}. \quad (1)$$

Here, the parameter f ≤ 1 accounts for non-ideal inversion conditions, while the so-called stretching parameter β accounts for deviations of the recovery-law from the purely-exponential behaviour expected for spin-1/2 nuclei in homogeneous environments. Within the accessed experimental window, we measure values β ≥ 0.85 for the sample under vacuum, suggesting local homogeneous conditions and a negligible distribution of T_1 values. The situation is markedly different for the sample under CO_2 athmosphere. Here, for T < 160 K, we measure sizeably lower values of β with a non-trivial dependence of this quantity on temperature. Moreover, for temperatures T < 90 K, the fitting quality based on Eq. 1 decreases considerably, as shown in the right-hand panel of Figure S5. For the sample under CO_2 atmosphere we obtained a much better description of the experimental recovery curves using the fitting function

$$I(\tau) = I_f(\infty)\left\{1 - 2f_f \exp\left[-\left(\frac{\tau}{T_{1f}}\right)\right]\right\} + I_s(\infty)\left\{1 - 2f_s \exp\left[-\left(\frac{\tau}{T_{1s}}\right)\right]\right\}, \tag{2}$$

i.e., a multi-exponential recovery with two components (here labelled as *f* and *s*) for all the temperature values T < 160 K. We have no evidence of the multi-exponential character of the recovery for T > 160 K, so we used Eq. 1 for the fitting procedure also for the sample under CO_2 atmosphere in this temperature limit.

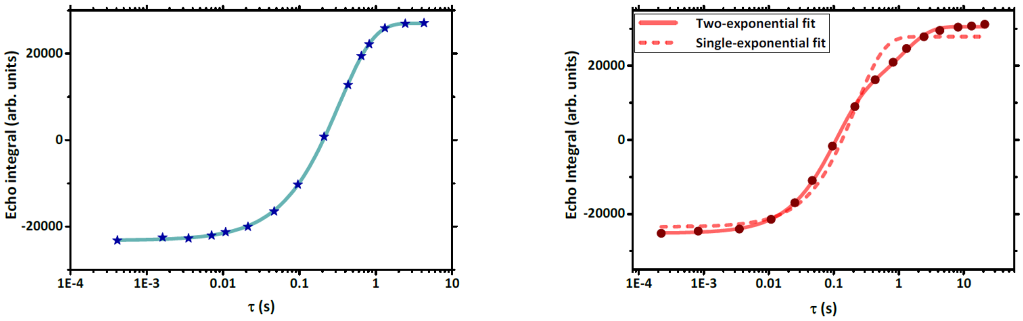

Figure S5. The left-hand panel shows the recovery of the nuclear magnetization in a representative T_1 experiment at T = 1.6 K and $\mu_0H \approx$ 0:38 T for the sample under vacuum. The continuous line is a best-fitting curve according to Eq. 1. The right-hand panel shows the recovery of the nuclear magnetization in a representative T_1 experiment at T = 75 K and $\mu_0H \approx$ 1.08 T for the sample under CO_2 athmosphere. The continuous lines are best-fitting curves according to Eqs. 1 and 2 (dashed and continuous lines, respectively).

The ^1H T_1 relaxation times as function of temperature were fitted with the linear combination of six Kubo-Tomita equations:

$$\frac{1}{T_1} = \sum_{n=1}^{6} (KT)_n \qquad (KT)_n = C_n \left(\frac{\tau_{C_n}}{1+\omega^2 \tau_{C_n}^2} + 4 \frac{\tau_{C_n}}{1+4\omega^2 \tau_{C_n}^2} \right) \qquad \tau_{C_n} = \tau_{0_n} \, e^{E_{A_n}/RT} \tag{3}$$

Where τ_{0_n} is the maximum rotation period, E_{A_n} is the activation energy and C_n is the relaxation constant of the nth component and ω the angular Larmor frequency of the nucleus.[13]

Computational Details. Atomic coordinates were imported from the refined crystal structures. All atoms in the frameworks were optimized as part of a periodic system using the CASTEP module of the Materials Studio software suite. The unit-cell parameters, as determine by indexing the PXRD traces, were kept fixed during geometry optimization process. The optimizations were performed using the GGA PBE functional with Grimme's DFT-D dispersion correction, and thresholds for geometry optimization and SCF convergence were chosen as 2×10^{-6} eV. Single point energy calculations were performed using the GGA PBE functional with Grimme's DFT-D dispersion correction, and threshold for SCF convergence were chosen as 1×10^{-6} eV.

Potential energy scans for the BCP conformations and rotors. Potential energy scans were performed using the GAUSSIAN16 software available through the CINECA high performance computing centre.[14]

Molecular Mechanics (MM) 2D scans

Atomic charges for Molecular Mechanics MM calculations. Single point calculations were performed using the GGA PBE functional with Grimme's DFT-D dispersion correction (CASTEP module of the Materials Studio software suite); thresholds for SCF convergence were chosen as 1×10^{-6} eV. The Milliken charges were calculated at the end of the SCF cycle. These atomic charges were used in all the Molecular Mechanics and Molecular Dynamics calculations.

Molecular Mechanics (MM). All MM calculations were performed using the Forcite-Plus module using a modified Dreiding ForceField with an Ewald summation method for both Electrostatics and van der Waals. Only the torsional potential described by *–C_31–C_R–* were modified to fit the rotational potential of the BCP rotor more accurately (Figure S3). The total rotational barrier for an isolated rotor is 48 cal/mol as a six-fold potential. Since there are six of these tortional forcefield parameters applied to one rotor, the value was set 8 cal/mol.

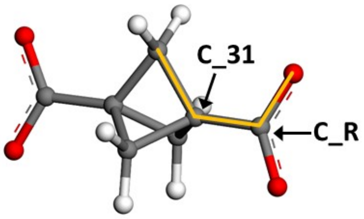

Figure S6. The torsional forcefield parameter, *–C_31–C_R–*, that was modified. The molecule contains six equivalent torsional forcefield parameters, but only one is indicated for clarity.

SYNTHESIS

Dimethylformamide (DMF), methanol (MeOH), 4,4'-bipyridine and 4,4'-bipyridine-d_8 (98 % atom D) were purchased by Merck. Bicyclo[1.1.1]pentanedicarboxylic acid was purchased from Fluorochem.

FTR-P1 synthesis. Bicyclo[1.1.1]pentanedicarboxylic acid (96.8 mg; 0.62 mmol), 4,4'-bipyridine (48.4 mg; 0.31 mmol) and $Zn(NO_3)_2{\cdot}6H_2O$ (184.4 mg; 0.62 mmol) were dispersed at room temperature in DMF (14 mL) and MeOH (21 mL) in a glass vials. The mixture was sonicated for 1 minute and then heated at 85°C in a preheated oven for 24 hours. Large crystals growth on the walls and bottom of the vial. Once cooled to room temperature the crystals were filtered and washed with a 3:2 mixture of MeOH/DMF and then with fresh MeOH. The powder was dried in air and then activated under vacuum at 140°C for 8 hours.

FTR-P1d synthesis. The same procedure for FTR-P1 was followed but using 4,4'-bipyridine-d_8 (50.9 mg; 0.31 mmol) instead of 4-4'-bypiridine.

OPTICAL MICROSCOPY

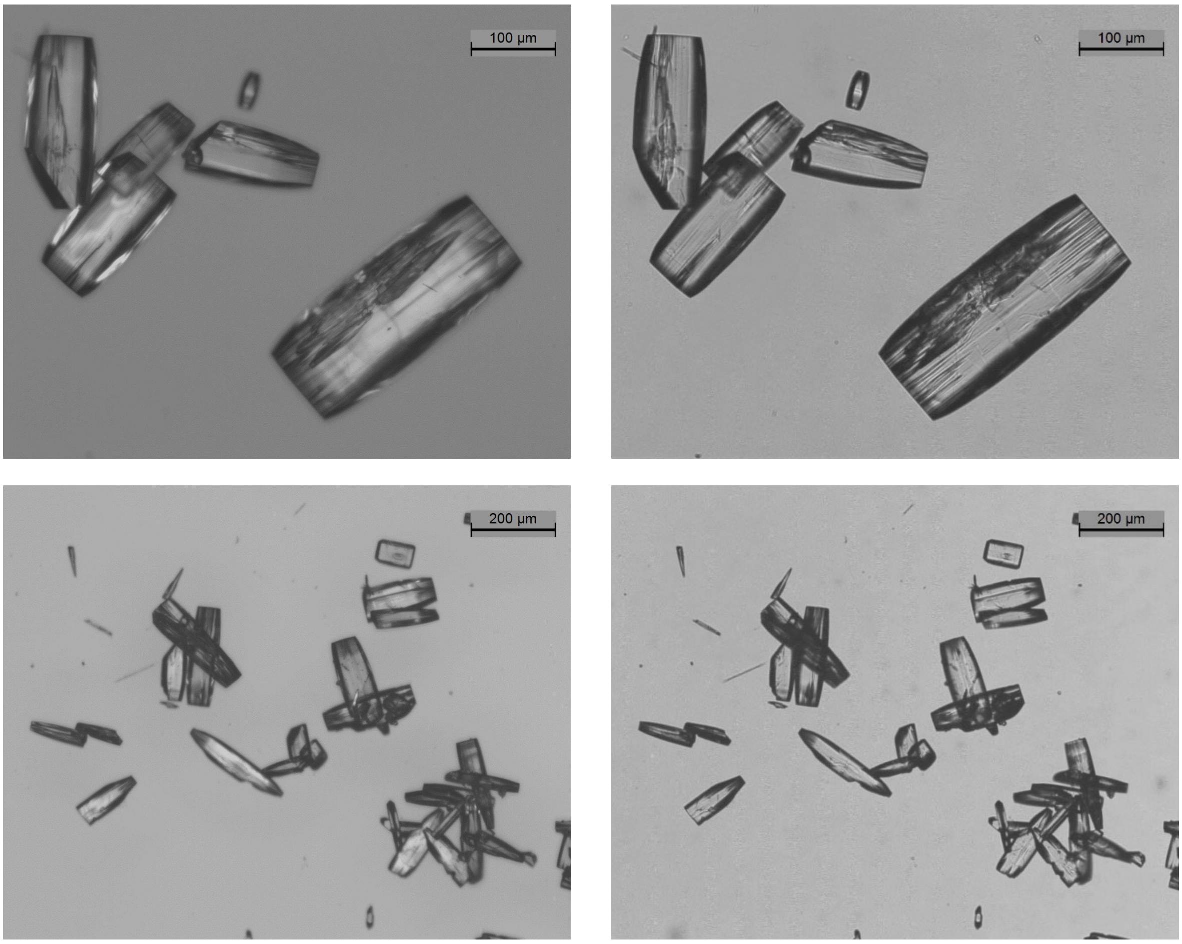

Figure S7. Optical images of FTR-P1d crystals at different magnifications. The images were collected using incident light (left images) and transmitted light (right).

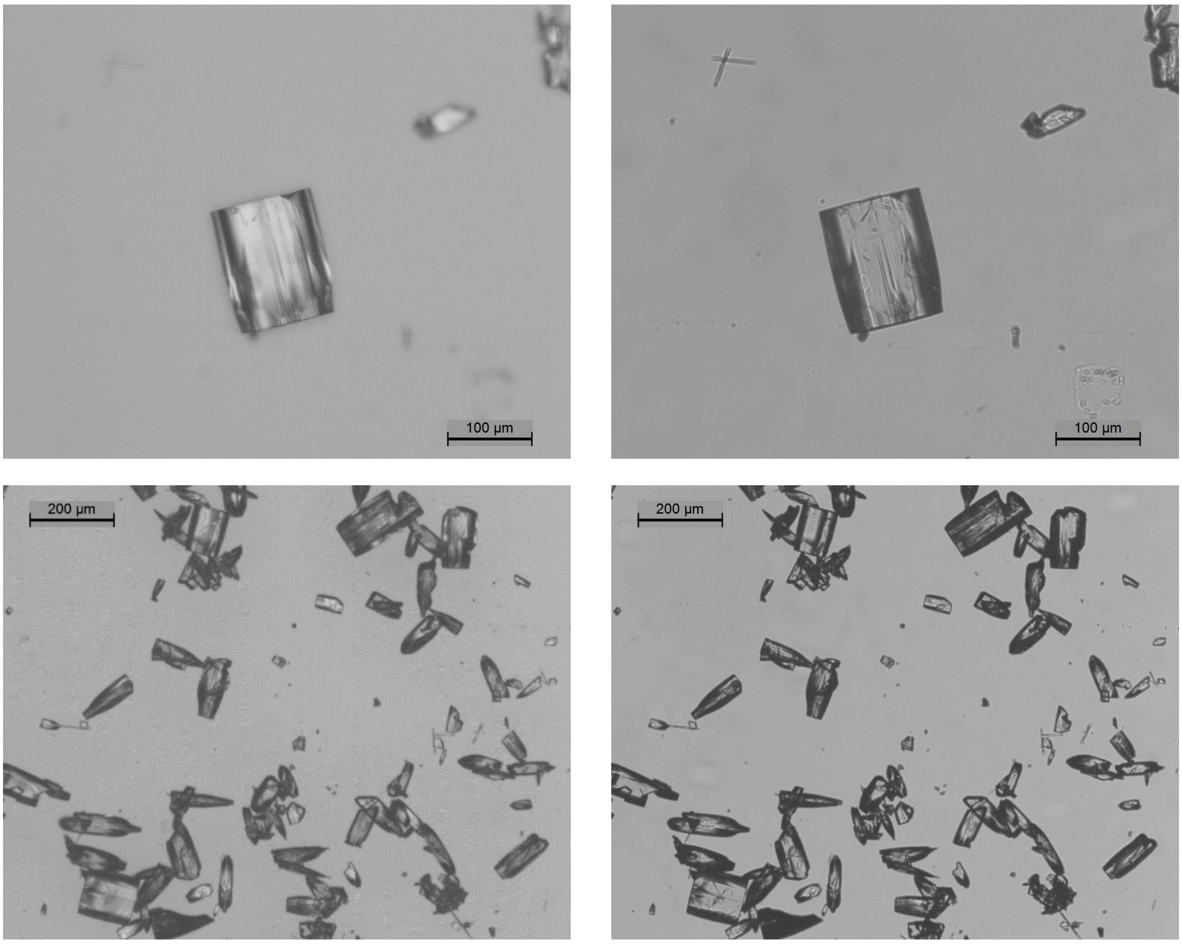

Figure S8. Optical images of FTR-P1d crystals at different magnifications. The images were collected using incident light (left images) and transmitted light (right).

THERMOGRAVIMETRIC ANALYSIS (TGA)

FTR-P1 and FTR-P1d were treated under high vacuum at 140°C to remove moisture and guest species. Thermogravimetric analyses were performed under oxidative conditions (dry air, 50 mL/min) from 30 to 1000°C.

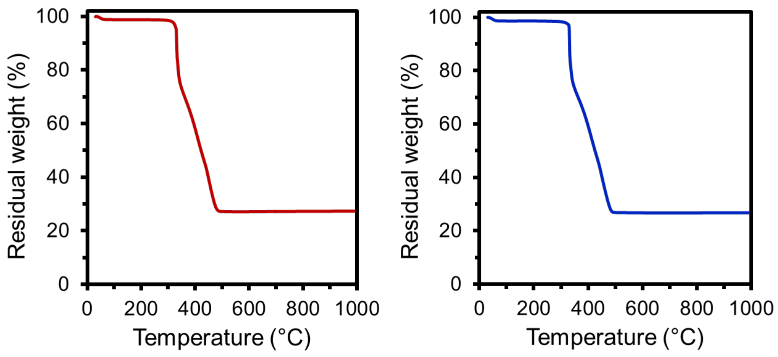

Figure S9. Thermogravimetric analysis of FTR-P1 (red) and FTR-P1d (blue) collected between 30 to 1000°C under oxidative atmosphere.

The TGA trace displayed a sharp weight loss at 330°C. Powder X-ray diffraction on the white residue after thermal analysis confirmed the generation of pure ZnO. Experimental residual weight at 1000°C of 26.7 $\%_{wt}$ and 27.3 $\%_{wt}$ for FTR-P1 and FTR-P1d respectively, were in excellent agreement with the theoretical values of 27 $\%_{wt}$ and 27.2 $\%_{wt}$.

INFRARED SPECTROSCOPY (IR)

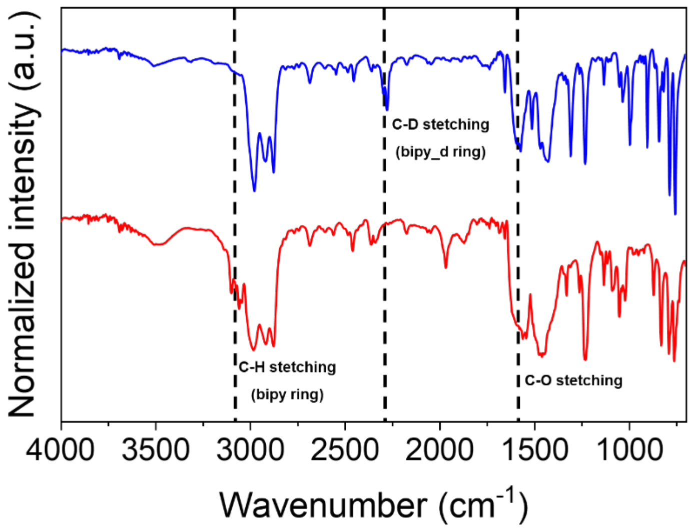

Figure S10. Infrared spectra of FTR-P1 (red, bottom) and FTR-P1d (blue, top) collected between 4000 and 700 cm^{-1} at room temperature. The carboxylic acid unit of free BCP carboxylic acid resonating at 1664 cm^{-1} shifted to 1596 cm^{-1} after coordination with Zn^{++} ions (in FTR-P1 structure).

^{1}H NMR OF DIGESTED SAMPLES

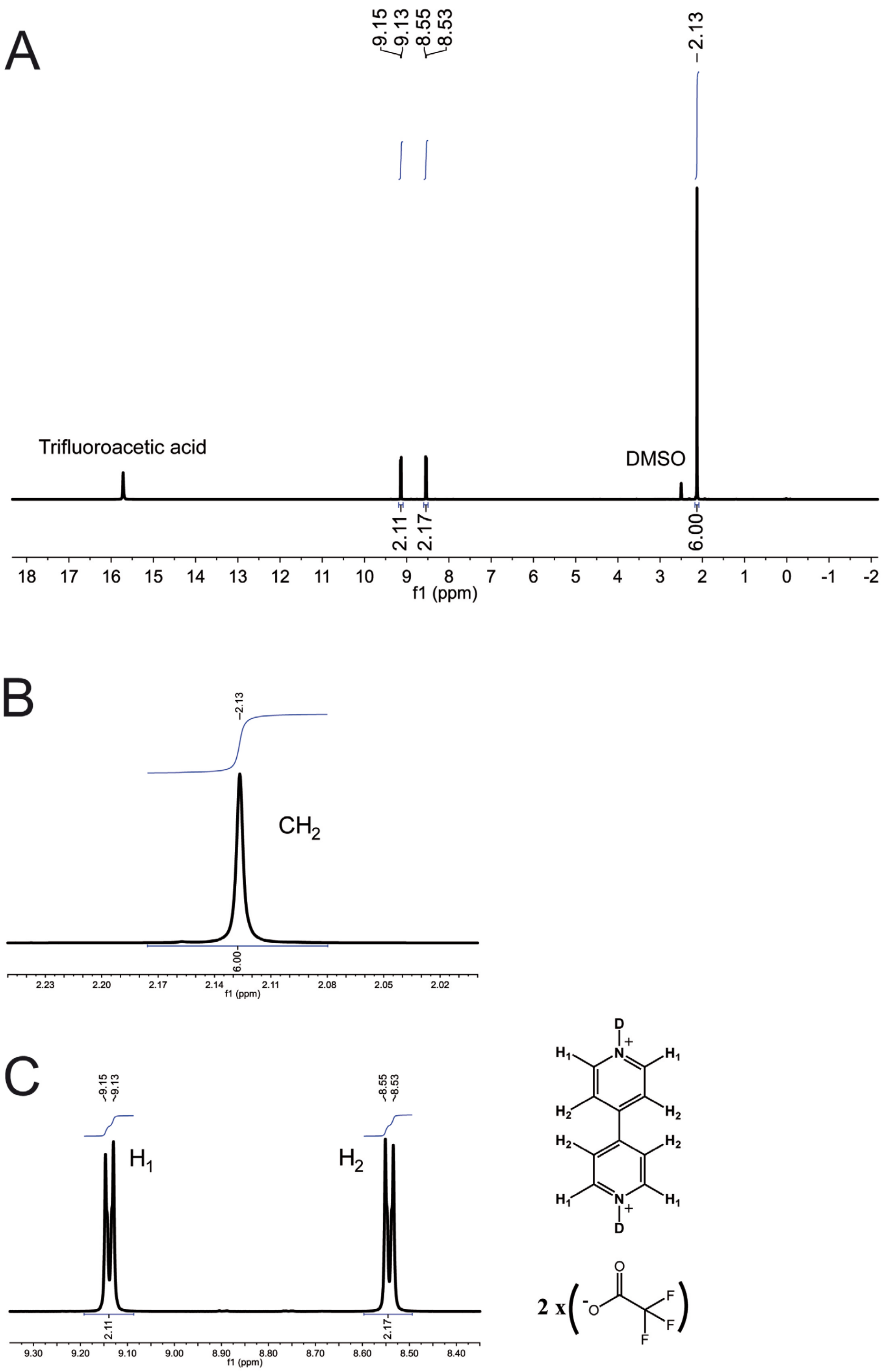

Figure S11. A) ^{1}H liquid NMR of activated FTR-P1 after digestion in a mixture of deuterated trifluoroacetic acid and dimethylsulfoxide. The signal at 2.13 ppm was assigned to the methylene groups of bicyclo[1.1.1]pentanedicarboxylic acid units; the doublets centered at 9.14 ppm and 8.54 ppm were assigned to the α and β hydrogens of the 4,4'-bipyridinium salt moieties (hydrogen H_1 and H_2, respectively). B) and C) Enlargement between 2.0 ppm and 2.25 ppm and between 8.35 ppm and 9.35 ppm.

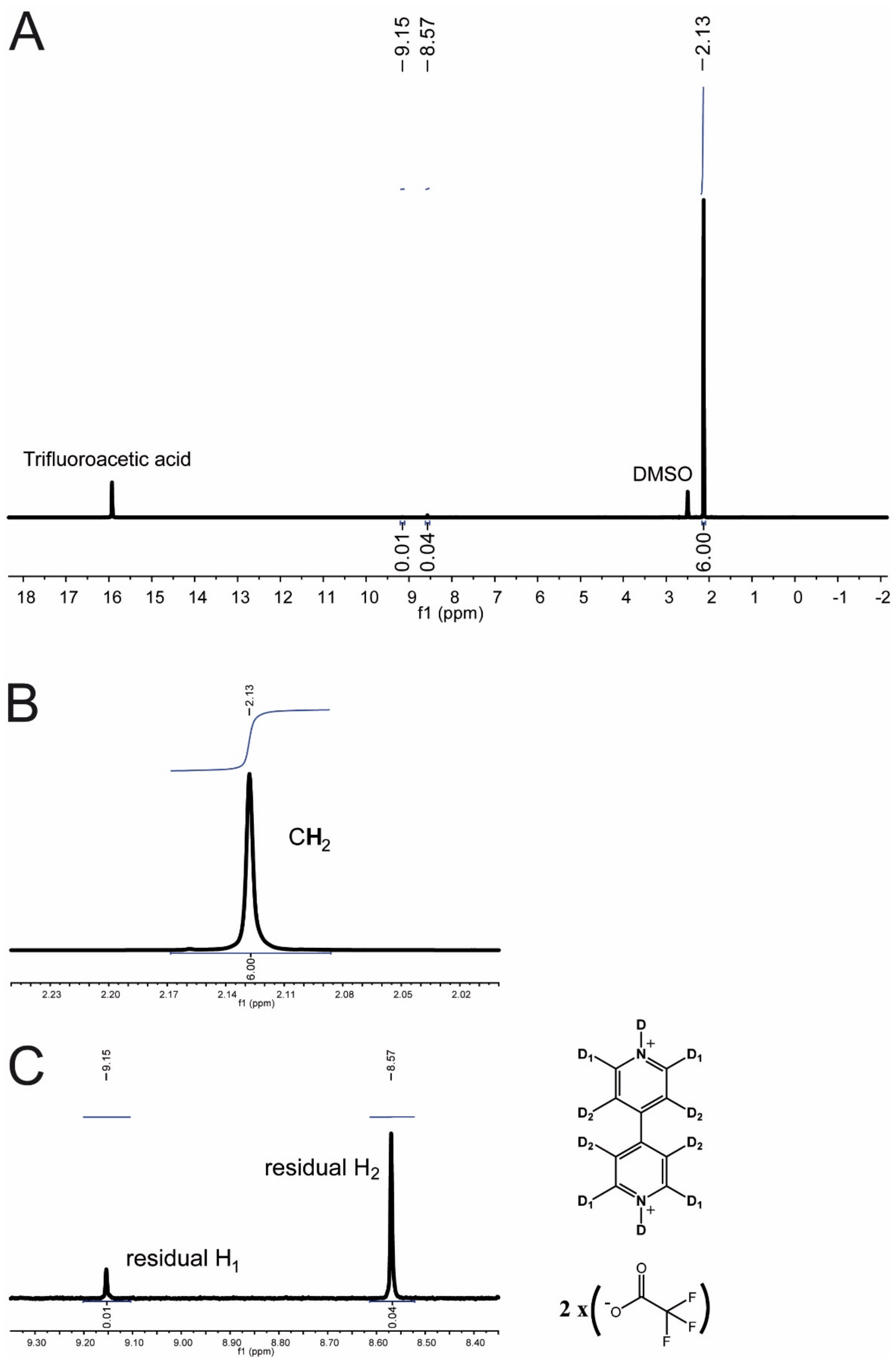

Figure S12. A) ^1H liquid NMR of activated FTR-P1d after digestion in a mixture of deuterated trifluoroacetic acid and dimethylsulfoxide. The signal at 2.13 ppm was assigned to the methylene groups of bicyclo[1.1.1]pentanedicarboxylic acid units; the signals at 9.15 ppm and 8.57 ppm were assigned to the residual α and β hydrogens of the 4,4'-bipyridinium salt moieties (residual hydrogen H_1 and H_2, respectively). B) and C) Enlargement between 2.0 ppm and 2.25 ppm and between 8.35 ppm and 9.35 ppm.

DIFFERENTIAL SCANNING CALORIMETRY (DSC)

High temperature analysis

FTR-P1 and FTR-P1d were treated under high vacuum at 140°C to remove moisture and guest species. The samples were quickly loaded in a 40 µL aluminium pan and inserted in the sample holder. Differential scanning calorimetry were performed under nitrogen atmosphere (nitrogen, 80 mL/min) from 30 to 450°C.

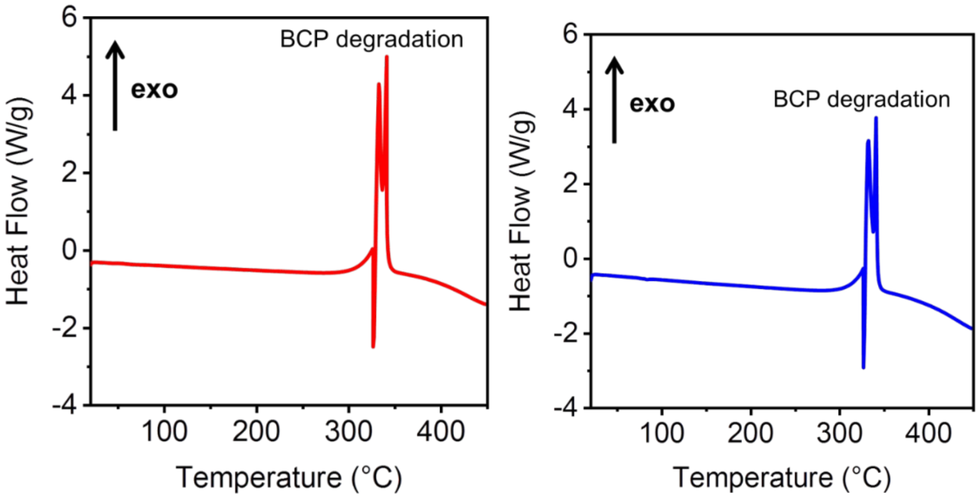

Figure S13. Differential scanning calorimetry of FTR-P1 (red) and FTR-P1d (blue) collected between 20°C and 450°C (blue) under an inert atmosphere (nitrogen, 80 mL/min).

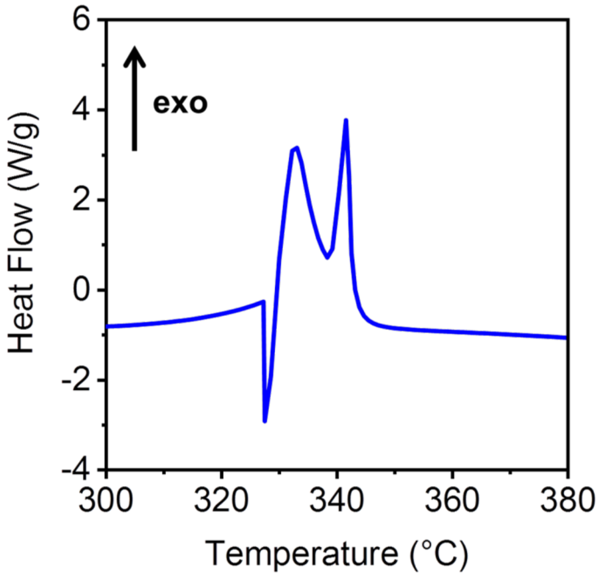

Figure S14. Enlargement of the differential scanning calorimetry of FTR-P1d (blue) collected between 300°C and 380°C (blue) under an inert atmosphere (nitrogen, 80 mL/min). The degradation of the bicycle[1.1.1]pentane moiety generates an exothermic event at 332°C and 342°C.

N_2 ADSORPTION ISOTHERMS

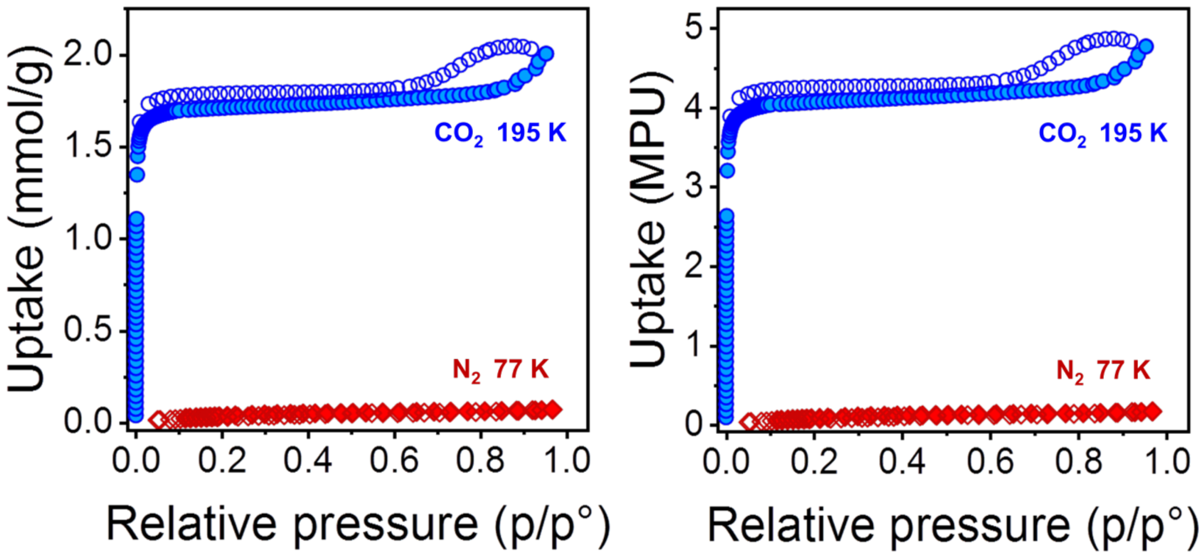

Figure S15. CO_2 adsorption isotherms measured at 195 K (blue circles) and N_2 adsorption isotherm collected at 77 K (red diamonds) for FTR-P1. The uptake values are expressed in mmol/g (left) and MPU (right). Filled and empty symbols denote adsorption and desorption branches, respectively. FTR-P1 displayed a type I CO_2 adsorption isotherm with a maximum uptake of 1.79 mmol/g STP (4.02 MPU), measured at 0.8 p/p°.

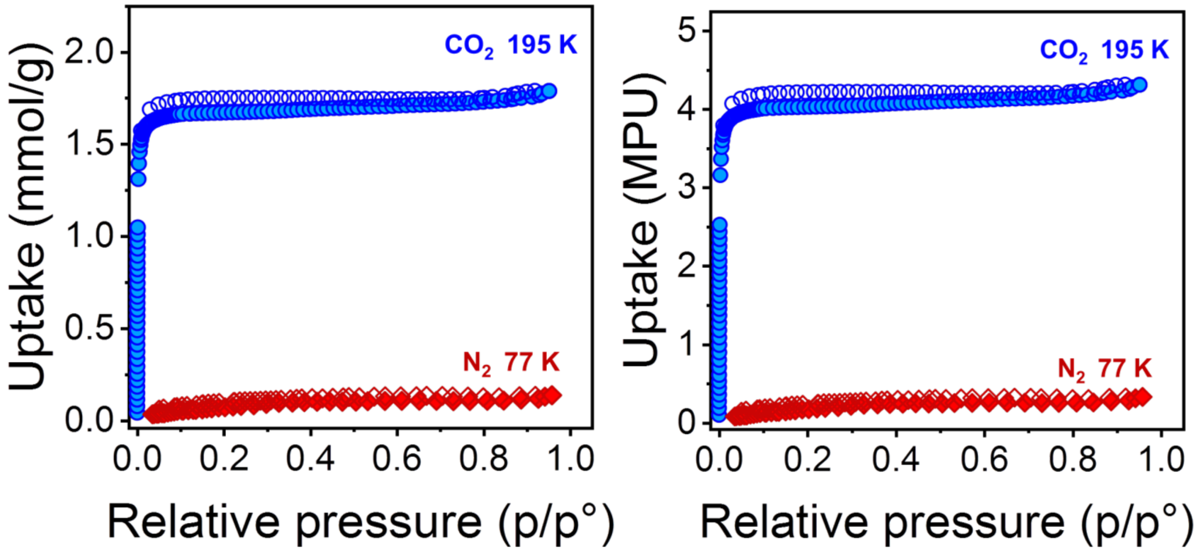

Figure S16. CO_2 adsorption isotherm measured at 195 K (blue circles) and N_2 adsorption isotherm collected at 77 K (red diamonds) for FTR-P1d. The uptake values are expressed in mmol/g (left) and MPU (right). Filled and empty symbols denote adsorption and desorption branches, respectively. FTR-P1d displayed a type I CO_2 adsorption isotherm with a maximum uptake of 1.73 mmol/g STP (4.15 MPU), measured at 0.8 p/p°.

CRYSTAL STRUCTURES OF FTR-P1 AND FTR-P1d

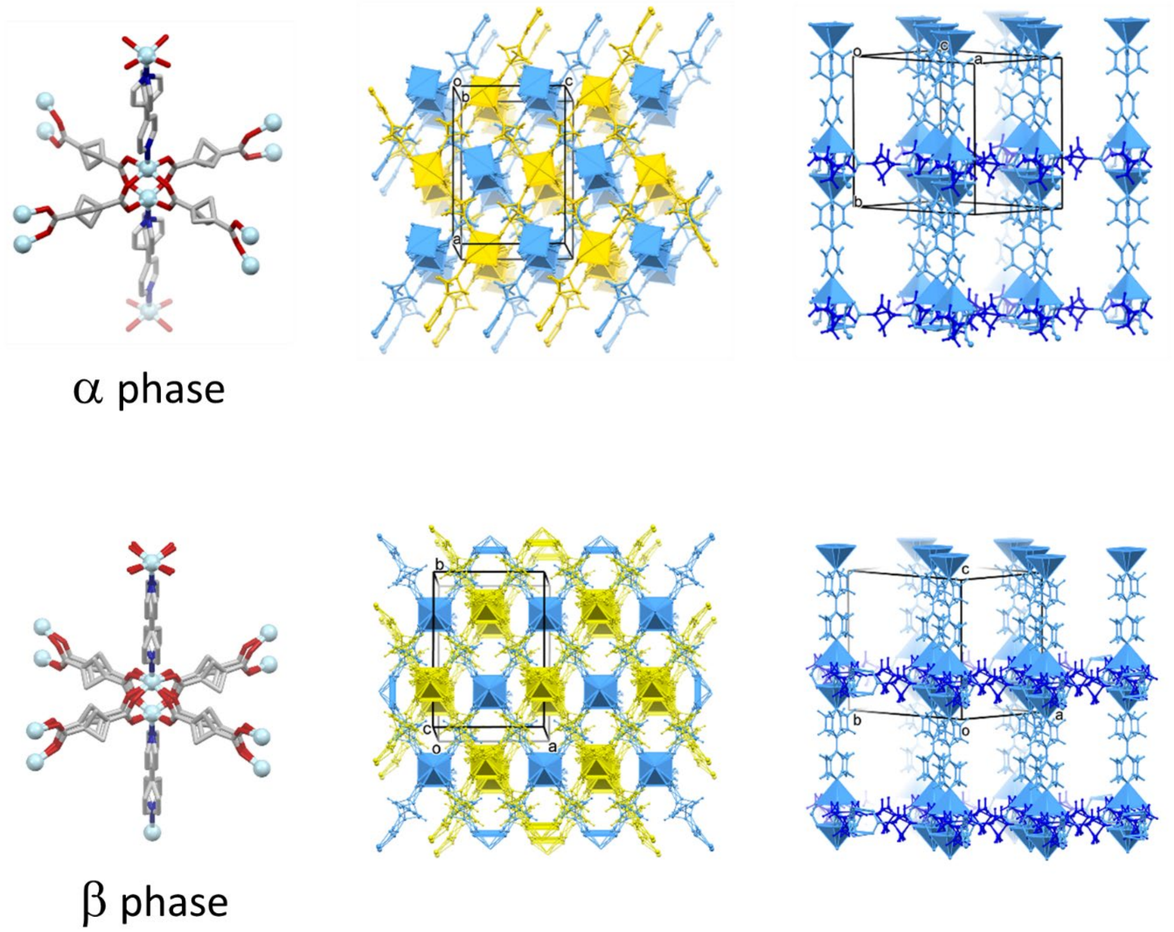

Figure S17. Comparison between the structural arrangements between the α and β phases. Left, dinuclear zinc node; Middle, depiction of the two independent networks, blue and yellow. The metal atoms are represented as solid tetrahedron. Right, highlight on the connections between linkers and nodes within a single network (BCP in dark blue).

The overall arrangement of both α and β phases can be described as a reticular system having a dinuclear zinc node, and two different linkers, namely a 4,4'-bipyridine and a bicyclo[1.1.1]pentane-1,3-dicarboxylic acid (BCP). The β phase comprises two equivalent BCP networks that shares the metal nodes. The less symmetric α phase presents only one of the β phase networks. A further distinction between the two phases is represented by the slightly different disorder of the bipyridine rings. In both phases, the metal centers exhibit a distorted square pyramidal geometry with the apical position occupied by the nitrogen atom of the bipyridine and the equatorial position occupied by the oxygen atoms of four symmetry related carboxylate moieties. The overall architecture comprises two interpenetrated networks that extend in a plane by means of the bridging bis-carboxylic groups of

BCP, and they expand perpendicularly to this plane by virtue of the 4,4'-bipy-zinc interaction. The crystal packing of both the α and β phases shows the presence of columnar cavities that run parallel to column aligned with the bipyridine linker, and correspond to approximately 7% and 9% of the unit cell volume, respectively (probe radius 1.2 Å, contact surface), Figure S12.

Table S1. Crystal data and structure refinement for FTR-P1d at the indicated temperatures.

Empirical formula	$C_{24}H_{12}D_8N_2O_8Zn_2$	$C_{24}H_{12}D_8N_2O_8Zn_2$	$C_{24}H_{12}D_8N_2O_8Zn_2$
Formula weight	603.21	603.21	603.21
Temperature/K	110.0	160.0	275.0
Crystal system	orthorhombic	orthorhombic	orthorhombic
Space group	Pcca	Pcca	Cmma
a/Å	16.1420(7)	16.1552(5)	11.4471(3)
b/Å	14.0673(6)	14.0647(3)	16.1419(6)
c/Å	11.3786(4)	11.4005(3)	14.0687(5)
α/°	90	90	90
β/°	90	90	90
γ/°	90	90	90
Volume/Å^3	2583.79(18)	2590.40(12)	2599.59(15)
Z	4	4	4
ρ_{calc}g/cm^3	1.551	1.547	1.541
μ/mm^{-1}	1.905	1.901	1.894
F(000)	1208.0	1208.0	1208.0
Crystal size/mm^3	0.14 × 0.14 × 0.1	0.14 × 0.14 × 0.1	0.23 × 0.2 × 0.19
Radiation	MoKα (λ = 0.71073)	MoKα (λ = 0.71073)	MoKα (λ = 0.71073)
2Θ range for data collection/°	5.048 to 55.01	5.246 to 51.436	5.048 to 51.428
Index ranges	-20 ≤ h ≤ 20, -18 ≤ k ≤ 18, -14 ≤ l ≤ 14	-19 ≤ h ≤ 19, -16 ≤ k ≤ 17, -13 ≤ l ≤ 13	-13 ≤ h ≤ 13, -19 ≤ k ≤ 19, -17 ≤ l ≤ 17
Reflections collected	49570	18983	31842
Independent reflections	2987 [R_{int} = 0.0667, R_{sigma} = 0.0243]	2458 [R_{int} = 0.0615, R_{sigma} = 0.0317]	1351 [R_{int} = 0.0490, R_{sigma} = 0.0166]
Data/restraints/parameters	2987/9/198	2458/9/187	1351/27/169
Goodness-of-fit on F^2	1.043	1.034	1.048
Final R indexes [I>=2σ (I)]	R_1 = 0.0264, wR_2 = 0.0633	R_1 = 0.0302, wR_2 = 0.0691	R_1 = 0.0187, wR_2 = 0.0471
Final R indexes [all data]	R_1 = 0.0417, wR_2 = 0.0701	R_1 = 0.0516, wR_2 = 0.0787	R_1 = 0.0215, wR_2 = 0.0496
Largest diff. peak/hole / e Å^{-3}	0.42/-0.34	0.32/-0.36	0.30/-0.27

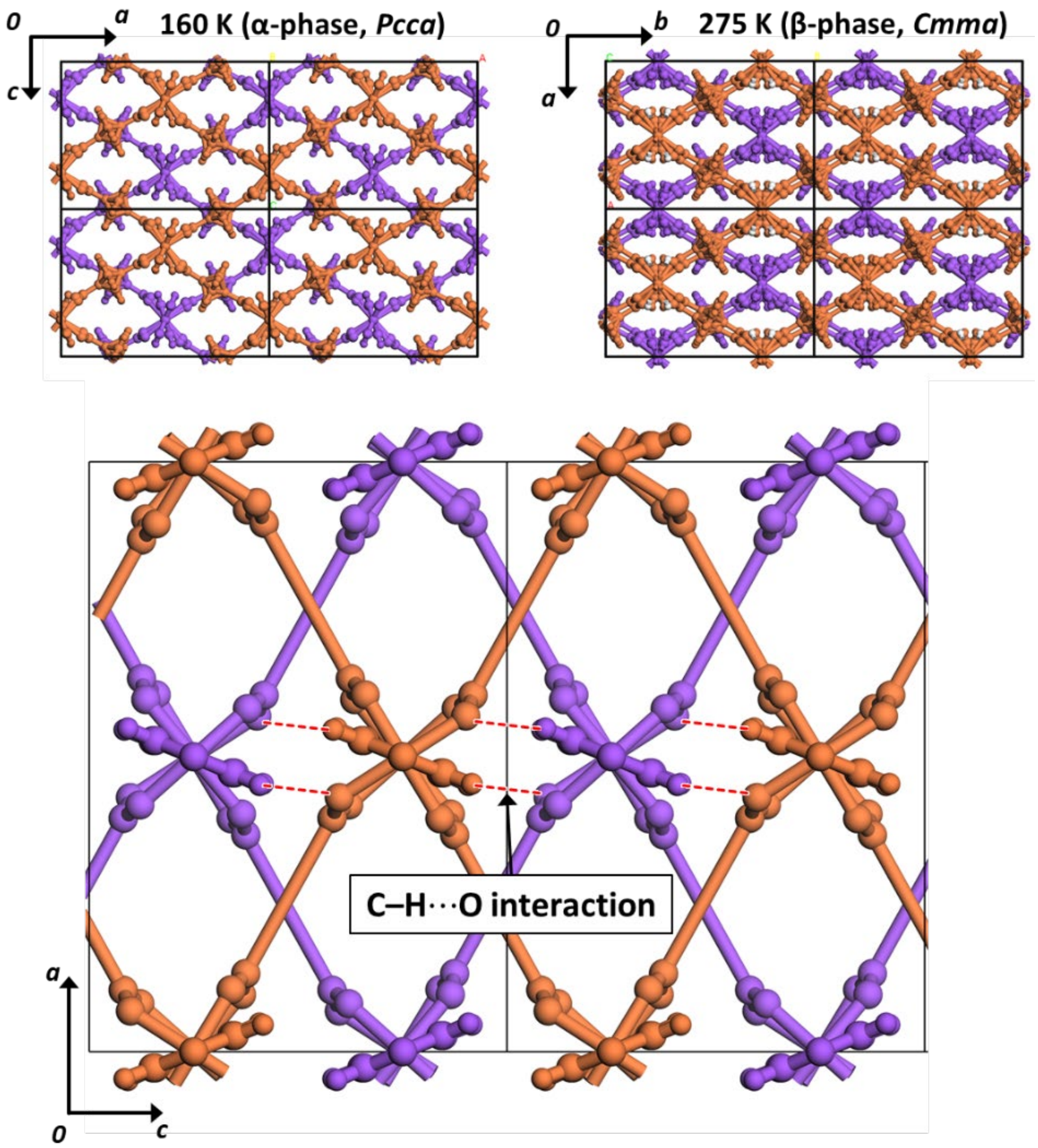

Figure S18. Top: Crystal structure of FTR-P1 viewed down the *c*-axis for the α-phase (left) and β-phase (right). showing the two interpenetrated networks highlighted in orange and violet. Bottom: Crystal structure of FTR-P1 highlighting the C-H···O interaction. The BCP moieties are represented as bars.

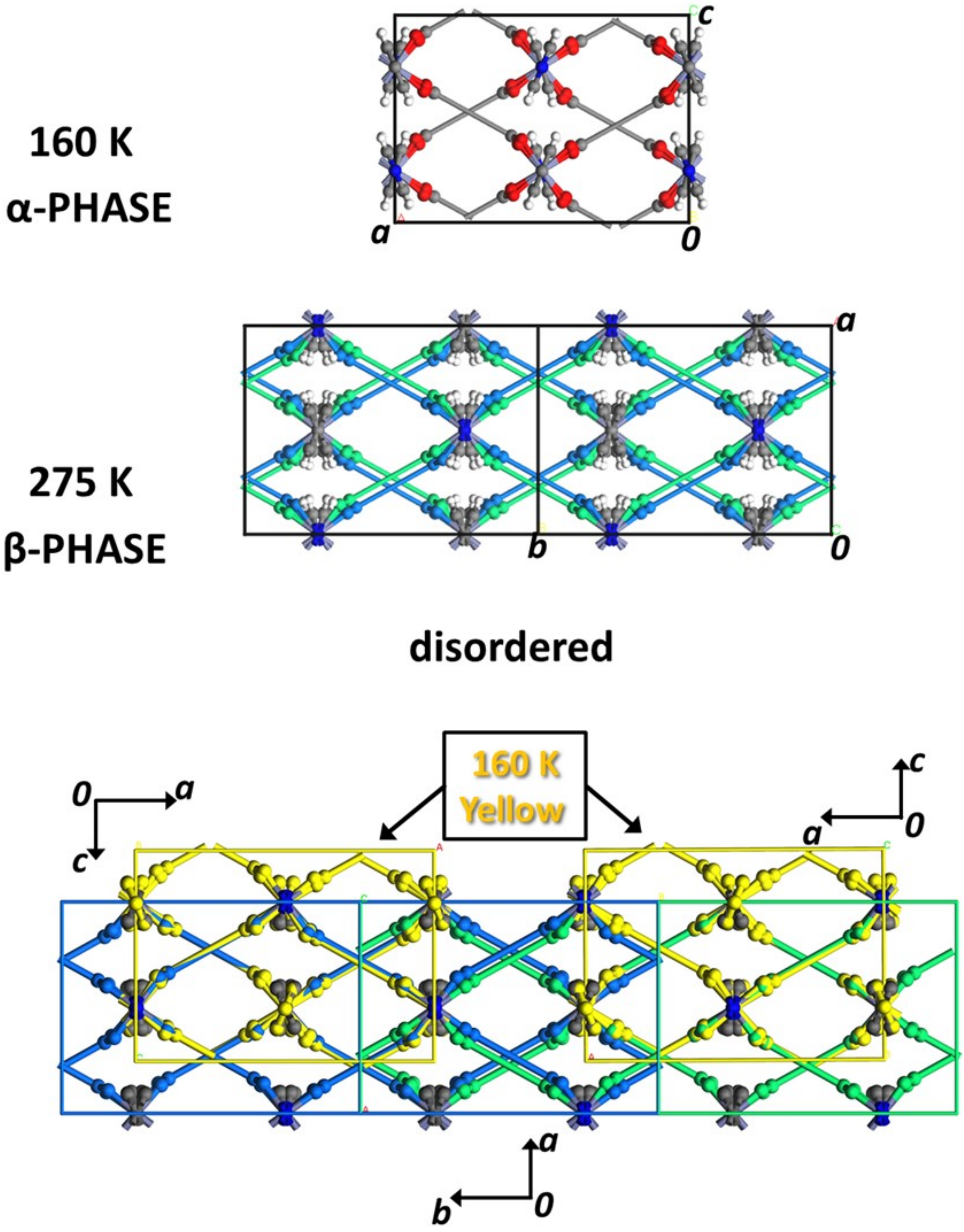

Figure S19. Top: Low-Temp phase, α-phase collected at 160 K. Middle: High-temp phase, β-phase collected at 275 K. The 2-site disorder of the BCP moiety in the 275 K structure is shown in green and blue. Bottom: Both the green and blue structures at 275 K (β-phase) can be overlayed with the 160 K structure (α-phase) in yellow. This illustrates that the structures generated by the blue and green disorder are identical.

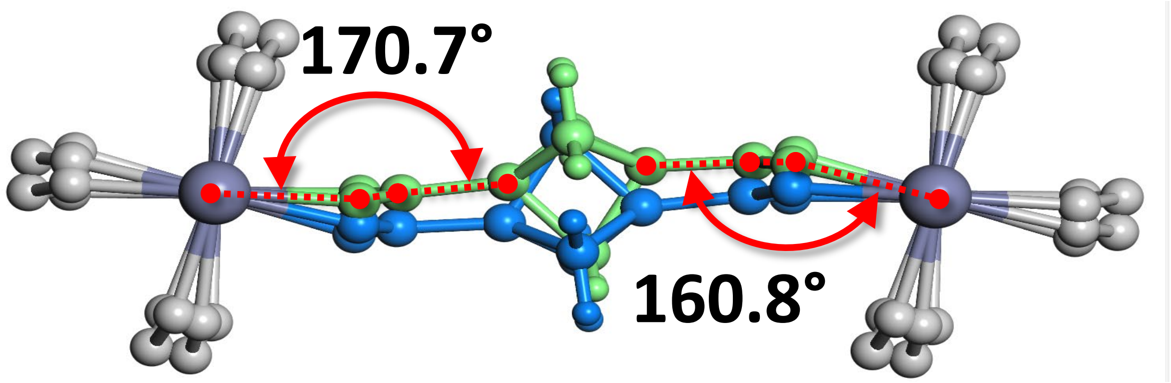

Figure S20. The BCP molecule is disordered over two symmetry equiv. positions. The carboxylates of the BCP molecule coordinates to the Zn metal in two distanced ways which yields an angle of 170.7° for one and 160.8° for the other.

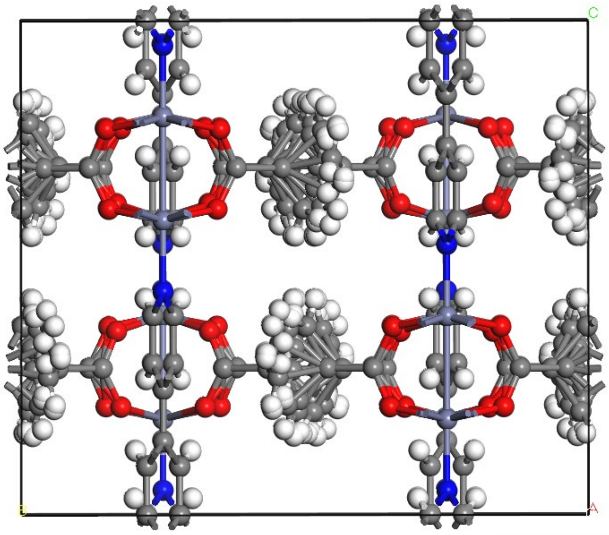

Figure S21. FTR-P1 activated structure 275 K (*Cmma)* viewed down the *a*-axis. The unit-cell of the FTR-P1 structure contains 8 bcp ligands and 4 bipy ligands. The unit-cell contains only one unique BCP and bipy from which the rest is symmetry generated.

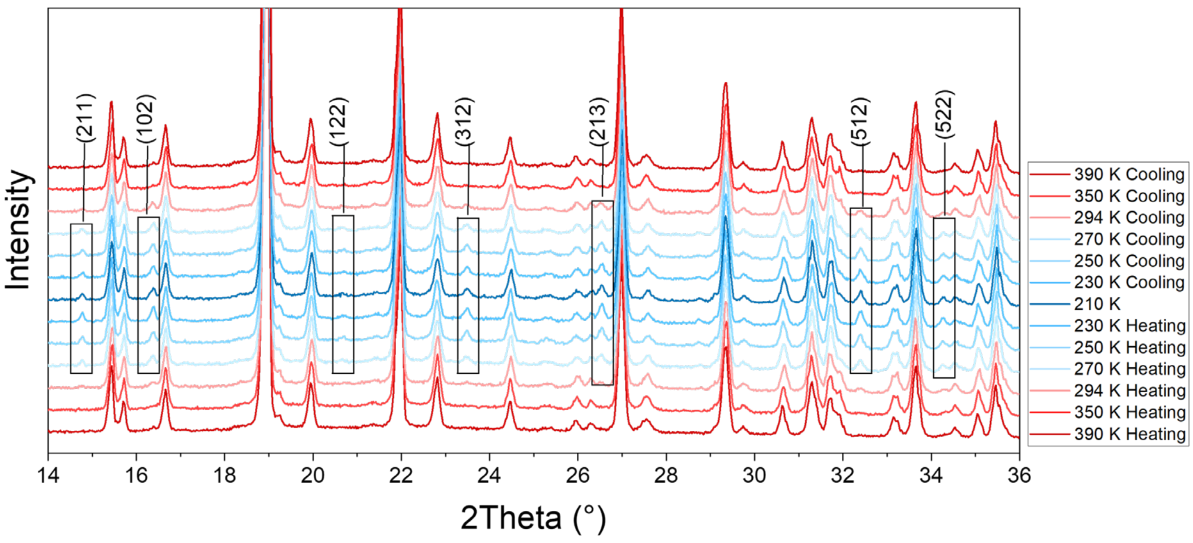

Figure S22. VT-PXRD cycle for FTR-P1d under vacuum with a temperature range of 390 – 210 – 390 K. The red traces represent the β-phase while the α-phase is shown in blue. The additional peaks associated with the α-phase are highlighted with the black box and their corresponding miller indices.

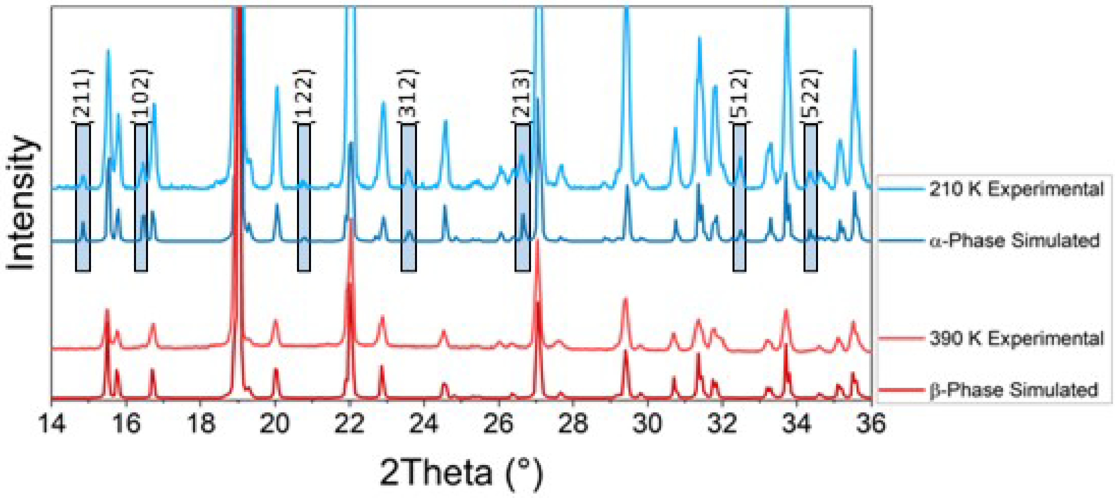

Figure S23. Simulated P-XRD plots of both the α-phase and β-phase compared with the experimental P-XRD traces collected under vacuum at 210K and 390K for **FTR-P1d**. The high temperature XRDs are shown in red while the low temperature XRDs are shown in blue. The Miller indexes indicate the reflections that are present for the *Pcca* α-phase but absent in the *Cmma* β-phase. Indeed, in the α-phase the extinction hkl with ***h+l=2n+1*** (for a conventional C lattice) is significantly more evident. The appearance of peaks at these indexes indicates the presence of the α-phase.

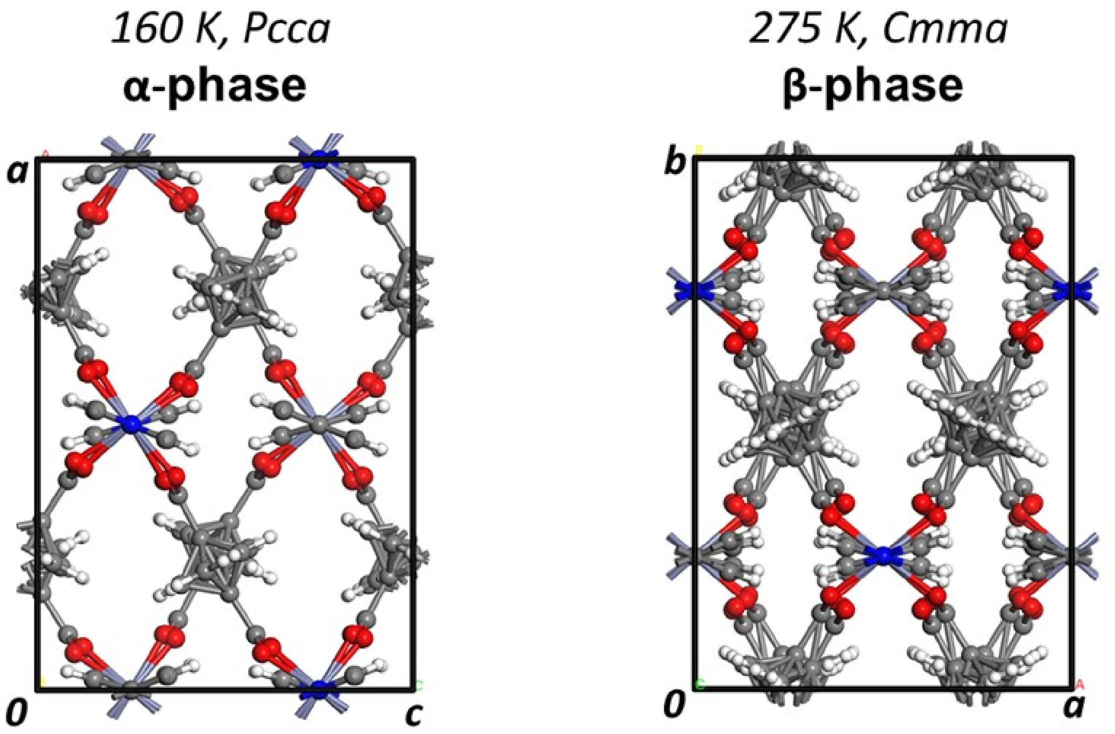

Figure S24. The α-phase (160 K, *Pcca*) and β-phase (275 K, *Cmma*) models FTR-P1d used for the two-phase Rietveld refinement of PXRD traces collected under vacuum and varying temperatures.

Table S2. The results of the phase-quantification of the α-phase (blue diamonds, *Pcca*) and β-phase (red circles, *Cmma*) of FTR-P1d from the two-phase Rietveld refinement of PXRD traces collected under vacuum and varying temperatures. The total error in the phase percentage, Rwp and Rp of the final Rietveld refinement cycle are shown.

Temperature (K)	Phase %		Error	Rwp (%)	Rp (%)
	α-Phase	β-Phase			
390	5.08	94.92	1.9	8.6	5.9
350	17.9	82.1	2.2	10.2	7.2
294	27.03	72.97	2.8	10.0	7.1
270	38.41	61.59	3.2	8.74	6.5
250	57.9	42.1	2.7	8.6	6.4
230	62.5	37.5	2.4	8.0	6.1
210	67.6	32.4	2.3	7.8	5.8
210	66.51	33.49	1.8	8.5	6.4
230	64.2	35.8	3.1	8.6	6.5
250	56.8	43.2	3.1	8.6	6.4
270	32.45	67.55	2.3	8.1	6.0
294	25.15	74.85	2.6	8.8	6.3
350	18.9	81.1	2.4	10.5	7.6
390	5.08	94.92	1.9	8.6	5.9

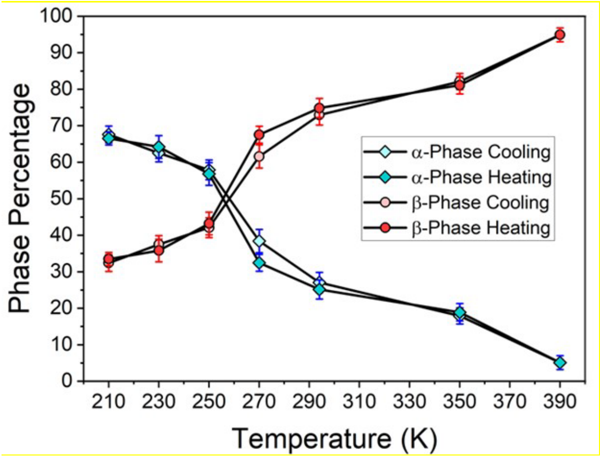

Figure S25. Left - A plot of the phase-quantification of the α-phase (blue diamonds, *Pcca*) and β-phase (red circles, *Cmma*) of FTR-P1d from the two-phase Rietveld refinement of PXRD traces collected under vacuum and varying temperatures. The error bars are shown in blue for the α-phase and in red for the β-phase.

^{13}C SOLID STATE NMR OF FTR-P1 AND FTR-P1d

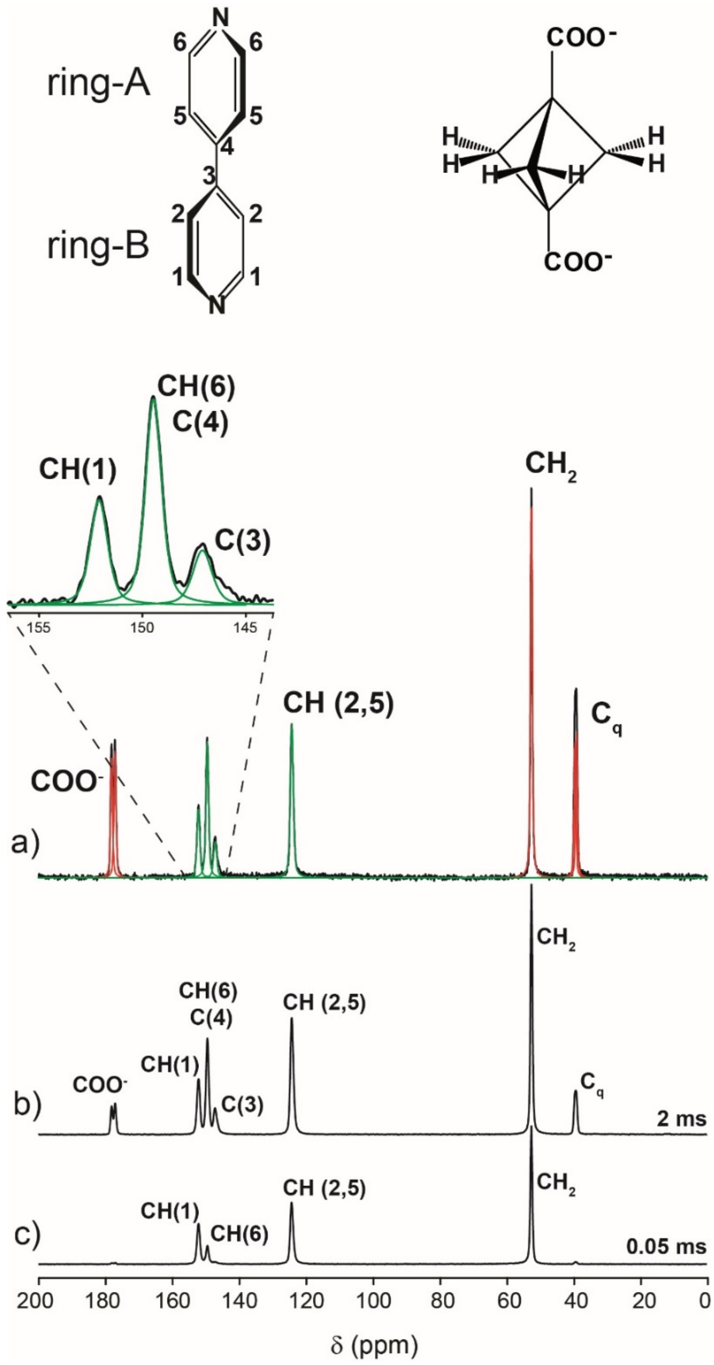

Figure S26. ^{13}C MAS NMR analysis of FTR-P1 performed at 293 K at a spinning speed of 12.5 kHz: a) Quantitative ^{13}C{^{1}H} SPE spectrum collected a recycle delay of 60 s; ^{13}C{^{1}H} CP spectra collected with contact time of 2 ms (b) and 0.05 ms (c).

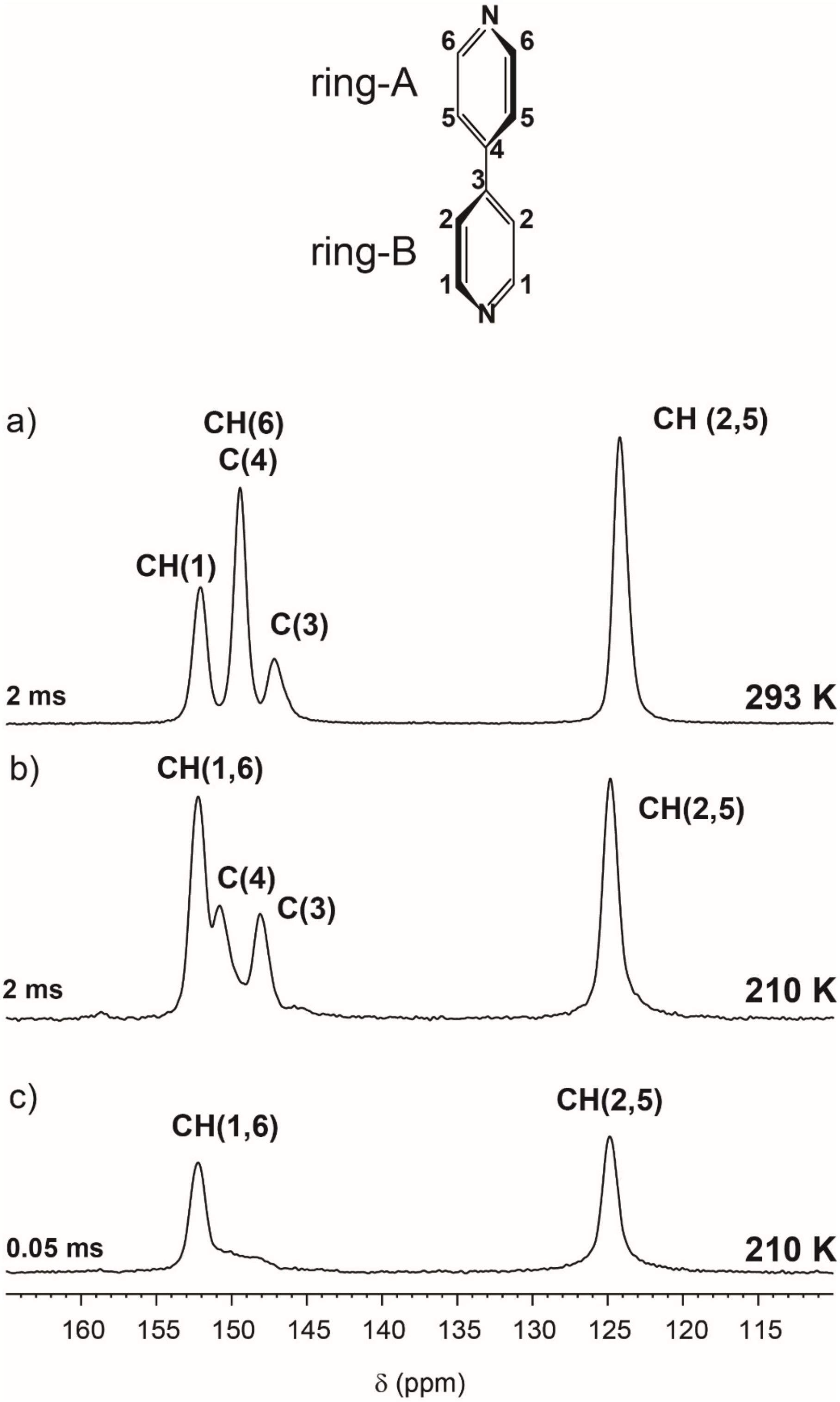

Figure S27. $^{13}C\{^{1}H\}$ CP MAS spectra of FTR-P1 collected at a spinning speed of 8 kHz and contact time of 2 ms at 293 K (a); contact time of 2 ms at 210 K (b) and contact time of 0.05 ms at 210 K (c).

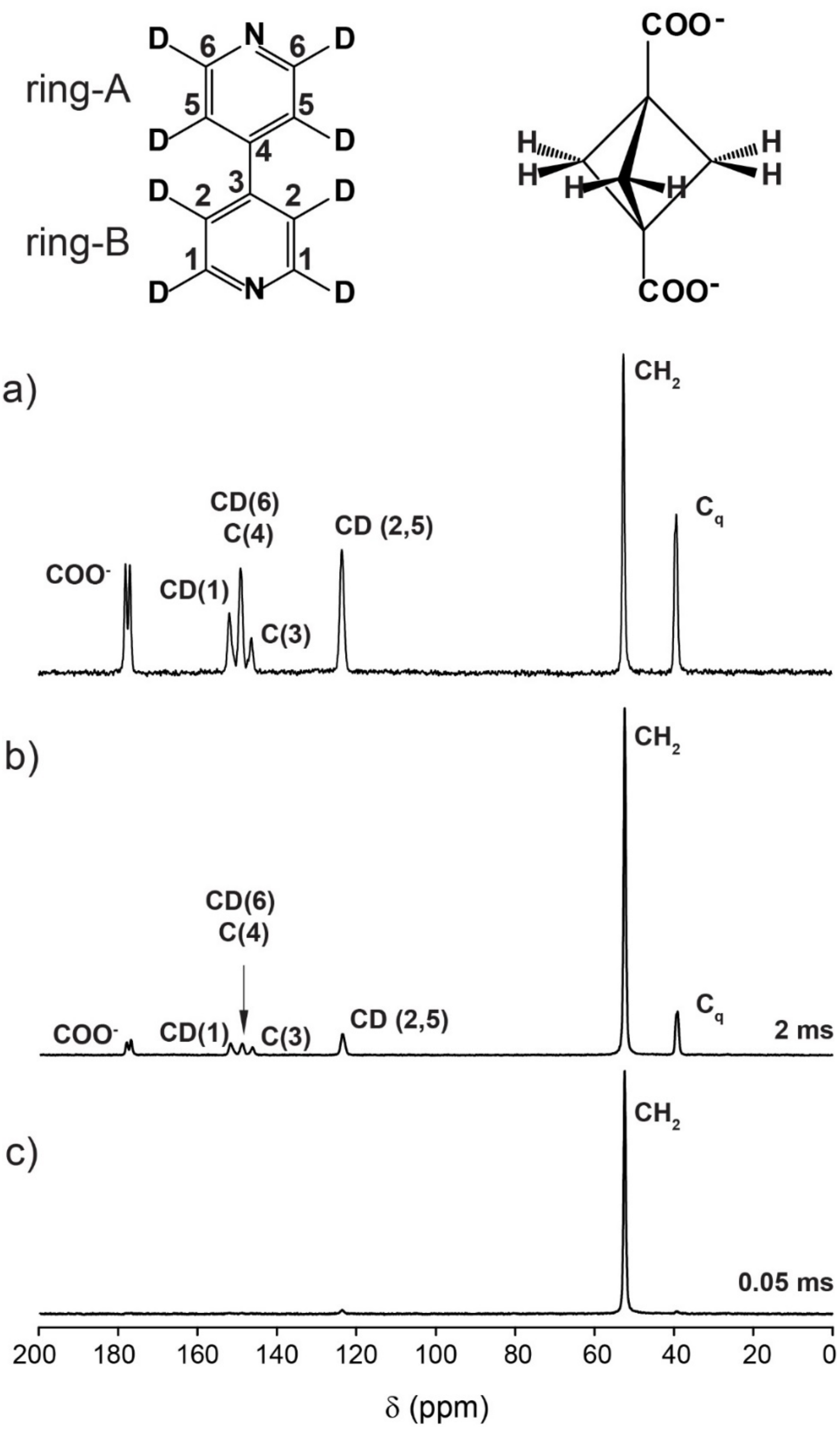

Figure S28. ^{13}C MAS NMR analysis of FTR-P1 performed at 293 K at a spinning speed of 12.5 kHz: a) Quantitative $^{13}C\{^{1}H\}$ SPE spectrum collected with a recycle delay of 60 s; $^{13}C\{^{1}H\}$ CP spectra collected with contact time of 2 ms (b) and 0.05 ms (c).

Table S3. ^{13}C shifts of PDR-3 from the simulation of quantitative ^{13}C{^{1}H} SPE MAS spectrum collected at 293 K at 7.04 T. ^{13}C chemical shifts of FTR-P1 from ^{13}C{^{1}H} CP MAS spectrum collected at 210 K at 7.04 T.

<table>
<tr><th>FTR-P1 β form
(T = 293 K)</th><th>Assignment</th><th>δ (ppm)
^{13}C{^{1}H} SPE MAS</th><th>Amount
%</th></tr>
<tr><td rowspan="7">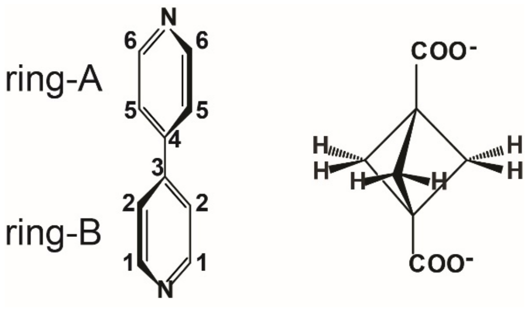
</td><td>C_1</td><td>152.10</td><td rowspan="4">39.8%</td></tr>
<tr><td>C_4, C_6</td><td>149.46</td></tr>
<tr><td>C_3</td><td>147.10</td></tr>
<tr><td>C_2, C_5</td><td>124.18</td></tr>
<tr><td>COO^-</td><td>178.10, 177.05</td><td rowspan="3">60.2%</td></tr>
<tr><td>CH_2</td><td>52.49</td></tr>
<tr><td>Cq</td><td>39.48, 39.01</td></tr>
<tr><th>FTR-P1 α form
(T = 210 K)</th><th>Assignment</th><th colspan="2">δ (ppm)
^{13}C{^{1}H} CP MAS</th></tr>
<tr><td rowspan="7"></td><td>C_1, C_6</td><td colspan="2">151.68</td></tr>
<tr><td>C_4</td><td colspan="2">150.25</td></tr>
<tr><td>C_3</td><td colspan="2">147.56</td></tr>
<tr><td>C_2, C_5</td><td colspan="2">124.29</td></tr>
<tr><td>COO^-</td><td colspan="2">177.89, 176.64</td></tr>
<tr><td>CH_2</td><td colspan="2">52.02</td></tr>
<tr><td>Cq</td><td colspan="2">39.16, 38.60</td></tr>
</table>

Table S4. ^{13}C shifts of FTR-P1d from the simulation of quantitative ^{13}C SPE MAS spectrum collected at 293 K at 7.04 T.

FTR-P1 β form (T = 293 K)	Assignment	δ (ppm)	Amount %
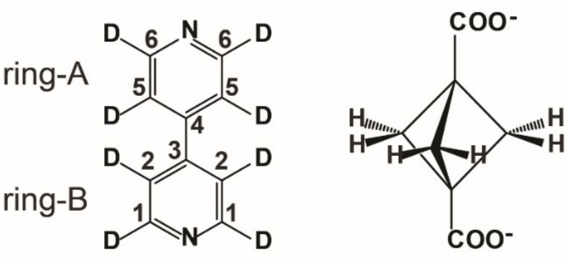	C_1	151.85	39.7%
	C_4, C_6	149.03	
	C_3	146.37	
	C_2, C_5	123.51	
	COO^-	178.10, 177.01	60.3%
	CH_2	52.48	
	Cq	39.57, 39.09	

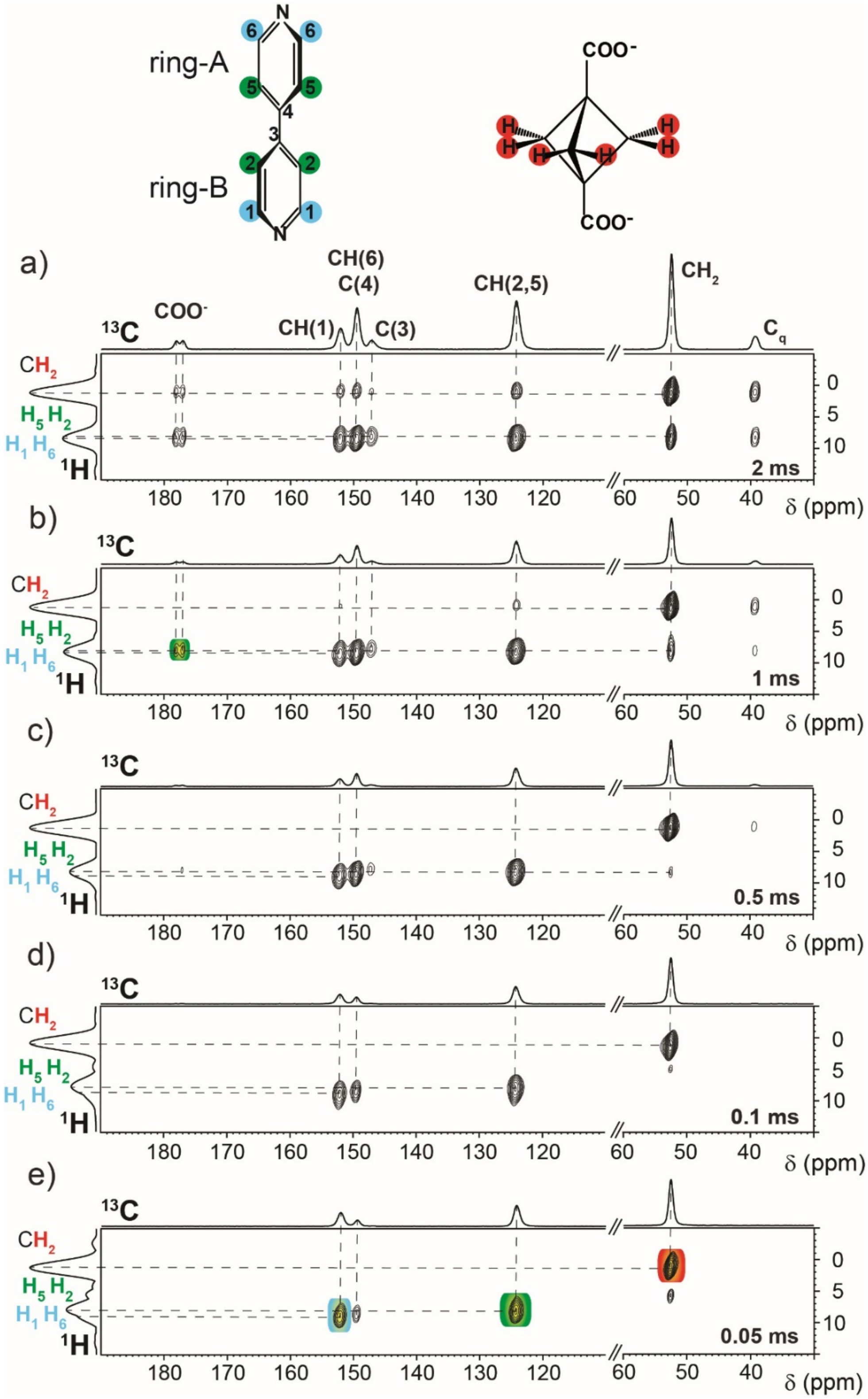

Figure S29. 2D ^{1}H-^{13}C PMLG HETCOR NMR spectra of FTR-P1d collected at 298 K, at a spinning speed of 12.5 kHz and contact times of 2 ms (a), 1 ms (b), 0.5 ms (c), 0.1 ms (d), and 0.05 ms (e).

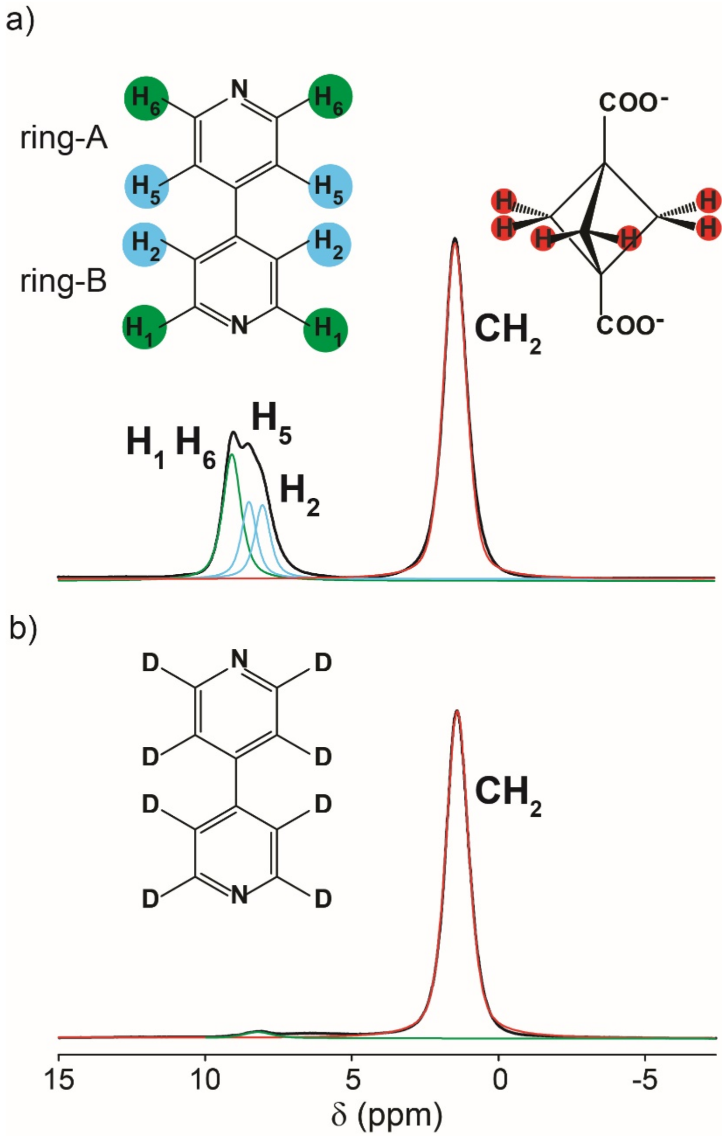

Figure S30. ^{1}H MAS spectra collected at 298 K with a spinning speed of 30 kHz and a recycle delay of 20 s of a) FTR-P1 and b) FTR-P1d.

Table S5. ^{1}H chemical shifts of FTR-P1 and FTR-P1d from the simulation of quantitative ^{1}H MAS spectra collected at 30 kHz, 298 K at 14 T. ^{1}H signals have been assigned by comparison of solid state NMR spectra with ^{1}H solution NMR spectrum of pure 4,4'-bipyridine and 4,4'-bipyridine in metal complexes.[7]

<table>
<tr><th>FTR-P1
(T = 293 K)</th><th>Assignment</th><th>δ (ppm)</th><th>Amount %
(internal ratio)</th></tr>
<tr><td rowspan="4">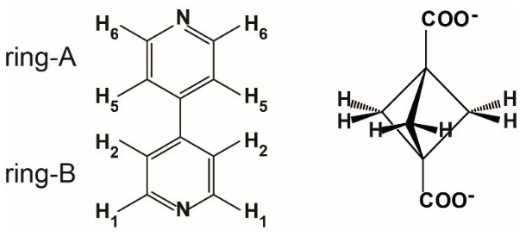
</td><td>H_1 H_6</td><td>9.08</td><td rowspan="3">40.0%
(2:1:1)</td></tr>
<tr><td>H_5</td><td>8.50</td></tr>
<tr><td>H_2</td><td>8.04</td></tr>
<tr><td>CH_2</td><td>1.49</td><td>60.0%
(6)</td></tr>
<tr><td rowspan="2">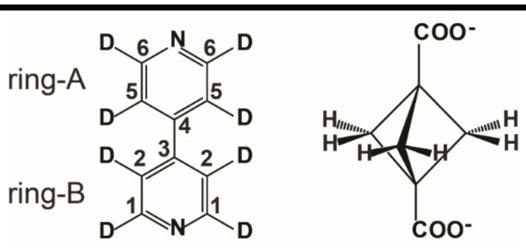
</td><td>H_{arom}</td><td>8.20</td><td>2.4%</td></tr>
<tr><td>CH_2</td><td>1.4</td><td>97.6%</td></tr>
</table>

^{2}H NMR RESULTS OF FTR-P1d

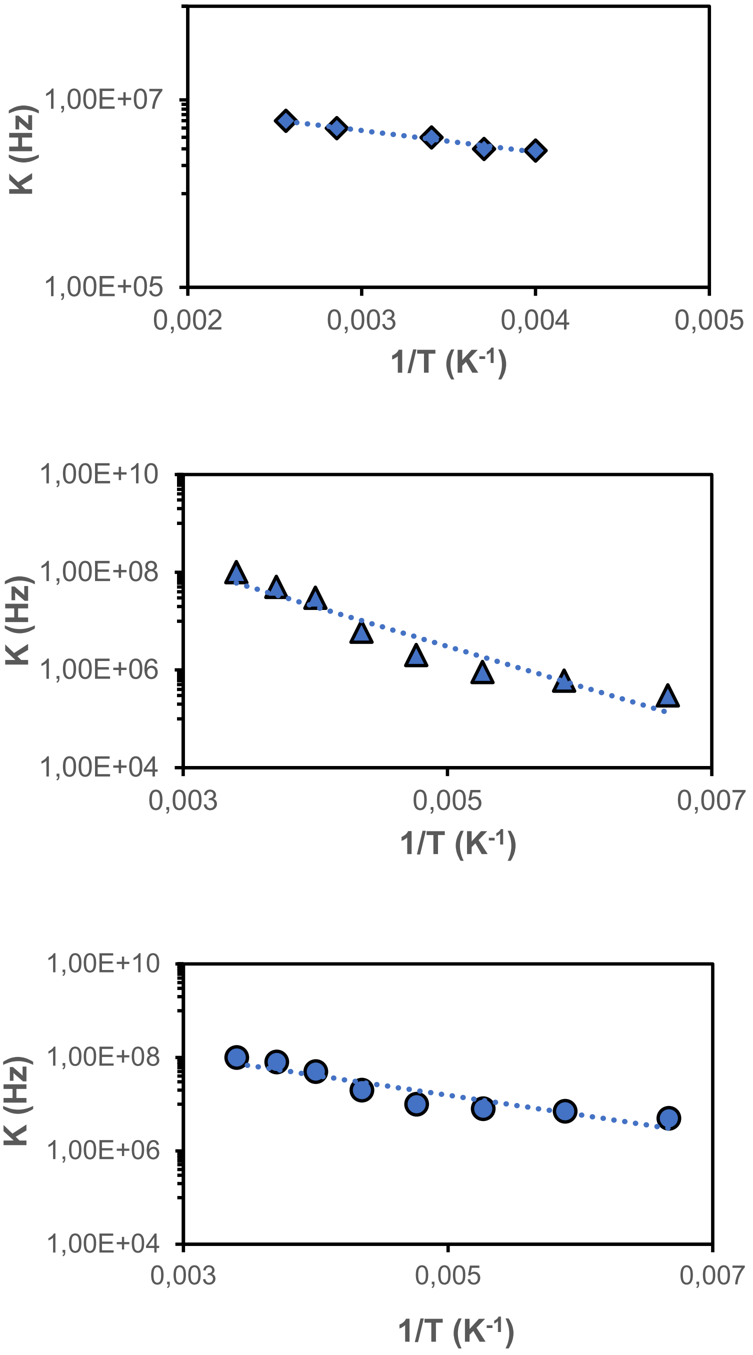

Figure S31. Arrhenius plots of reorentation frequencies versus the inverse of temperature from ^{2}H NMR spectra of FTR-P1d. E_a=1.2 kcal/mol for 4-site flip reorentation ±39.5°, ±140.5° of ring A in the β-phase (blue diamonds), E_a=3.3 kcal/mol for 2-site 180°C flip reorientation for ring B in the β-phase (blue triangles), E_a=2.0 kcal/mol 2-site 180° flip reorientation of ring A in the α-phase (blue circles). The error bars on temperature are smaller than the symbols.

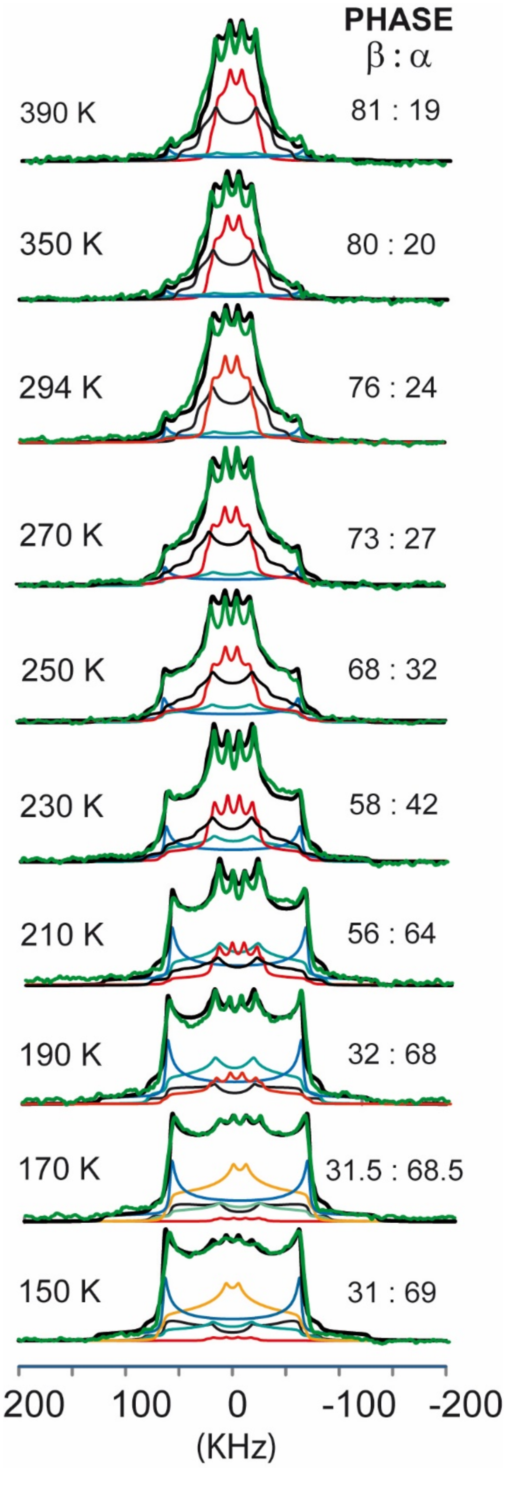

Figure S32. ^{2}H solid-echo NMR spectra of bipyridine rings in FTR-P1d at distinct temperatures and the simulated spectra considering the following components: 4-site ±39.5° ±140.5° reorientation plus ±39.5° jumps for ring A in the β-phase (red line), 2-site 180° reorientation plus ± 28° jumps for ring B in the β-phase (black line), 2 site 180° reorientation for ring A in the α-phase (green line), static pattern for ring B in the α-phase (blue line) and 2-site jumps ±39.5° (yellow line) for ring A in the both phases.

Table S6. Fraction of α- and β-phases as function of temperature of FTR-P1d from ^2H NMR analysis and the corresponding estimated errors.

Temperature (K)	α-phase	Estimated error	β-phase	Estimated error
390	19	3	81	3
350	20	3.5	80	3.5
294	24	3.7	76	3.7
270	27	5	73	5
250	32	4.2	68	4.2
230	42	3.6	58	3.6
210	64	3.2	36	3.2
190	68	2.8	32	2.8
170	68,5	3.4	31,5	3.4
150	69	2.5	31	2.5
390	19	3	81	3

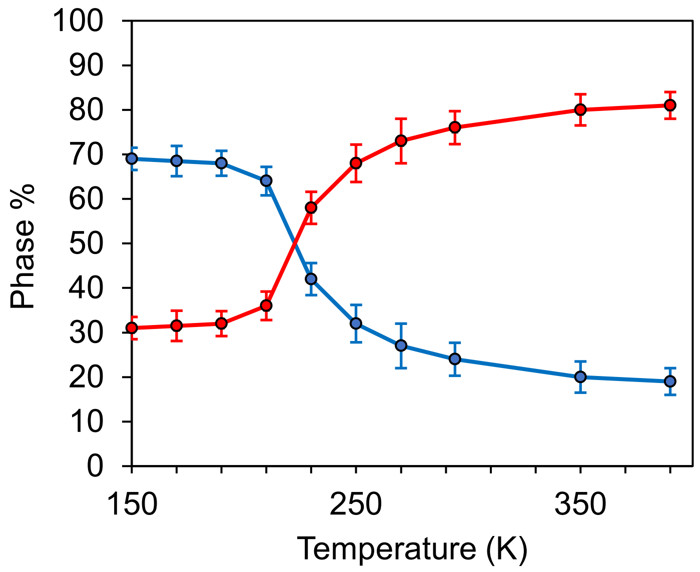

Figure S33. Ratio of α- (blue line) and β-phase (red line) of FTR-P1d from the deconvoluted ^2H NMR spectra.

Table S7. Reorentational frequencies (k) of bipyridine rings from ^{2}H solid-echo NMR lineshape analysis of FTR-P1d.

Temperature	β-phase	β- phase	α- phase	α- phase
	ring A	ring B	ring A	ring B
	4-site ±39.5°/±140.5° + jumps ±39.5°	2-site 180° + jumps ±28°	2-site 180°	static
	(Hz)	(Hz)	(Hz)	(Hz)
390.0 ± 0.2				
350.0 ± 0.2	6.00E+06			
294.0 ± 0.2	5.00E+06			
270.0 ± 0.2	4.00E+06	1.00E+08	1.00E+08	< 1.00E+04
250.0 ± 0.2	3.00E+06	5.00E+07	8.00E+07	< 1.00E+04
230 ± 0.2	2.50E+06	3.00E+07	5.00E+07	< 1.00E+04
210.0 ± 0.2		6.00E+06	2.40E+07	< 1.00E+04
190.0 ± 0.2		2.30E+06	2.00E+07	< 1.00E+04
170.0 ± 0.2		9.40E+05	1.00E+07	< 1.00E+04
150.0 ± 0.2		6.00E+05	7.00E+06	< 1.00E+04
390.0 ± 0.2		2.00E+05	4.00E+06	< 1.00E+04

Table S8. Activation energies of bipy rings for FTR-P1d.

	β-phase	β - phase	α- phase	α- phase
	ring A	ring B	ring A	ring B
	4-site ±39.5°/±140.5° + jumps ±39.5°	2-site 180° + jumps ±28°	2-site 180°	
**E_a (kcal//mol)**	1.2	3.9	2.0	-
**K_0 (Hz)**	2.9E+07	5.1E+10	2.8E+09	-
**$\Delta H^{\neq}$ (kcal/mol)**	0.58	3.5	1.6	-
**$\Delta S^{\neq}$ (cal/mol K)**	-26	-11	-17	-

Jumps ±28°: Ea=3.3 kcal/mol and K_0=5.6E+10 Hz.

Table S9. Fraction (%) of distinct motional mechanisms at different temperatures for bipyridine rings A and B in FTR-P1d as obtained from ^{2}H solid-echo NMR spectra.

		β-phase		α-phase	
**Temp.**	**Mechanism**	**ring A**	**ring B**	**ring A**	**ring B**
390.0 ± 0.2 K	4-site ±39.5°/±140.5°	40%			
	180°/ ±28° jumps		40%		
	jump ±39.5°				
	2-site 180°			10%	
	static				10%
350.0 ± 0.2 K	4-site ±39.5°/±140.5°	41%			
	jumps 180°/ ±28°		41%		
	jump ±39.5°				
	2-site 180°			9%	
	static				9%
294.0 ± 0.2 K	4-site ±39.5°/±140.5°	33%			
	jumps 180°/ ±28°		38%		
	jump ±39.5°	5%			
	2-site 180°			12%	
	static				12%
270.0 ± 0.2 K	4-site ±39.5°/±140.5°	23.5%			
	2-site 180°/ ±28°		20.0%		
	jump ±39.5°	9.5%			
	2-site 180°		20.0%	13.5%	
	static				13.5%
250.0 ± 0.2 K	4-site ±39.5°/±140.5°	18%			
	2-site 180°/ ±28°		23%		
	jump ±39.5°	16%			
	2-site 180°		11%	16%	
	static				16%
230.0 ± 0.2 K	4-site ±39.5°/±140.5°	15%			
	2-site 180°/ ±28°		7%		
	jump ±39.5°	14%			
	2-site 180°		22%	21%	
	static				21%
210.0 ± 0.2 K	4-site ±39.5°/±140.5°	9%			
	2-site 180°/ ±28°		2%		

	jump ±39.5°	9%			
	2-site 180°		16%	32%	
	static				32%
	4-site ±39.5°/±140.5°	3%			
	2-site 180°/ ±28°				
190.0 ± 0.2 K	jump ±39.5°	13%			
	2-site 180°		16%	34%	
	static				34%
	4-site ±39.5°/±140.5°	1%			
	jump ±28°				
170.0 ± 0.2 K	jump ±39.5°	15%		20%	
	2-site 180°		16%	14%	
	static				34%
	4-site ±39.5°/±140.5°	1%			
	2-site 180°/ ±28°				
150.0 ± 0.2 K	jump ±39.5°	15%		20%	
	2-site 180°		16%	14%	
	static				34%

^{1}H T$_1$ NMR RESULTS OF FTR-P1d

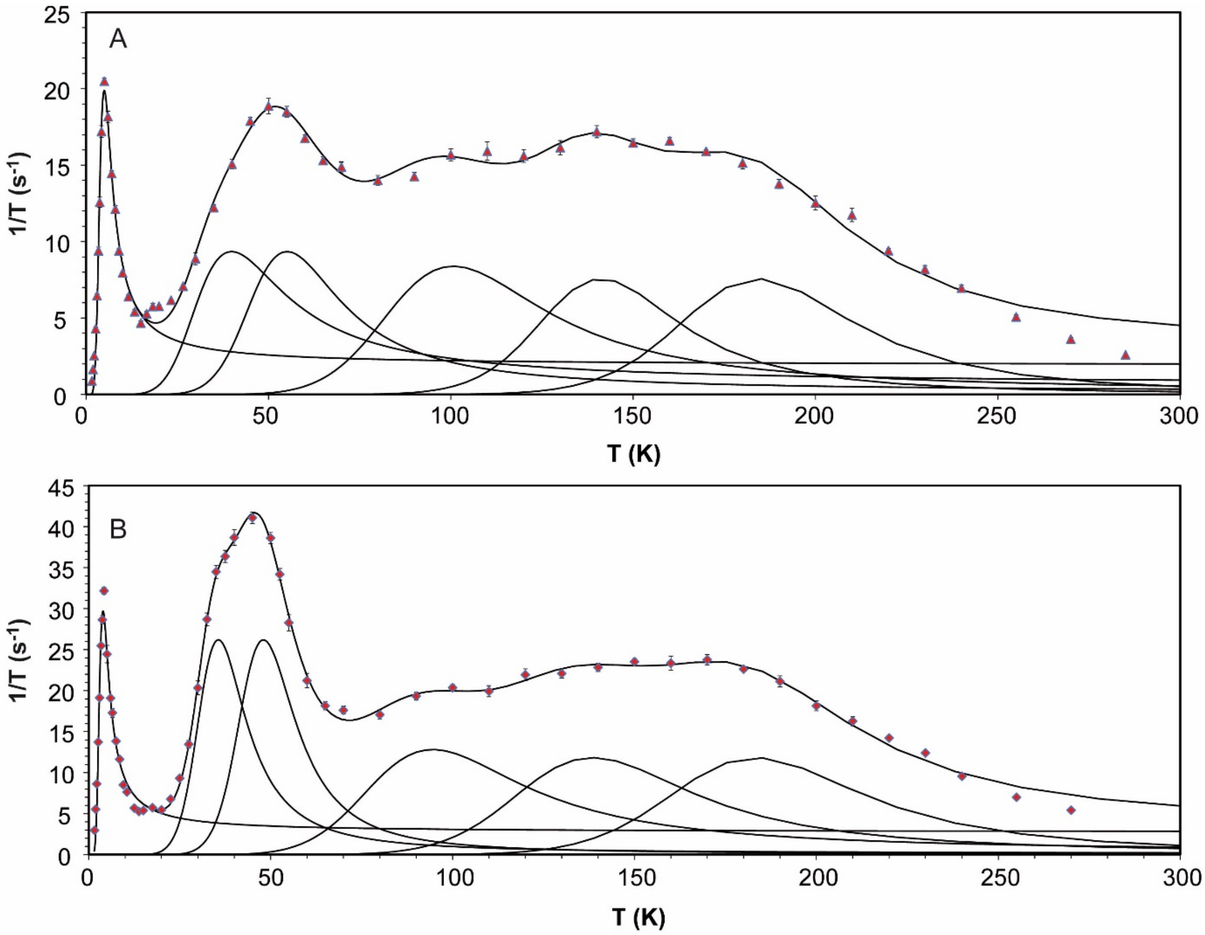

Figure S34. ^{1}H relaxation rates as function of temperature of FTR-P1d at two distinct magnetic 45.86 (A) and 16.56 MHz (B). The error bars on temperature are smaller than the symbols.

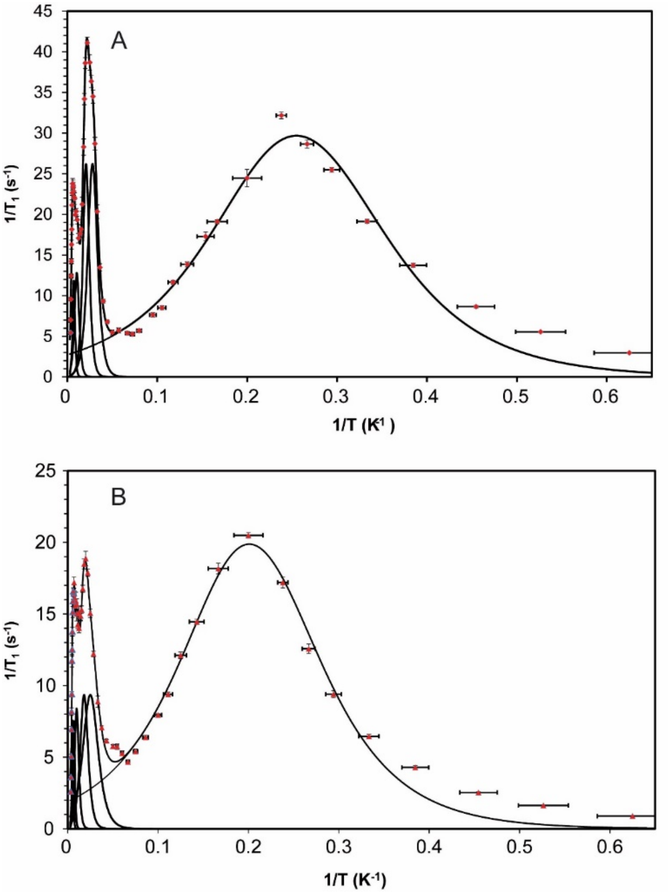

Figure S35. [1]H relaxation rates as function of reciprocal of temperature of FTR-P1d at two distinct magnetic 16.56 (A) and 45.86 MHz (B). The error bars on temperature are smaller than the symbols.

Table S10. Activation energy and C constant for BCP rotators from ^{1}H T_1 relaxation times at 1.08 T in FTR-P1d sample.

**Component**	**1**	**2**	**3**	**4**	**5**	**6**
**C (10^9 s^{-2})**	2.17	1.92	1.92	0.94	0.87	0.87
**E_A (kcal/mol)**	0.024	0.455	0.688	0.940	1.816	2.947

Table S11. Activation energy and C constant for BCP rotators from ^{1}H T_1 relaxation times at 0.39 T in FTR-P1d sample.

**Component**	**1**	**2**	**3**	**4**	**5**	**6**
**C (10^9 s^{-2})**	4.03	1.89	1.89	1.70	1.53	1.53
**E_A (kcal/mol)**	0.031	0.547	0.281	1.050	2.419	3.296

POTENTIAL ENERGY SCANS FOR THE BCP CONFORMATIONS AND ROTORS IN FTR-P1

DFT scans using reduced model containing only BCP rotors. Potential energy scans were performed using the GAUSSIAN16 software available through the CINECA high performance computing centre.[15] We built a model considering an ensemble of eight vicinal rotators as arranged within the crystal structure (Figure S27). Since these arrangements are kept in place by the metal coordination and other structural factors, the internal coordinates of the moieties, and their relative position were frozen. The 2D potential energy rigid scans for the rotors were performed using M062X/6-311+G(d) level of theory[16] with Counterpoise correction. For the 2D rigid scan only rotors **R1-R2** and **R1-R3** were considered (0° – 120° with a scan step of 5° for each rotor) and their interactions with the vicinal rotors calculated separately. The corrections for the R1-R2 system comprised two side-ways and two downwards corrections while the **R1-R3** system only needed the side-ways correction (Figure S28). The corrections were calculated by scanning one rotor (0° – 120° with a scan step of 5°) while the vicinal rotors, sideways or downwards, could optimize their rotor rotational torsion angle. Since the rotors has a three-fold symmetry, a 120° scan would be sufficient in all cases. The side-ways and downwards interaction potential only had to be calculated one and then added to the 2D scan results in the proper manner. For example, if R1 and R2 has the angles 30° and 5° for the 2D scan, the energy at this position should be corrected by the sideways and downwards potential energies at both 30° and 5°:

$$\boldsymbol{rel.E(R1-R2)_{30°,5°}} = \Delta E_{R1R2}(2D\ scan) + \Delta E_{R1}(side, 30°) + \Delta E_{R2}(side, 5°) + \Delta E_{R1}(down, 30°) + \Delta E_{R2}(down, 5°)$$

$$\boldsymbol{rel.E(R1-R3)_{30°,5°}} = \Delta E_{R1R3}(2D\ scan) + \Delta E_{R1}(side, 30°) + \Delta E_{R3}(side, 5°) + \Delta E_{R1}(side, 30°) + \Delta E_{R3}(side, 5°)$$

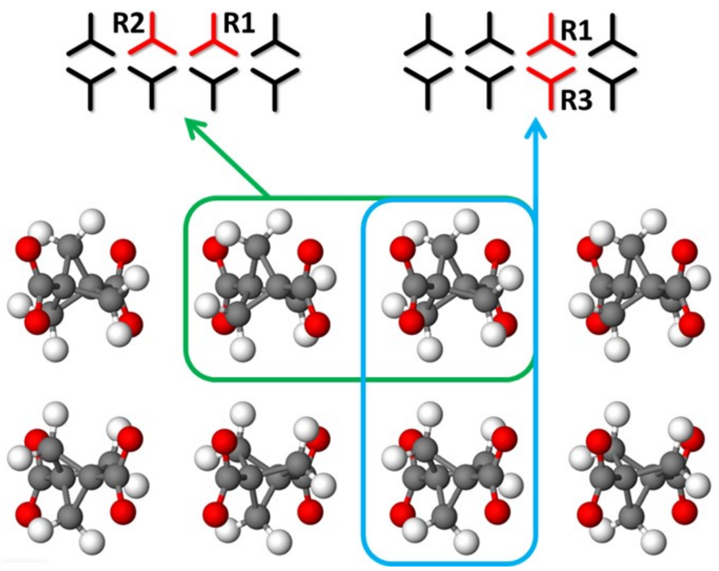

Figure S36. The DFT model considered the **R1-R2** (green box) and **R1-R3** (blue box). Due to the scale of the model and computational expense, the calculation was split into parts with the 2D scans calculated using only the R**1-R2** and **R1-R3** moieties. The interactions of these moieties with molecules below them and to their sides were calculated separately and added appropriately to the 2D scan results.

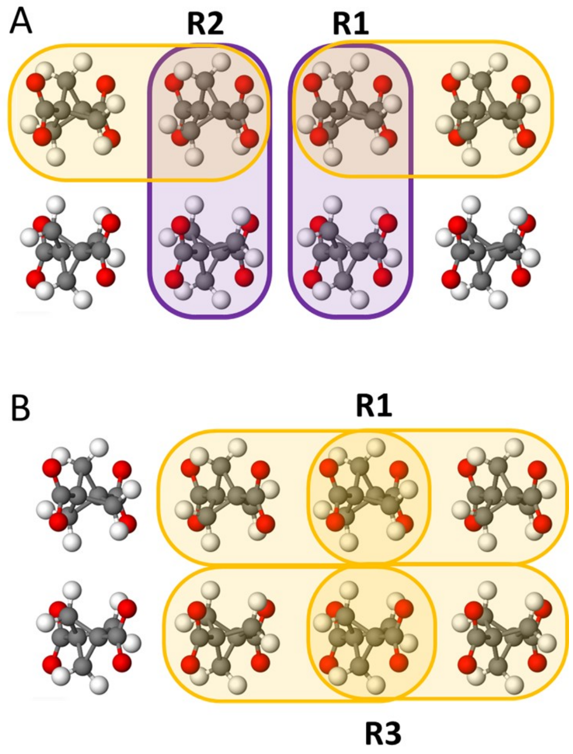

Figure S37. The DFT model with the corrective interactions indicated. A) For the **R1-R2** side-by-side2D scan, interactions with FTR on their side (yellow box) and below (purple box) had to be calculated and accounted for. B) For the **R1-R3** up-and-down 2D scan, only interactions with FTR on their side (yellow box) had to be calculated and accounted for.

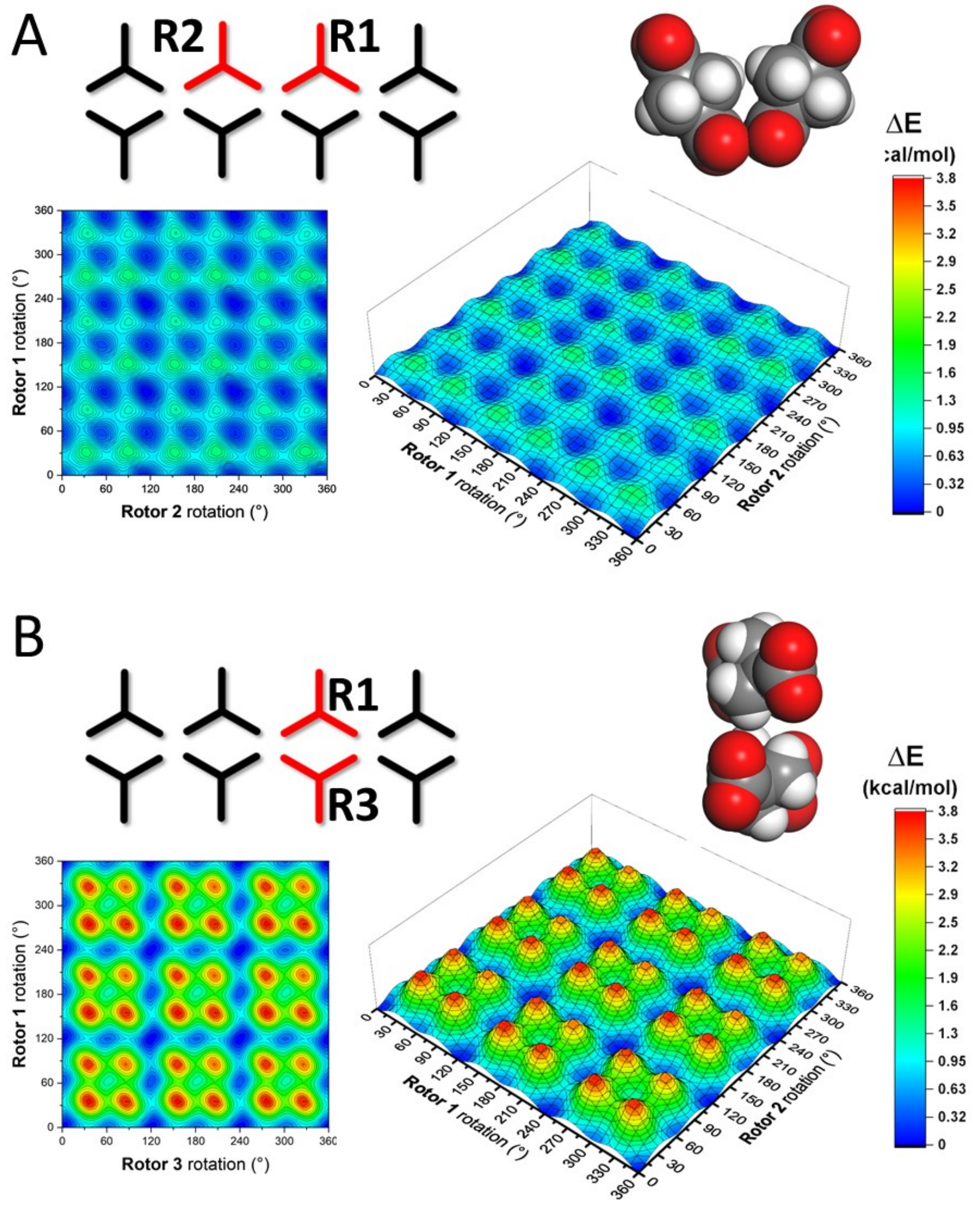

Figure S38. The results of the DFT 2D scans. A) Scan for rotors **R1-R2** and B) for **R1-R3**. The diagrammatic representation of the model is shown and to the right a space-filling representation of the rotor pairs in their crystallographic positions. The potential energy maps are shown as both a 2D projection map (left) and a 2D surface map (right). All the maps are plotted to the same ΔE scale of 0 – 3.8 kcal/mol for visual comparison of the energy landscapes.

CO_2 ADSORPTION AND ADSORPTION-COUPLED CALORIMETRY IN FTR-P1

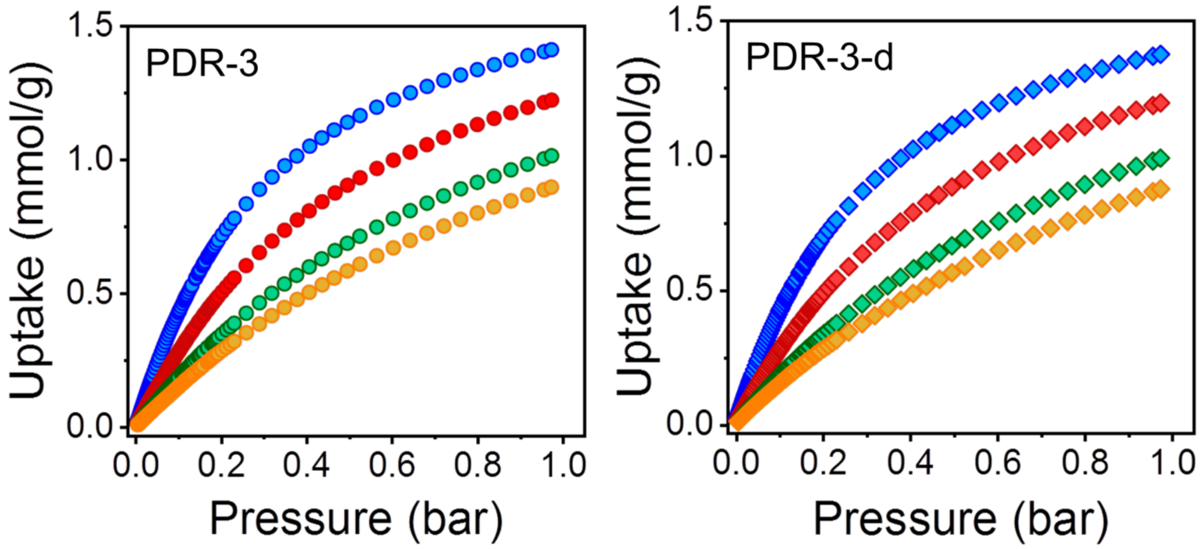

Figure S39. Left: variable temperature CO_2 adsorption isotherms collected up to 1 bar on ASAP 2020 for FTR-P1 (circles). Right: variable temperature CO_2 adsorption isotherms collected up to 1 bar on ASAP 2020 for FTR-P1d (diamonds). Blue, T = 273 K. Red, T: 283 K. Green, T: 293 K. Orange, T: 298 K.

Calculation of isosteric heat of adsorption: fitting with Langmuir adsorption isotherm. CO_2 adsorption data were fitted according to Langmuir model in order to calculate the isosteric heat of adsorption of FTR-P1 and FTR-P1d.

$$Q = \frac{Q_{sat} \cdot K \cdot p}{1 + K \cdot p}$$

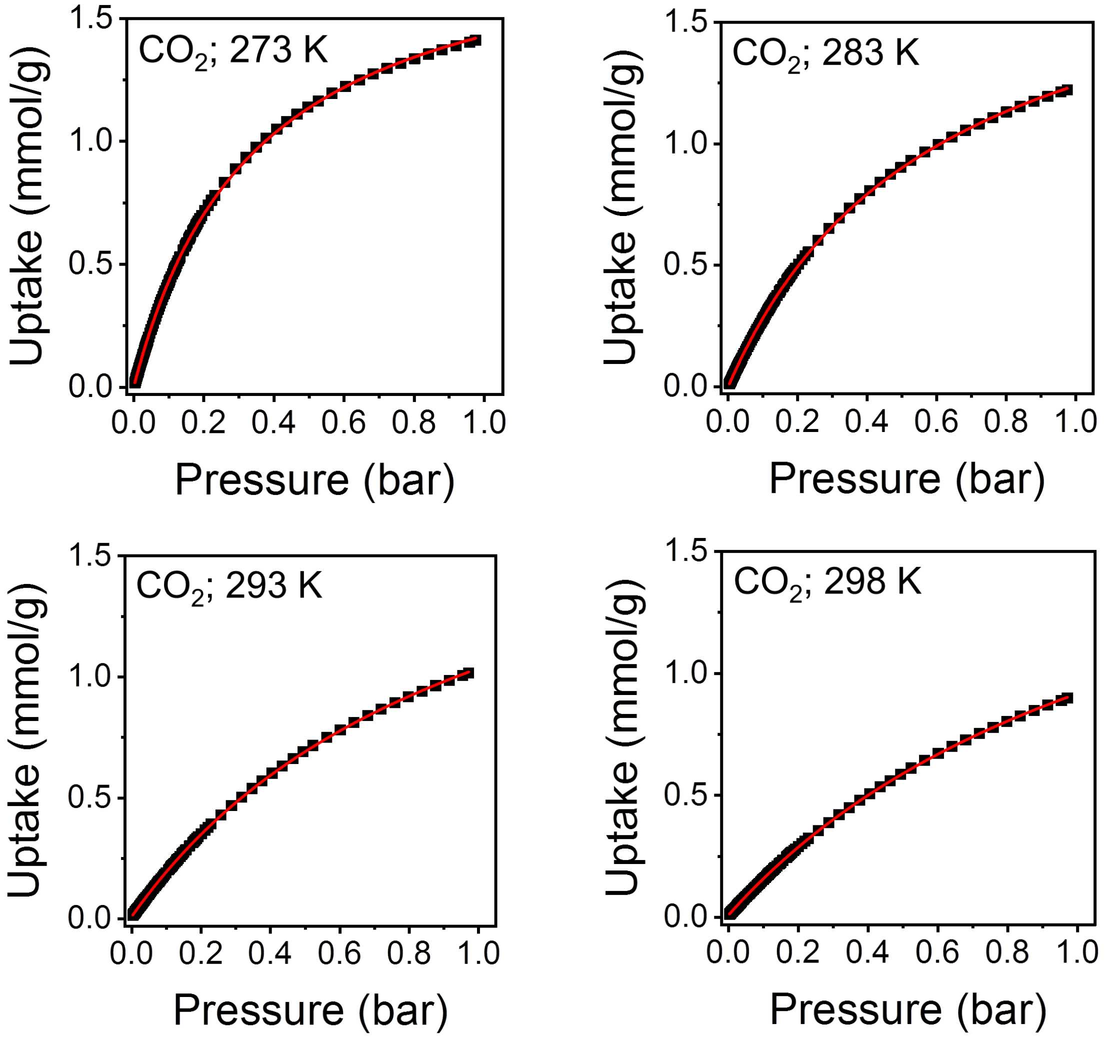

PDR-3	Q_{sat} (mmol/g)	K (1/bar)	R^2
273 K	1.924 ± 0.005	2.88 ± 0.013	0.99977
283 K	1.990 ± 0.005	1.655 ± 0.007	0.99986
293 K	2.084 ± 0.007	0.978 ± 0.005	0.99991
298 K	2.101 ± 0.008	0.767 ± 0.004	0.99992

Figure S40. Fitting of experimental adsorption data of FTR-P1 with Langmuir adsorption isotherm and calculated parameters at different temperatures.

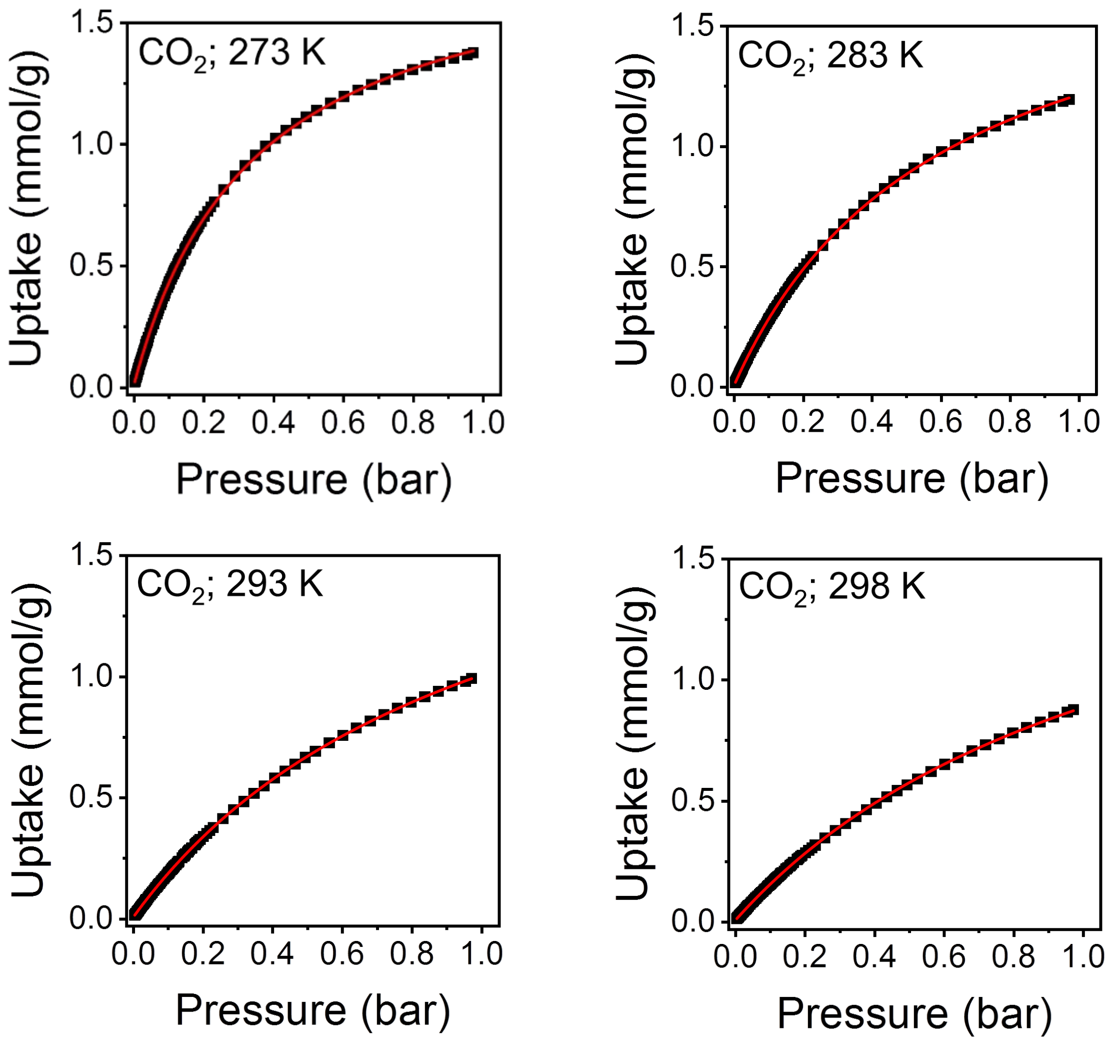

PDR-3-d	Q_{sat} (mmol/g)	K (1/bar)	R^2
273 K	1.870 ± 0.003	2.91 ± 0.01	0.99988
283 K	1.946 ± 0.004	1.651 ± 0.005	0.99994
293 K	2.025 ± 0.002	0.980 ± 0.002	0.99999
298 K	1.946 ± 0.006	0.828 ± 0.004	0.99994

Figure S41. Fitting of experimental adsorption data of FTR-P1d with Langmuir adsorption isotherm and calculated parameters at different temperatures.

Fitting with Langmuir-Freundlich adsorption isotherm. CO_2 adsorption data were fitted according to Langmuir-Freundlich model in order to calculate the isosteric heat of adsorption of FTR-P1 and FTR-P1d.

$$Q = \frac{Q_{sat} \cdot K \cdot p^n}{1 + K \cdot p^n}$$

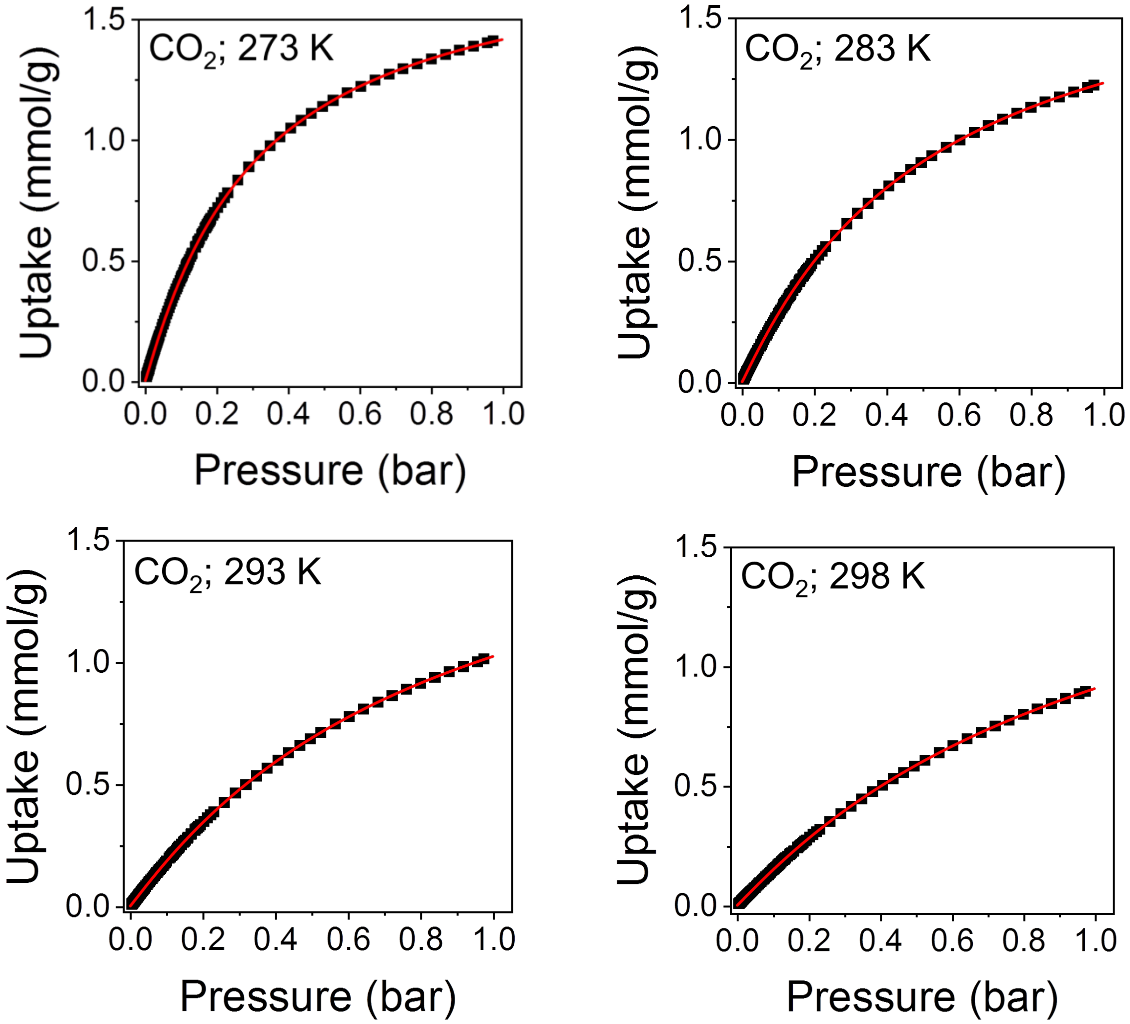

PDR-3	Q_{sat} (mmol/g)	K (1/bar)	n	R^2
273 K	1.823 ± 0.003	3.47 ± 0.017	1.054 ± 0.002	0.99998
283 K	1.856 ± 0.004	1.97 ± 0.01	1.046 ± 0.001	0.99999
293 K	1.900 ± 0.003	1.170 ± 0.0044	1.039 ± 0.001	0.99999
298 K	1.901 ± 0.007	0.912 ± 0.006	1.034 ± 0.002	0.99999

Figure S42. Fitting of experimental adsorption data of FTR-P1 with Langmuir-Freundlich adsorption isotherm and calculated parameters at different temperatures.

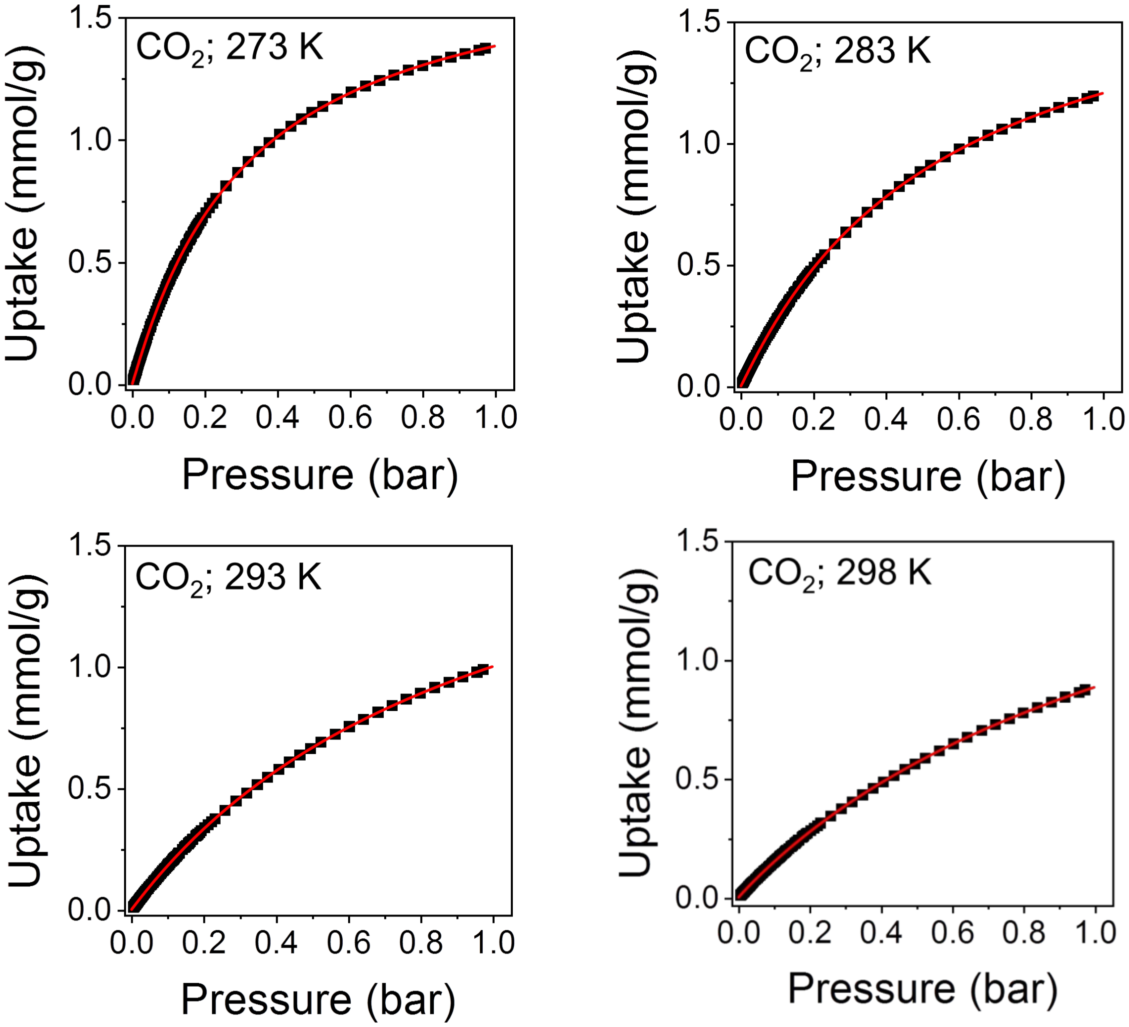

PDR-3-d	Q_{sat} (mmol/g)	*K* (1/bar)	*n*	R^2
273 K	1.800 ± 0.003	3.33 ± 0.015	1.039 ± 0.001	0.99999
283 K	1.856 ± 0.003	1.855 ± 0.007	1.030 ± 0.001	0.99999
293 K	2.000 ± 0.007	1.002 ± 0.006	1.005 ± 0.001	0.99999
298 K	2.10 ± 0.016	0.727 ± 0.009	0.976 ± 0.002	0.99997

Figure S43. Fitting of experimental adsorption data of FTR-P1d with Langmuir-Freundlich adsorption isotherm and calculated parameters at different temperatures.

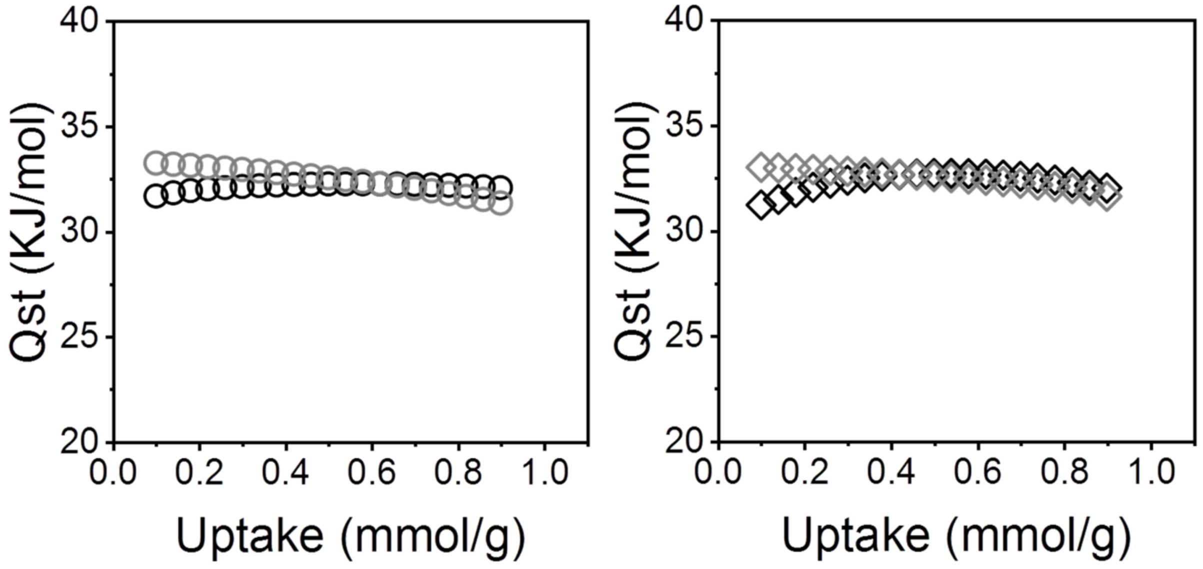

Figure S44. CO_2 isosteric heat of adsorption of FTR-P1 (Left) and FTP-P1d (right) calculated according to Langmuir (Light grey) and Langmuir-Freundlich (black) models.

Determination of CO_2 enthalpy of sorption by direct sorption-coupled microcalorimetry. Sorption-coupled microcalorimetry allowed direct measurement of the enthalpy variation related to CO_2 adsorption. The calorimetry data were recorded on a Setaram μDSC7 Evo instrument equipped with a high pressure sample holder. CO_2 dosing and adsorption isotherms collections were performed with a Micromeritics ASAP 2050 adsorber coupled to the μDSC module. The set-up allowed simultaneous determination of CO_2 adsorption isotherms and of heat exchanged during the adsorption process at each adsorption step.[16] Sorption-coupled microcalorimetry measurements were performed twice at 293 K and the two different runs were averaged to reduce experimental errors.

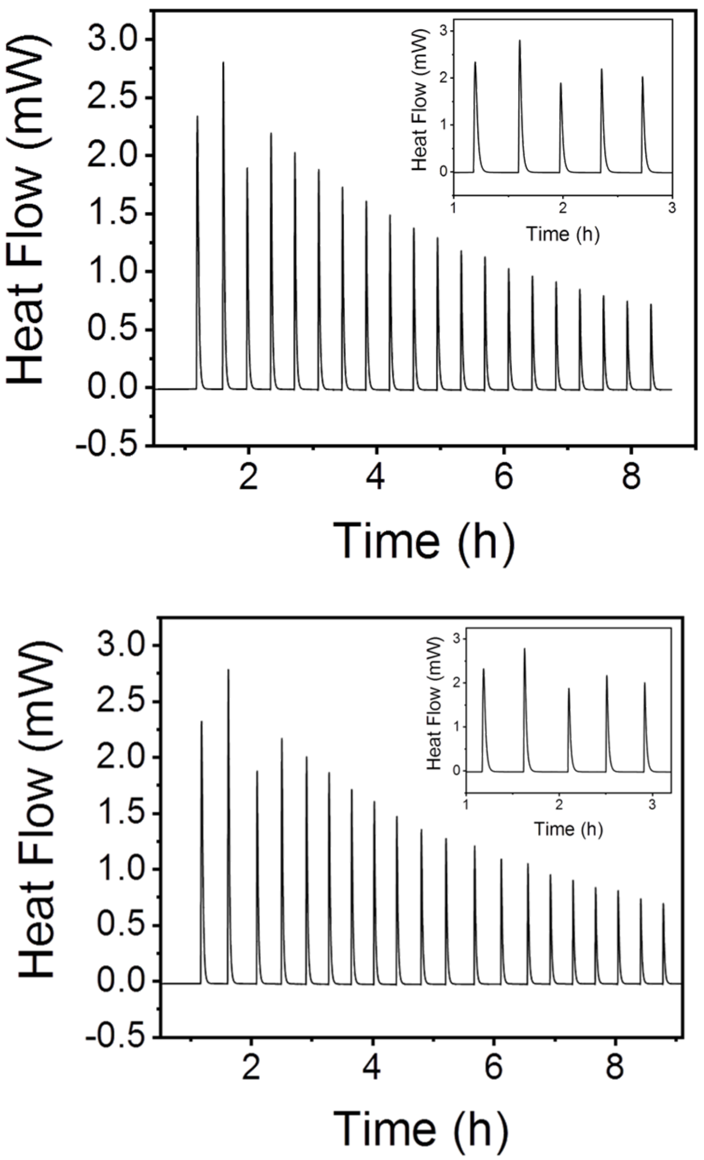

Figure S45. Heat flow measured of the adsorption of CO_2 for FTR-P1 using the ASAP 2050 and μDSC7 coupled system. The top and bottom are the duplicate experiments and the insets shows an expanded timeline. Each peak represents a gas dose and therefor an adsorption point.

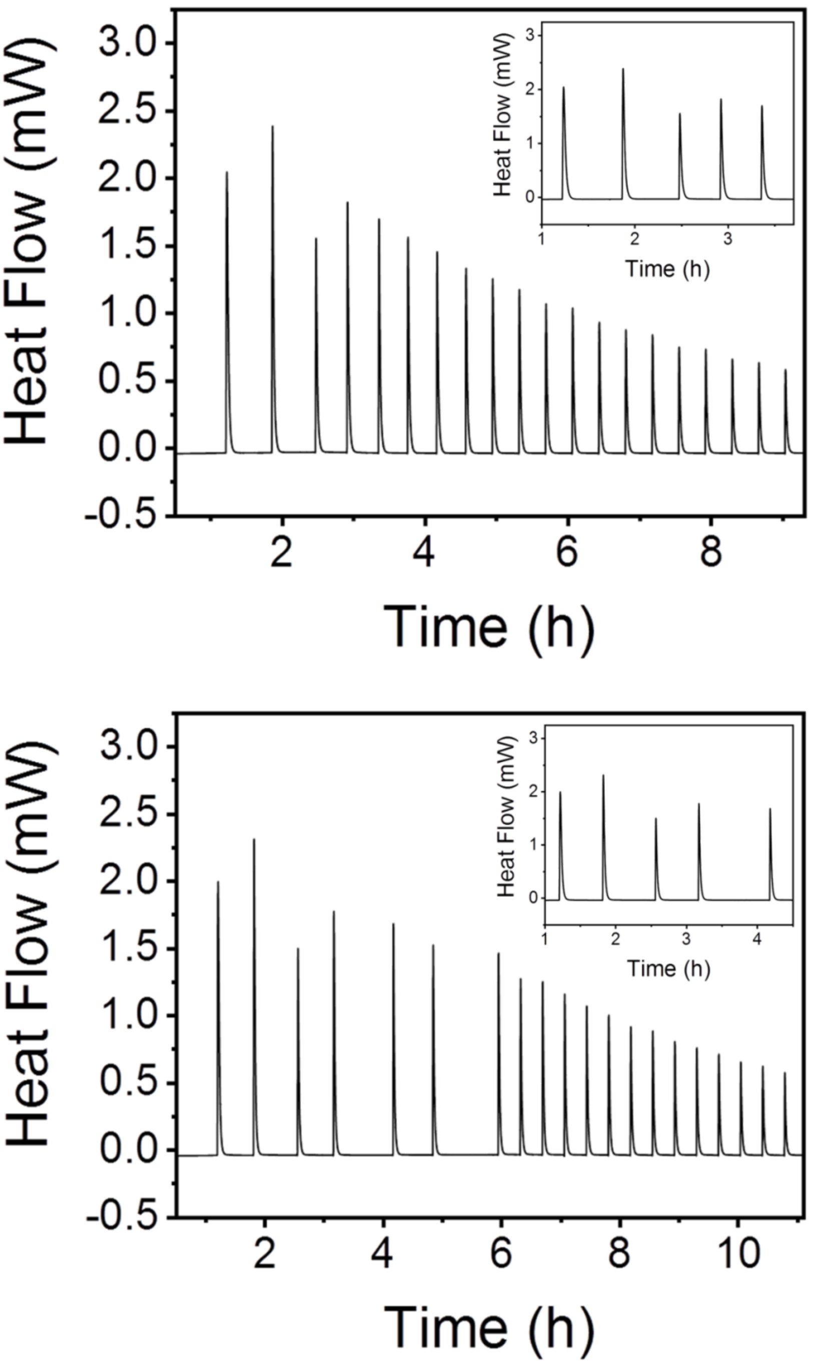

Figure S46. Heat flow measured of the adsorption of CO_2 for FTR-P1d using the ASAP 2050 and μDSC7 coupled system. The top and bottom are the duplicate experiments and the insets shows an expanded timeline. Each peak represents a gas dose and therefor an adsorption point.The heat flows measured with calorimeter were integrated over time to calculate the integral heat associated to each sorption event.

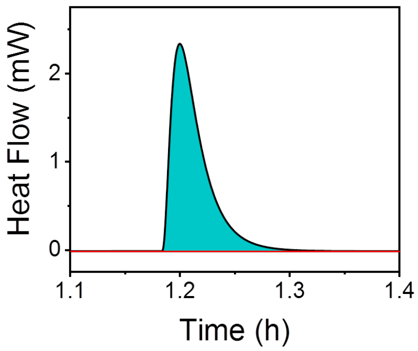

Figure S47. Heat flow integration of one peak of the calorimetry trace. The integral heat corresponded to the blue-shaded area.

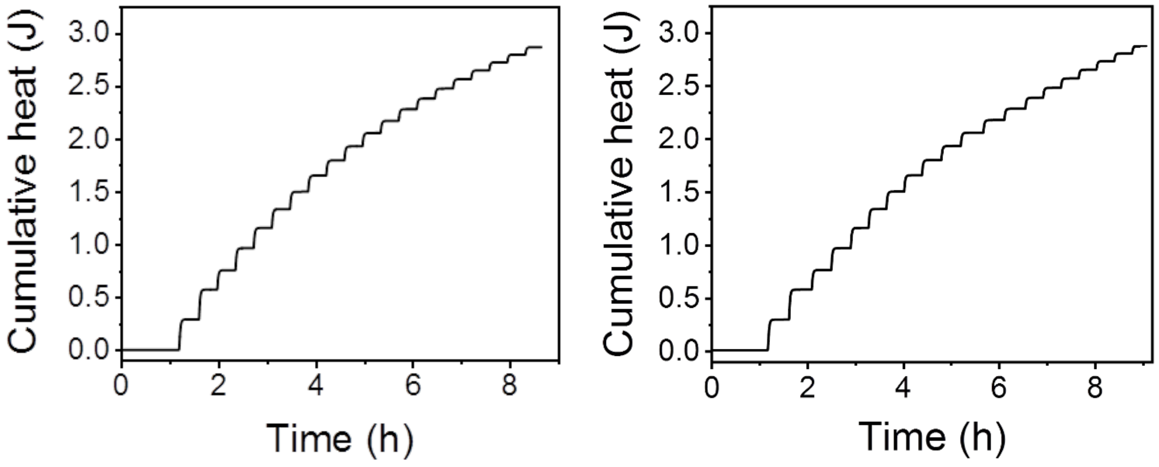

Figure S48. Integral heat released during the adsorption of CO_2 for FTR-P1. First (left) and second (right) runs are reported.

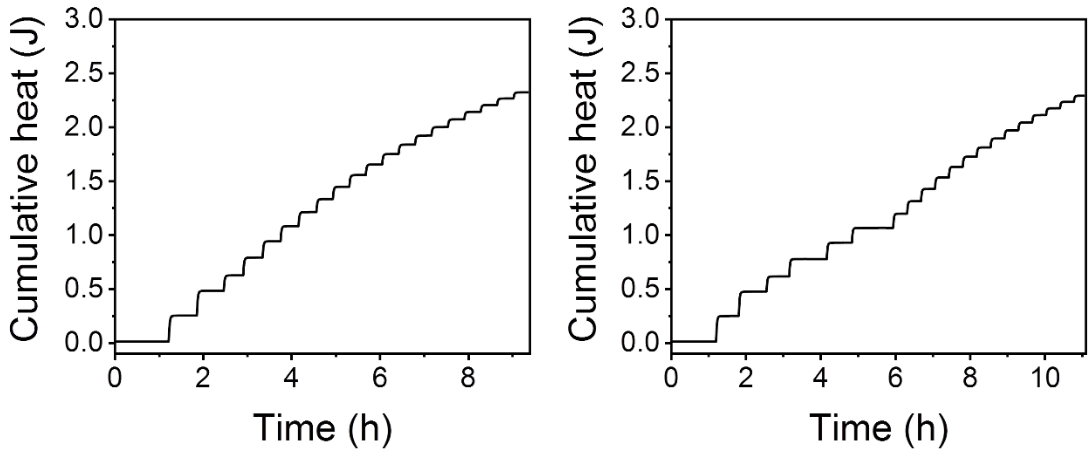

Figure S49. Integral heat released during the adsorption of CO_2 for FTR-P1d. First (left) and second (right) runs are reported.

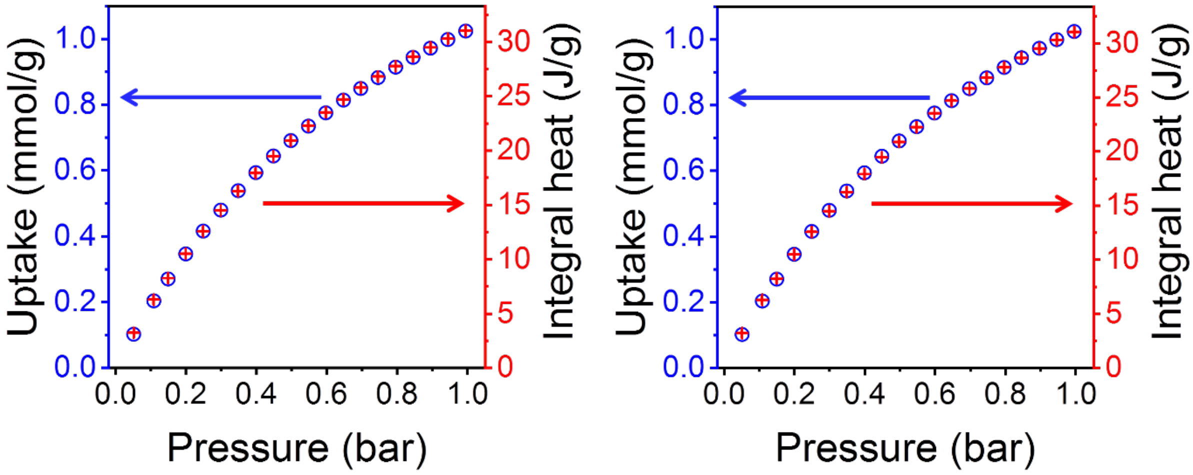

Figure S50. Uptake (mmol/g) and normalized integral heat released during the adsoprtion process for the first run (left) and second run (right) for FTR-P1.

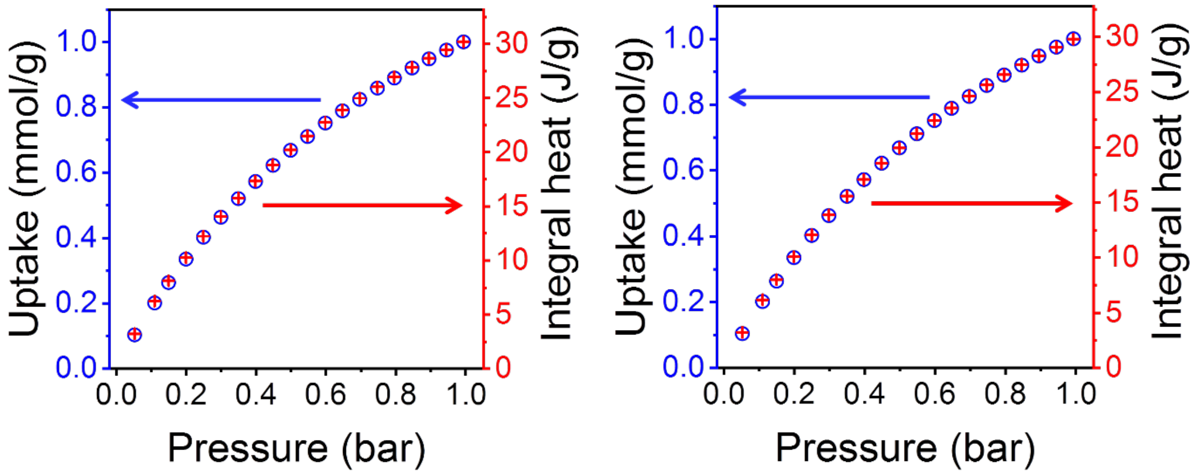

Figure S51. Uptake (mmol/g) and normalized integral heat released during the adsoprtion process for the first run (left) and second run (right) for FTR-P1d.

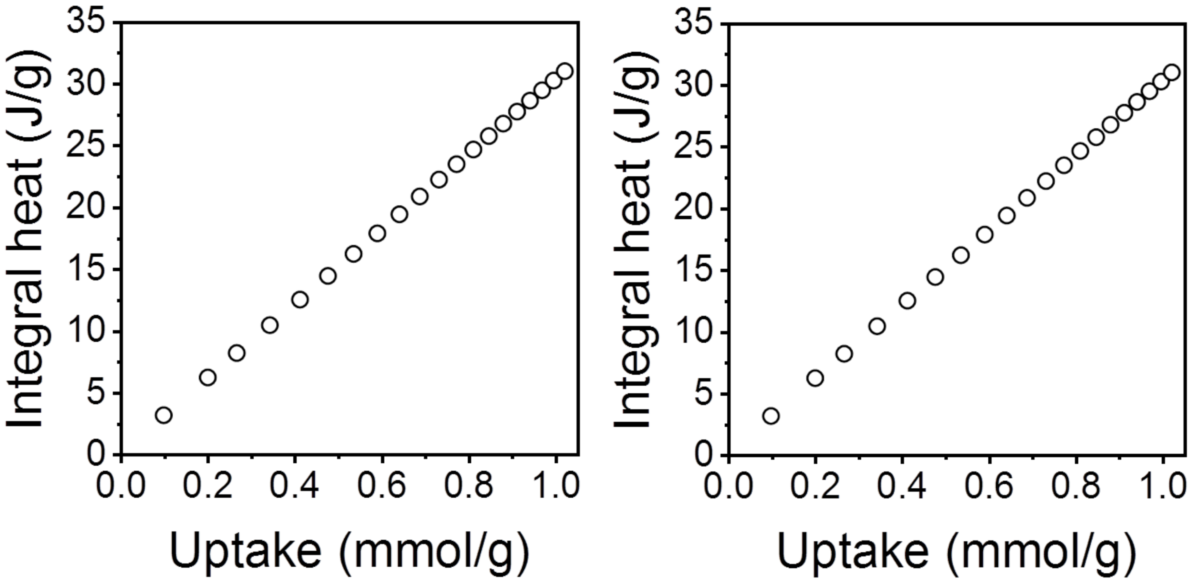

Figure S52. Normalized integral heat (J/g) versus uptake (mmol/g) for the first run (left) and second run (right) for FTR-P1.

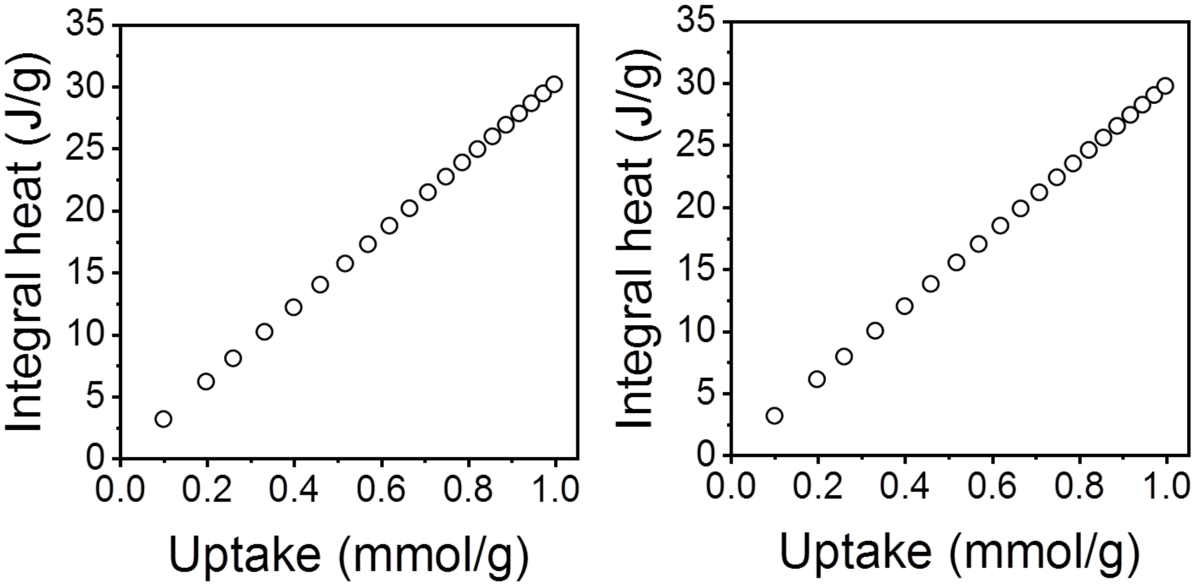

Figure S53. Normalized integral heat (J/g) versus uptake (mmol/g) for the first run (left) and second run (right) for FTR-P1d.

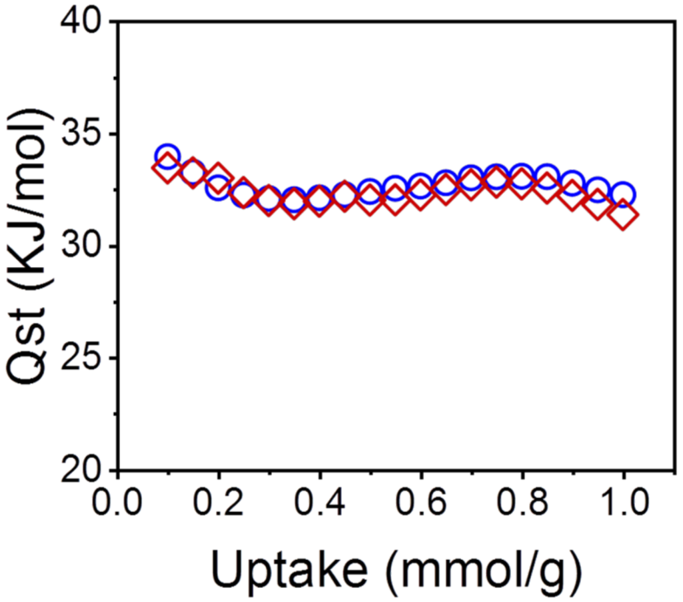

Figure S54. CO_2 isosteric heat of adsorption of FTR-P1 (blue circles) and FTR-P1d (red diamonds) measured *via* sorption-coupled microcalorimetry.

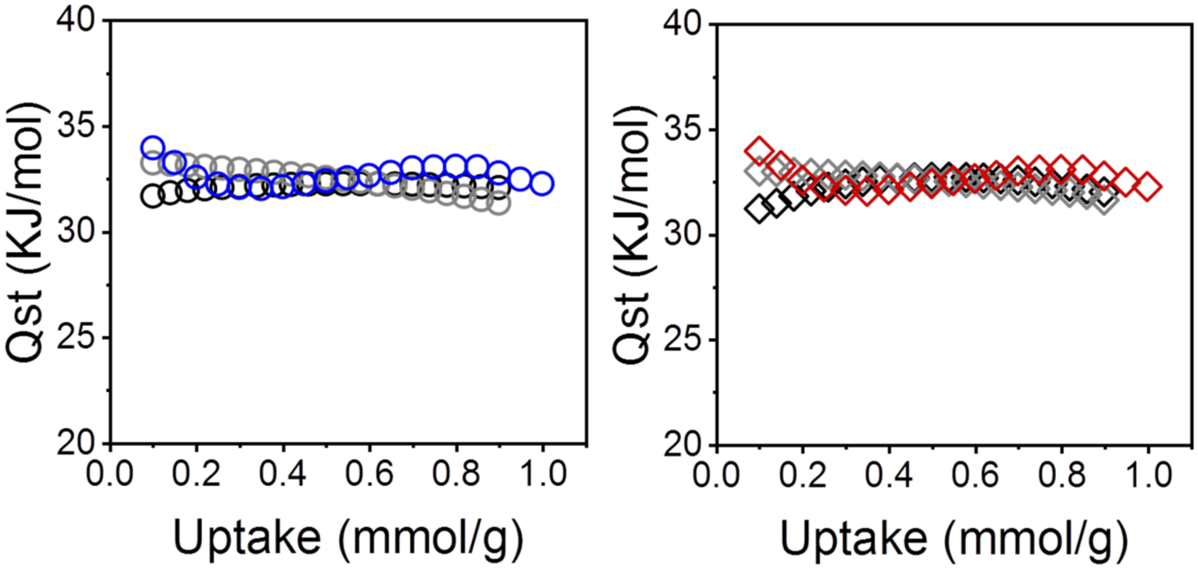

Figure S55. Left: comparison between CO_2 isosteric heat of adsorption of FTR-P1 calculated according to Langmuir (light grey), Langmuir-Freundlich (black) models and measured with sorption-coupled microcalorimetry (blue circles). Right: comparison between CO_2 isosteric heat of adsorption of FTR-P1d calculated according to Langmuir (light grey), Langmuir-Freundlich (black) models and measured with sorption-coupled microcalorimetry (red diamonds).

Table S12. Average isosteric heats of adsorption according to Langmuir and Langmuir-Freundlich models and measured with sorption-coupled microcalorimetry for FTR-P1 and FTR-P1d.

Q_{st} KJ/mol	Langmuir model	Langmuir-Freundlich model	Sorption-coupled calorimetry
FTR-P1	32.5	32.5	32.7
FTR-P1d	32.1	32.3	32.4

Simulations for determination of the gas arrangements and interaction energies. CO_2 arrangements were determined using GCMC fixed loading simulations using one molecule per unit-cell. DFT optimizations (CASTEP) were performed to obtain the energies used to determine the most probable CO_2 arrangements. The optimizations were performed using the GGA PBE functional with Grimme's DFT-D dispersion correction, and thresholds for geometry optimization and SCF convergence were chosen as 2 × 10–6 eV. Single point energies, calculated using CASTEP, were used to determine the interaction energies which were calculated as follows:

E(host+CO2):The full crystal structure with the guest molecules included.

$E(host)$: *The guest molecules are removed from the periodic model.*

$E(CO2)$: *The host molecules are removed from the periodic model.*

$Eint(host\text{–}CO2)$: *The host– guest interaction energy*

$$Eint(host\text{–}CO2) = E(host + CO2) - \big(E(host) + E(CO2)\big)$$

POWDER X-RAY DIFFRACTION AND PXRD STRUCTURAL RIETVELD REFINEMENT of FTR-P1d·4CO_2

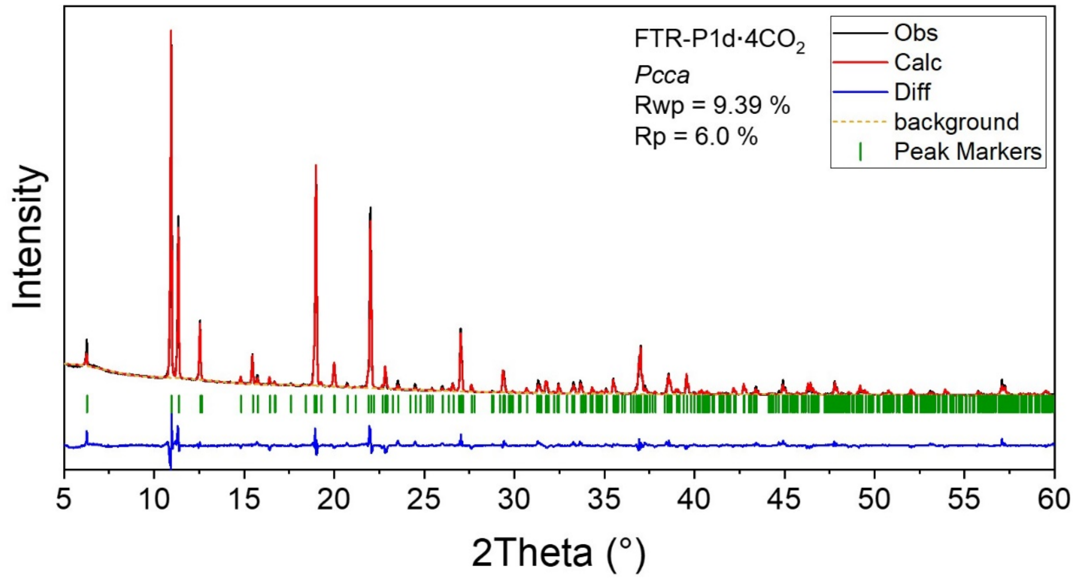

Figure S56. Rietveld fits of X-ray data for FTR-P1d·4CO_2, collected at 253 K under 2 bar CO_2, using the TOPAS-Academic-64 V6 software package.[6] The HKL indices are indicated as green markers and the difference plot are shown below. The black and red represent the observed and calculated traces respectively while the dotted yellow line represent the background plot.

Table S13. Unit cell parameters of FTR-P1d·4CO_2 from the Rietveld refinement of powder X-ray diffraction pattern collected at 253 K.

**FTR-P1d·4CO_2**	α'-phase
Temp	253 K
Space Group	*Pcca*
a (Å)	16.1167(4)
b (Å)	14.0720(4)
c (Å)	11.4375(3)
Cell Volume (Å^3)	2593.96(12)
ρ (g cm^{-3})	1.6337
Rwp	9.4 %
Rp	6.0 %

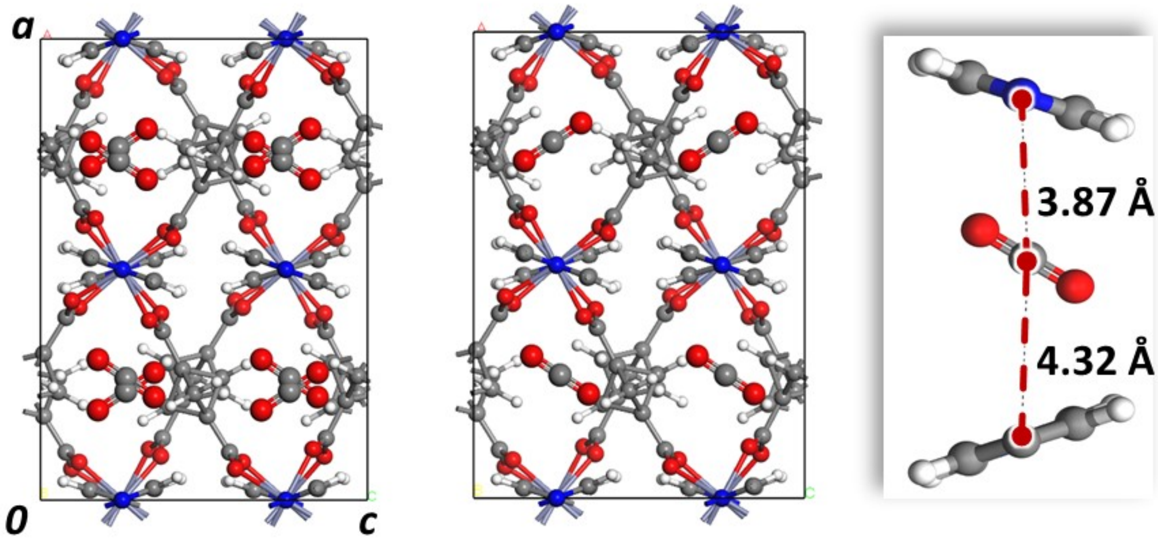

Figure S57. The unit-cell of contains 4 CO_2 molecules, each disorder over two equivalent positions. The structure are that of the *vacuo* α-phase. The CO_2 molecules are tilted with respect to one another which reduces the electrostatic repulsion between their oxygen atoms. The Host-CO_2 interaction energy, E_{int} = 32.7 kJ/mol. CO_2 molecules are confined off-centred between two bipyridine rings. The shorter centroid-to-carbon distance of 3.87 Å is associated with the bipy ring most parallel to the CO_2 molecule.

VARIABLE TEMPERATURE POWDER X-RAY DIFFRACTION (VT-PXRD) UNDER 1 BAR CO_2 ATMOSPHERE

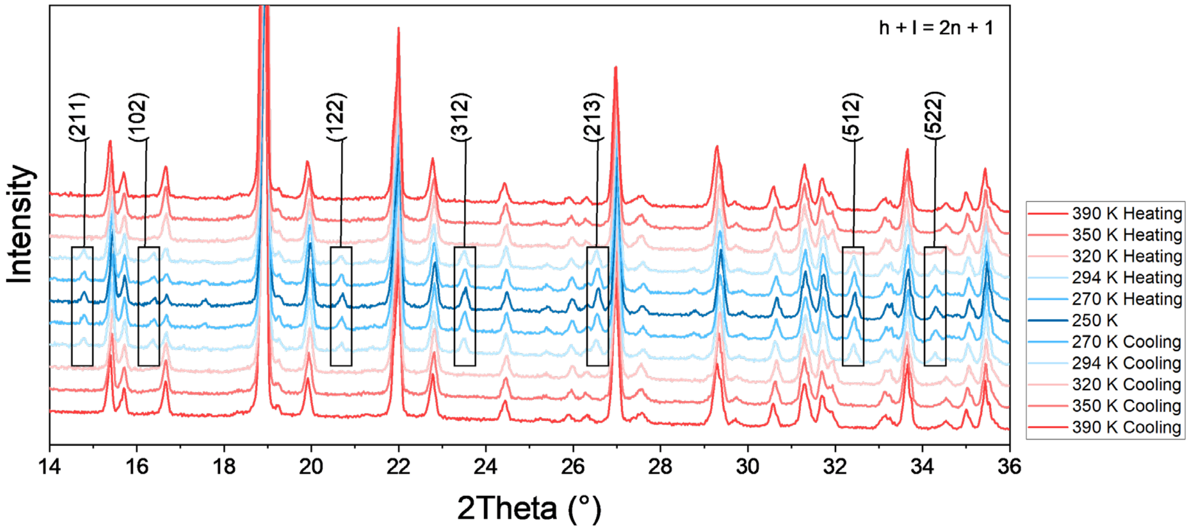

Figure S58. VT-PXRD cycle for FTR-P1d under 1 bar CO_2 atmosphere with a temperature range of 390 – 250 – 390 K. The red traces represent the β-phase while the α-phase is shown in blue. The additional peaks associated with the α-phase are highlighted with the black box and their corresponding miller indices.

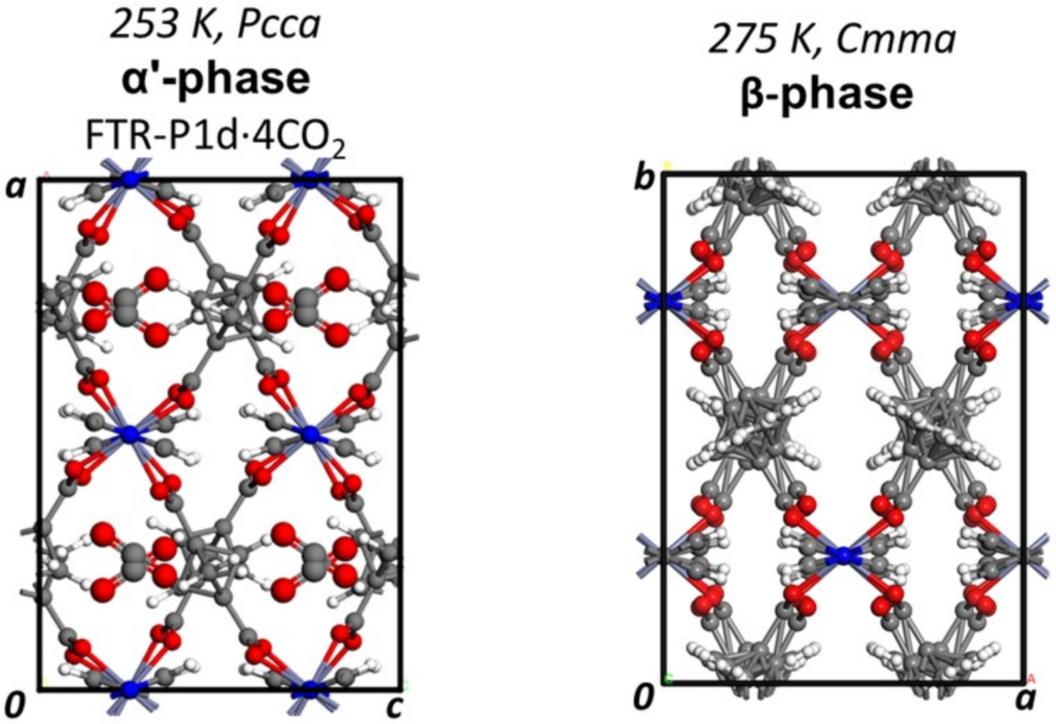

Figure S59. The α'-phase (253 K with 99% CO_2 occupancy, *Pcca*) and β-phase (275 K, *Cmma*) models of FTR-P1d, used for the two-phase Rietveld refinement of PXRD traces collected under I bar CO_2 at varying temperatures.

Table S14. The results of the phase-quantification of the α'-phase (blue diamonds, *Pcca*) and β-phase (red circles, *Cmma*) of FTR-P1d from the two-phase Rietveld refinement of PXRD traces collected under vacuum and varying temperatures. The CO_2 occupancies were extrapolated from the VT adsorption measurements and are shown as a percentage of the maximum (4 MPU). The total error in the phase percentage, Rwp and Rp of the final Rietveld refinement cycle are shown.

Temperature (K)	CO_2 occupancy (%)	Phase %		Error	Rwp (%)	Rp (%)
		α'-Phase	β-Phase			
390	0	10.3	89.7	1.23	8.0	5.7
350	0	11.5	88.5	4.3	8.1	5.8
320	17	24.8	75.2	3.2	7.7	5.4
294	57	88	12	2.5	7.4	5.4
270	77	89.1	10.9	2.21	7.4	5.5
250	89	90.5	9.5	2.35	8.3	6.0
250	89	91.1	8.9	1.3	8.2	5.9
270	77	90.32	9.68	1.4	8.0	5.7
294	57	87.17	12.83	4.2	7.2	5.3
320	17	20.953	79.047	5.8	8.6	6.1
350	0	15.25	84.75	1.75	8.1	5.7
390	0	9.228	90.772	2.1	8.2	5.8

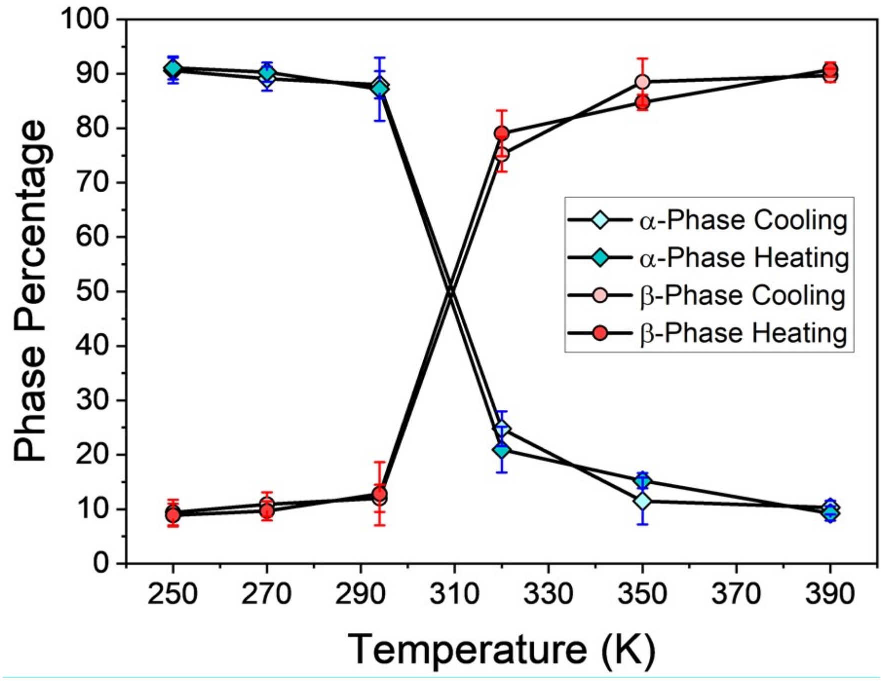

Figure S60. A plot of the phase-quantification of the α'-phase (blue diamonds, *Pcca* FTR-P1d·xCO_2) and β-phase (red circles, *Cmma* FTR-P1d) from the two-phase Rietveld refinement of PXRD traces collected under vacuum and varying temperatures. The error bars are shown in blue for the α-phase and in red for the β-phase.

Table S15. The results of the phase-quantification of the α'-phase (*Pcca*) and β-phase (*Cmma*) of FTR-P1d from the two-phase Rietveld refinement of PXRD traces collected at distinct CO_2 loadings and 293 K. The CO_2 occupancies were taken from an IGA sorption measurement and is give as a percentage of the maximum of 4 molecules per unit-cell (MPU). The total error in the phase percentage, Rwp and Rp of the final Rietveld refinement cycle are shown.

Pressure (bar)	CO2 occupancy (%)	Phase % α'-Phase	Phase % β-Phase	Error	Rwp (%)	Rp (%)
0	0	27.88	72.12	2.72	9.5	6.7
0.1	11	24.36	75.64	4.97	9.3	6.2
0.2	18	24.65	75.35	4.45	8.8	5.9
0.3	26	30.87	69.13	5.3	8.7	5.9
0.5	37	57.49	42.51	3.6	8.0	5.4
0.7	47	60.6	39.4	5.9	7.0	4.9
1	60	70.09	29.91	4	6.5	4.8
1.5	69	68.77	31.23	5.9	5.9	4.4
2.0	77	69.8	30.2	1.6	5.8	4.1

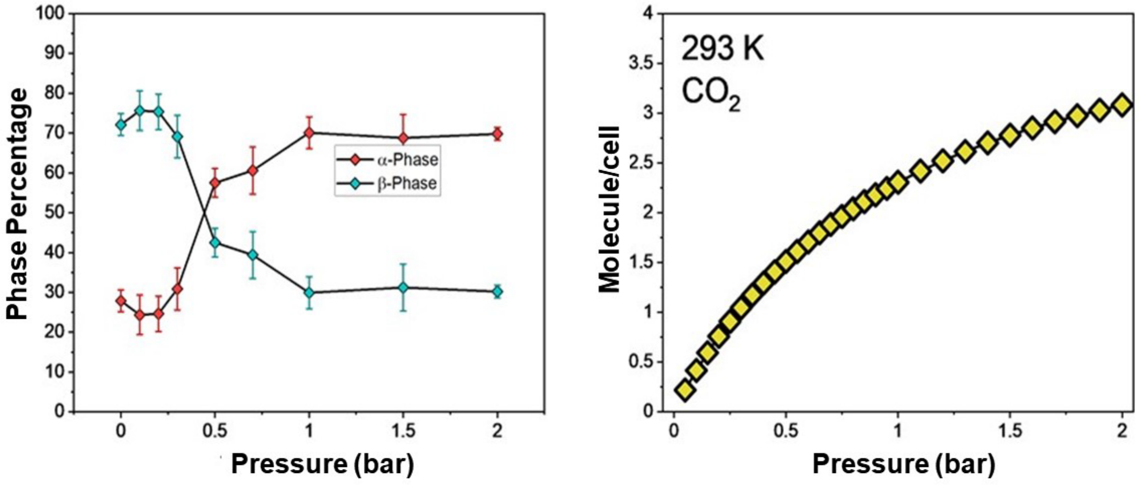

Figure S61. Left – CO_2 Sorption isotherm collected on the IGA gravimetric sorption analyser at 293K up to 2 bar. Right - A plot of the phase-quantification of the α'-phase (red diamonds, *Pcca* FTR-P1d·*x*CO_2) and β-phase (blue diamonds, *Cmma* FTR-P1d) from the two-phase Rietveld refinement of PXRD traces collected under 293 K and varying pressures (0 – 2 bar CO_2). The error bars are shown in red for the α-phase and in blue for the β-phase.

^{2}H NMR RESULTS FOR FTR-P1d·xCO$_2$

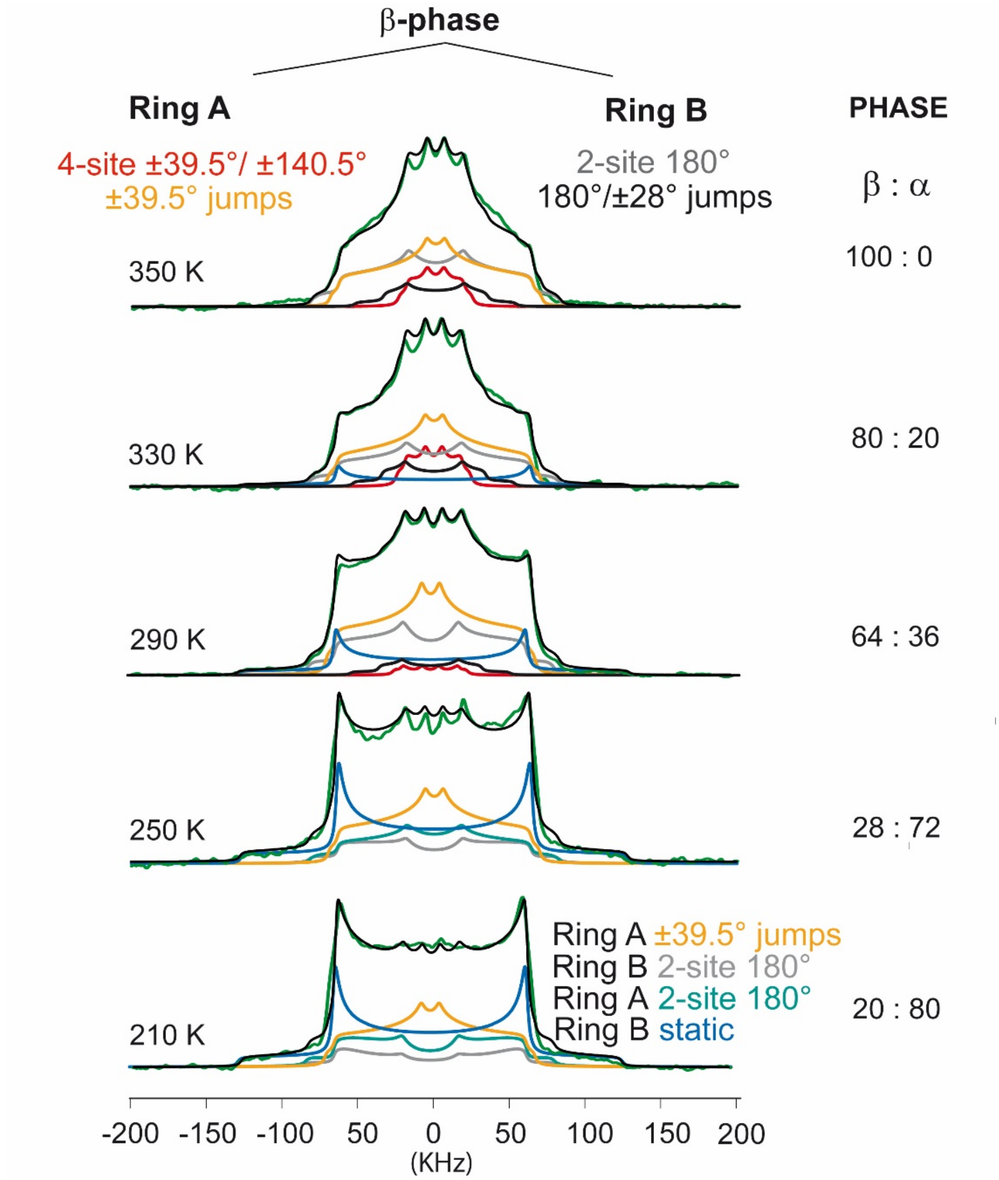

Figure S62. ^{2}H solid-echo NMR spectra of bipyridine rings in FTR-P1d·xCO$_2$ at 3 bar from ^{2}H NMR analysis (α'-phase blue circles, β-phase red circles) at distinct temperatures and the simulated spectra considering the following components: 4-site ±39.5°/ ±140.5° reorientation for ring A in the β-phase (red line), 2 site 180° reorientation for ring B in the β-phase (gray line), 180°/±28° jumps for ring B in the β-phase (black line), 2 site 180° reorientation for ring A in the α'-phase (green line), static pattern for ring B in the α'-phase (blue line) and 180°±39.5° jumps for ring A in both phases (yellow line).

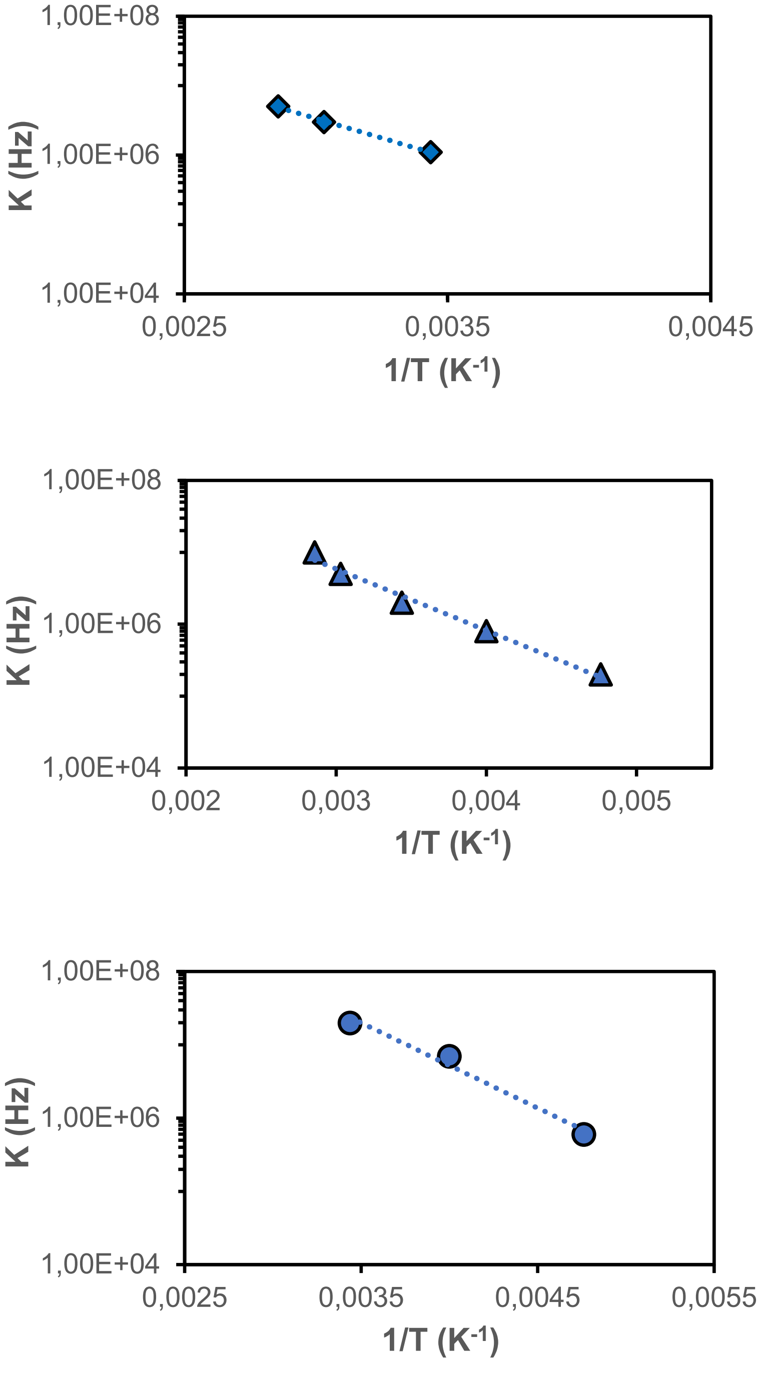

Figure S63. Arrhenius plots of reorentation frequencies versus the inverse of temperature from ^{2}H NMR spectra of FTR-P1d· $x$$CO_2$ at 3 bar from ^{2}H NMR analysis (α'-phase blue circles, β-phase red circles). E_a=5.1 kcal/mol for 4-site flip reorentation ±39.5°/±140.5° of ring A in the α-phase (blue diamonds), E_a=3.9 kcal/mol for 2-site 180°C flip reorientation for ring B in the α-phase (blue triangles), E_a=5.3 kcal/mol for 2-site 180° flip reorientation of ring A in the β-phase (blue circles). The error bars on temperature are smaller than the symbols.

Table S16. Reorentational frequencies (k) of bipyridine rings from ^{2}H solid-echo NMR lineshape analysis of FTR-P1d·$x$$CO_2$ at 3 bar.

Temperature	β-phase	β- phase	α'- phase	α'- phase
	ring A	ring B	ring A	ring B
	4-site ±39.5°±140.5°	2-site 180°	2-site 180°	
(K)	(Hz)	(Hz)	(Hz)	(Hz)
350.0 ± 0.2	5.00E+06	1.00E+07		
330.0 ± 0.2	3.00E+06	5.00E+06		< 1.00E+04
291.0 ± 0.2	1.10E+06	2.00E+06	2.00E+07	< 1.00E+04
250.0 ± 0.2		8.00E+05	7.00E+06	< 1.00E+04
210.0 ± 0.2		2.00E+05	6.00E+05	< 1.00E+04

Table S17. Activation energies of bipy rings for FTR-P1d·$x$$CO_2$.

	β-phase	β- phase	α'- phase	α'- phase
	ring A	ring B	ring A	ring B
	4-site ±39.5°/±140.5° (+ jumps ±39.5°)	2-site 180°	2-site 180°	
E_a (kcal//mol)	**5.15**	**3.9**	**5.33**	**-**
K_0 (Hz)	7.9E+09	2.1E+09	2.3E+11	-
$\Delta H^{\neq}$ (kcal/mol)	4.51	3.4	4.8	
$\Delta S^{\neq}$ (cal/mol K)	-15	-18	-8	

Table S18. Fraction (%) of distinct motional mechanisms at different temperatures for bipyridine rings A and B in FTR-P1d·$x$$CO_2$ as obtained from ^{2}H solid-echo NMR spectra.

		β-phase		α'-phase	
**Temp.**	**Mechanism**	**ring A**	**ring B**	**ring A**	**ring B**
350.0 ± 0.2 K	4-site ±39.5°/±140.5°	10%			
	2-site 180°/ ±28°		10%		
	jump ±39.5°	40%			
	2-site 180°		40%		
330.0 ± 0.2 K	4-site ±39.5°/±140.5°	10%			
	2-site 180°/ ±28°		10%		
	jump ±39.5°	30%		10%	
	2-site 180°		30%		
	static				10%
291.0 ± 0.2 K	4-site ±39.5°/±140.5°	2%			
	2-site 180°/ ±28°		5%		
	jump ±39.5°	28%		15%	
	2-site 180°		30%	2%	
	static				18%
250.0 ± 0.2 K	4-site ±39.5°/±140.5°				
	2-site 180°/ ±28°				
	jump ±39.5°	14%		17%	
	2-site 180°		14%	19%	
	static				36%
210.0 ± 0.2 K	4-site ±39.5°/±140.5°				
	2-site 180°/ ±28°				
	jump ±39.5°	10%		20%	
	2-site 180°		10%	20%	
	static				40%

Tables S19. Fraction of α'- and β-phases as function of temperature of FTR-P1d·xCO_2 at 3 bar from ^{2}H NMR analysis and the corresponding estimated errors.

Temperature (K)	α'-phase	Estimated error	β-phase	Estimated error
350	3	2.8	97	2.8
330	20	3.8	80	3.8
291	35	4.5	64	4.5
250	72	3.4	28	3.4
210	80	3	20	3

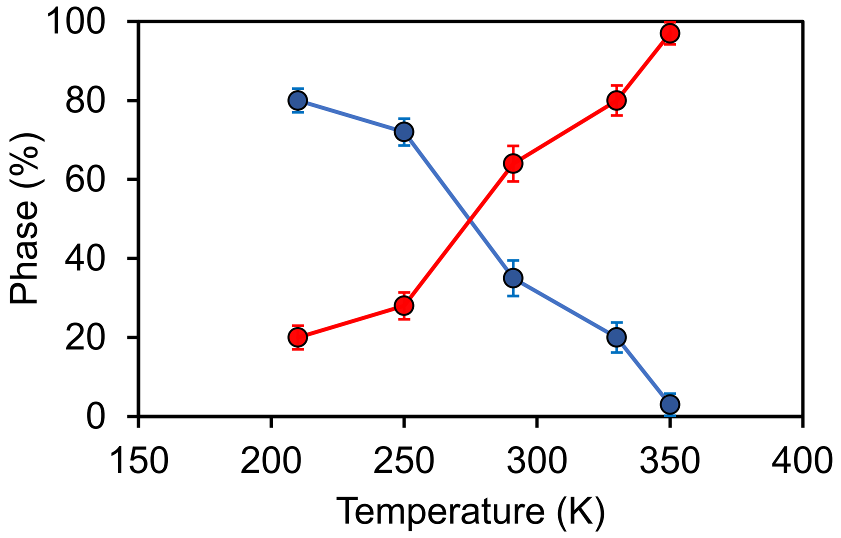

Figure S64. Fraction of α'- to β-phases as function of temperature of FTR-P1d·xCO_2 at 3 bar from ^{2}H NMR analysis (α'-phase blue circles, β-phase red circles).

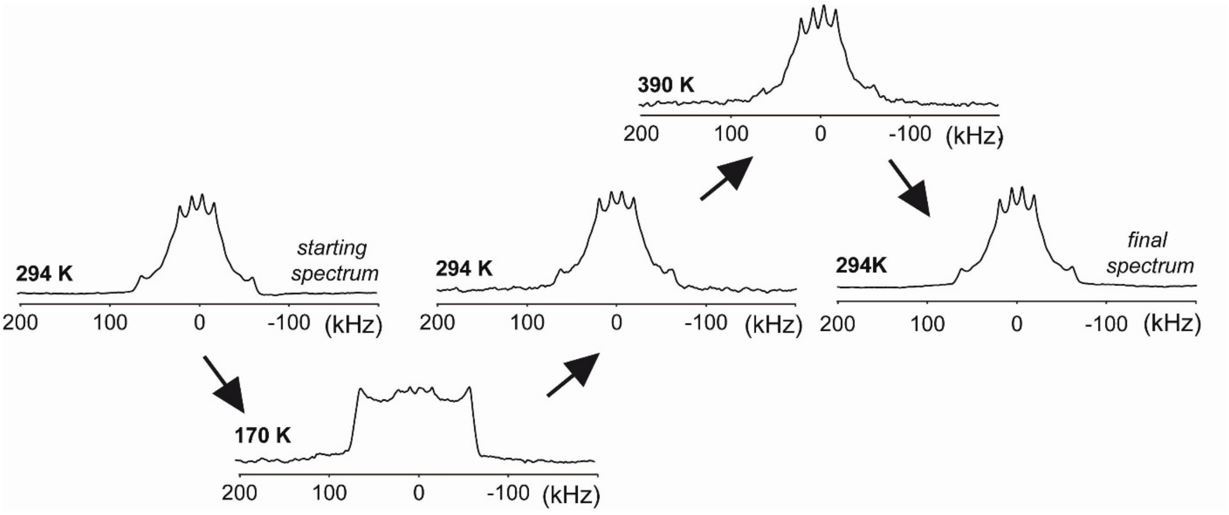

Figure S65. ^{2}H MAS spectra of FTR-P1d·$x$$CO_2$ collected at variable temperature as function of time, showing the complete reversibility of dynamical process.

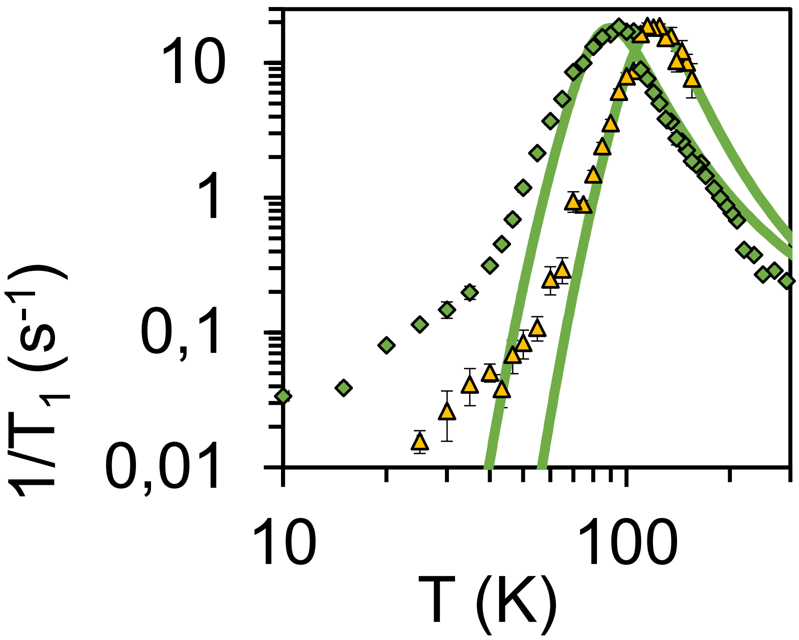

Figure S66. ^{1}H T_1 relaxation times as function of temperature of FTR-P1d. The error bars on temperature are smaller than the symbols' width.

PXRD and ^{2}H NMR of FTR-P1d*DMF

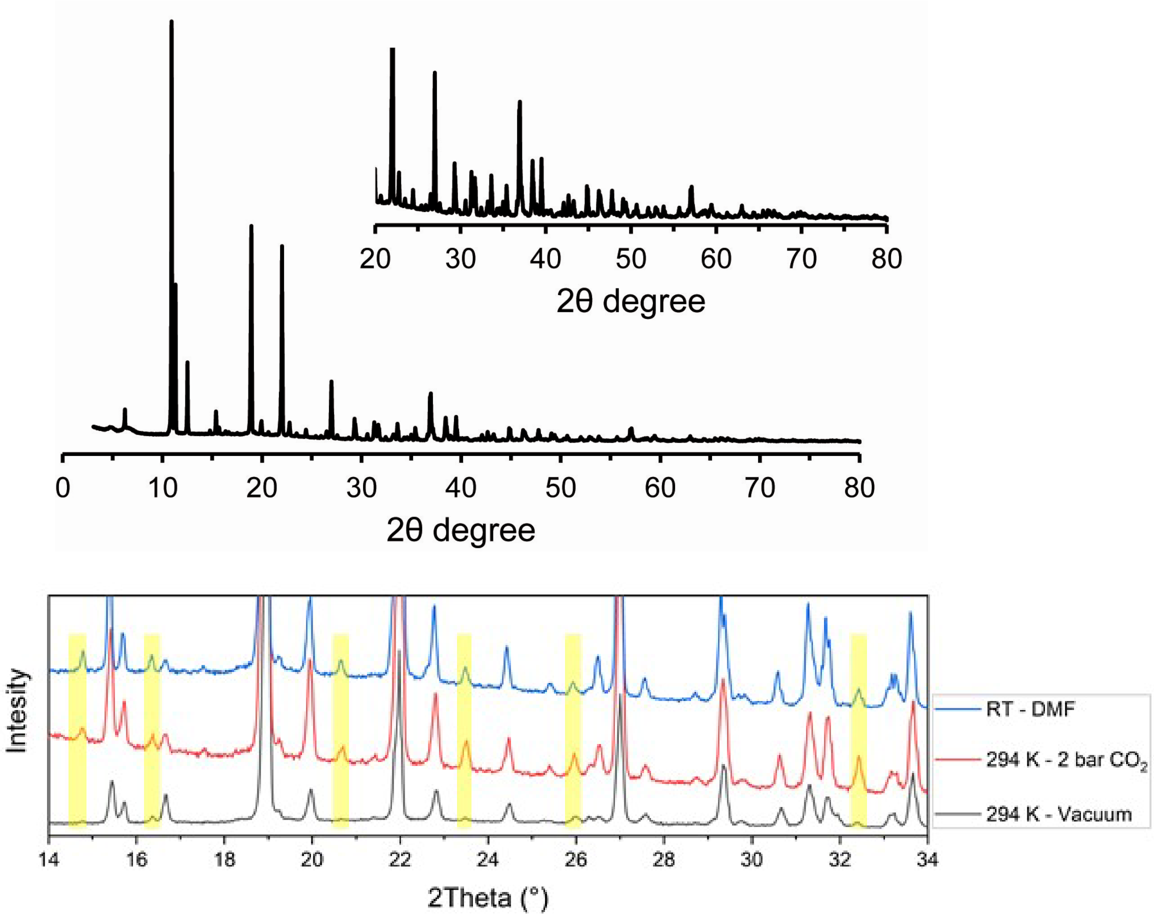

Figure S67. PXRD profile of FTR-P1d*DMF (top). PXRD oof FTR-P1d*DMF compared to those of of FTR-P1d*CO_2 and FTR-P1d.

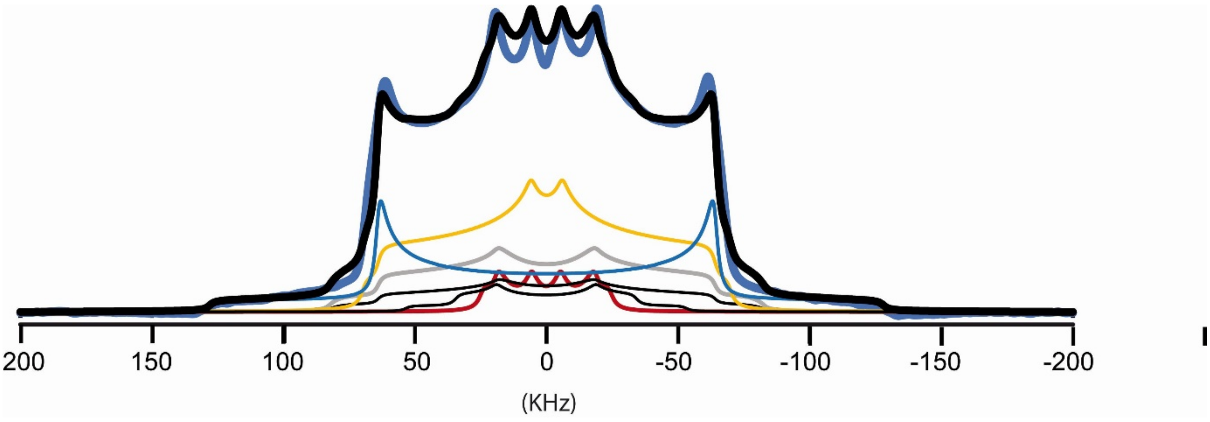

Figure S68. ^{2}H NMR of of FTR-P1d*DMF: experimental (blue) and simulated (black) lines. Single components: 4-site ±39.5°/±140.5° (red), 2-site 180°/ ±28° (black), jump ±39.5° (yellow) and 2-site 180° (grey/black).

Tables S20. Fraction (%) of distinct motional mechanisms at different temperatures for bipyridine rings A and B in FTR-P1d*DMF as obtained from ^2H solid-echo NMR spectra.

		β-phase		α'-phase	
**Temp.**	**Mechanism**	**ring A**	**ring B**	**ring A**	**ring B**
290.0 ± 0.2 K	4-site ±39.5°/±140.5°	5% $1.1*10^6$ Hz			
	2-site 180°/ ±28°		5% $1*10^7$ Hz		
	jump ±39.5°	10% $6*10^6$ Hz		25% $6*10^6$ Hz	
	2-site 180°		30% $9*10^6$ Hz		25% $<10^4$ Hz

MOLECULAR MECHANICS (MM) 2D SCANS

The 2D scans for rotor pairs **R1-R2** and **R1-R3** were considered, like the DFT calculation. The calculation was performs considering periodic boundary conditions using a 2 x 2 supercell of both FTR-P1d (Empty, **α-phase**) or FTR-P1d·4CO_2 (**α'-phase**). The computational models are shown in Figure S69. The metal-carboxylate paddle-wheel node was treated as a rigid body, i.e., the internal coordinates of the paddle-wheel atoms are preserved during the optimization. The unit-cell parameters were free to optimize at each step of the scan. The scan was performed from 0° - 360° with a step size of 5° for the 1st scan parameter (rotational torsion angle of **R1**, Figure S69) and 0° - 240° with a step size of 5° for the 2nd scan parameter (rotational torsion angle of **R2** and **R3**). Since rotor has a 3-fold symmetry, the energies repeat every 120° and thus the 2nd scan parameter could be extended to 360° by using the data calculated between 0° - 240°.

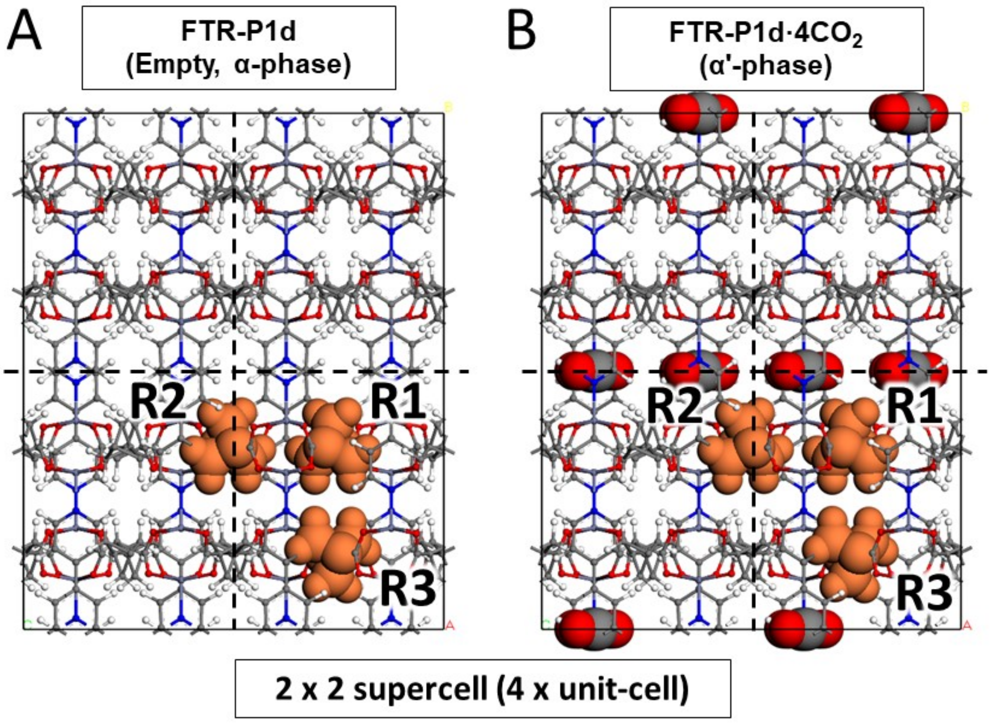

Figure S69. The computational models used for the molecular mechanics 2D scan for FTR-P1d (Empty, **α-phase**) or FTR-P1d·4CO_2 (**α'-phase**). The scanning rotors R1, R2 and R3 are indicated. The scan was performed in pair of only **R1-R2** and **R1-R3**, similar to the DFT calculations.

COMPARISON BETWEEN DFT AND MOLECULAR MECHANICS 2D SCANS

DFT - Reduce model comprising only FTR moieties as positioned within the crystal structure.

MM - Periodic model comprising a 2 x 2 supercell (4 x unit-cell)

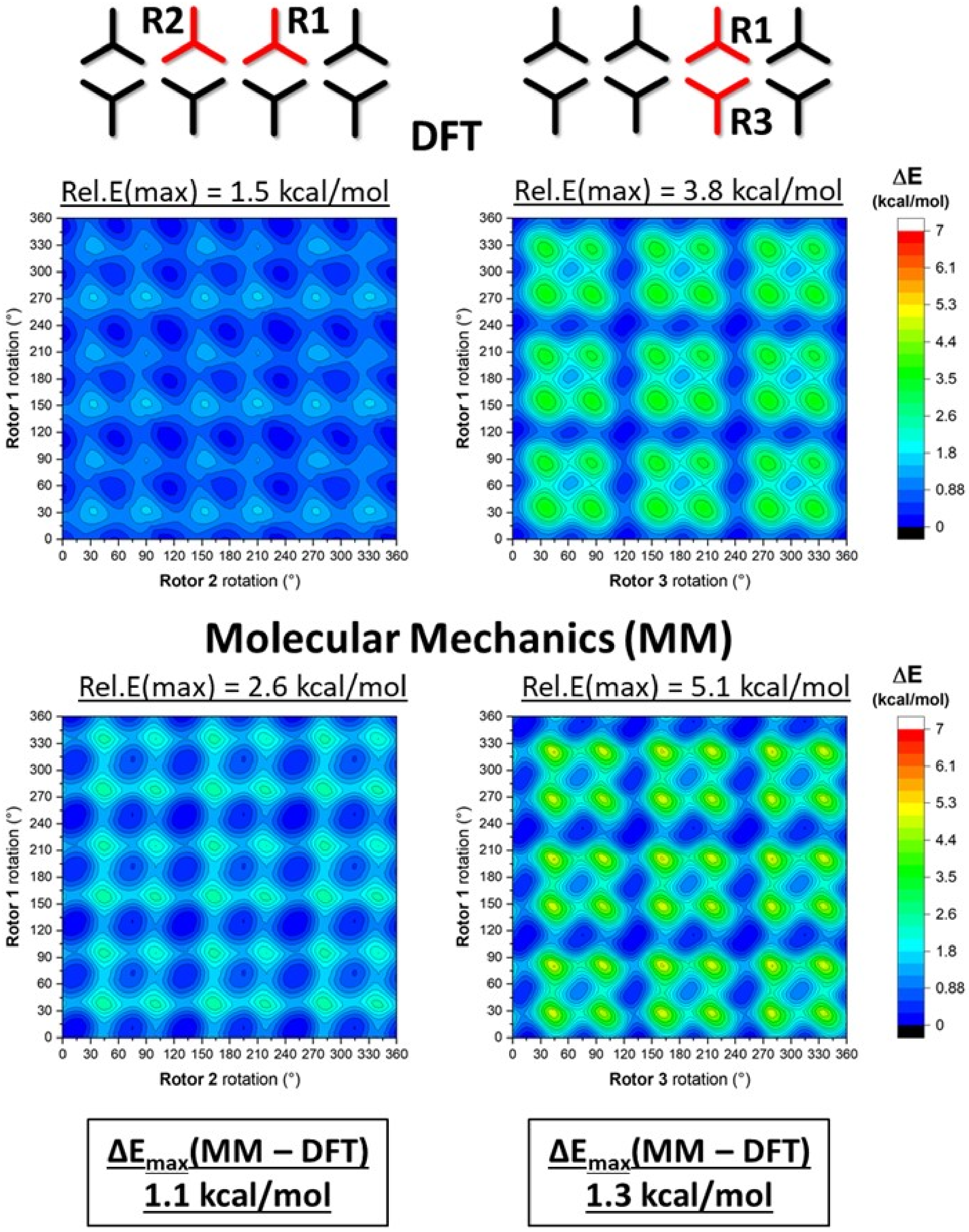

Figure S70. Comparison of the DFT and molecular mechanics (MM) 2D scans for the rotor pairs **R1-R2** and **R1-R3**. The 2D contour maps are plotted on the same ΔE scale (0 – 7 kcal/mol) and contour levels for direct visual qualitative comparison. The MM calculations are observed to be ca. 1 kcal/mol higher than the DFT 2D scans.

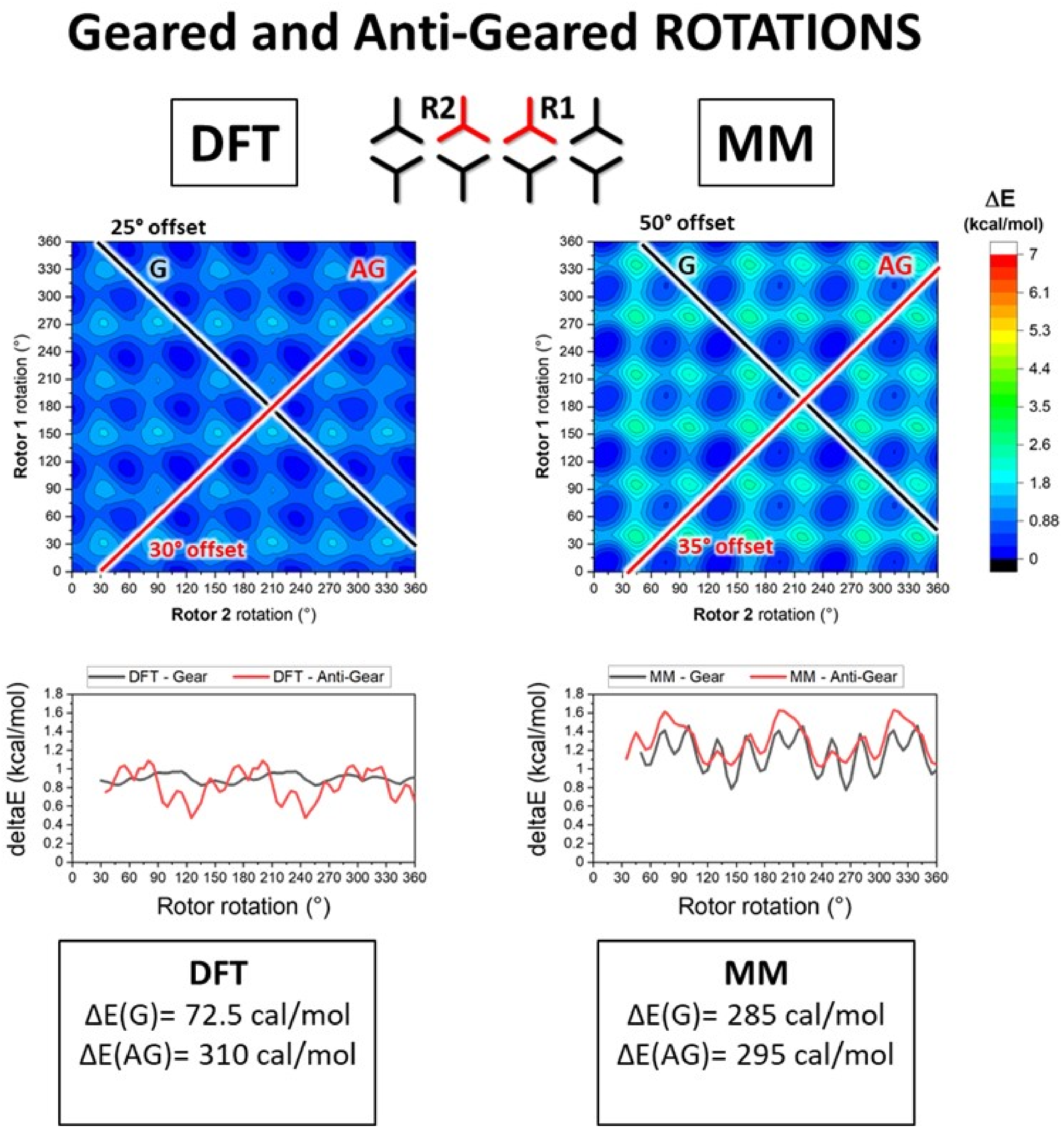

Figure S71. The 2D DFT scans results for **R1-R2** as calculated by molecular mechanics (2 x 2 supercell) and compared to the DFT calculations (reduced model comprising FTR rotors). Top: graphical illustration of the model. Middle: The 2D contour maps with the rotation of rotors pairs R1 and R2 on the Y- and X-axes with the colours indicating the ΔE. The colour scale ranges from 0 kcal/mol (blue) to 7 kcal/mol (red). This energy range was chosen for comparison to the **R1-R3** 2D scans. The lines indicate the most optimal Gearing (G, clockwise and counterclockwise rotations, i.e., the rotors rotate in opposite directions) and Anti-gearing (AG, clockwise - clockwise and counterclockwise - counterclockwise rotations, i.e., the rotors rotate in the same directions) pathways on the energy surfaces, with the relative rotor rotational offset indicated for each. Bottom: The ΔE plot for the most optimal gearing and anti-gearing rotations. The DFT and MM ΔE (energy barrier) for G and Ag are shown per rotor.

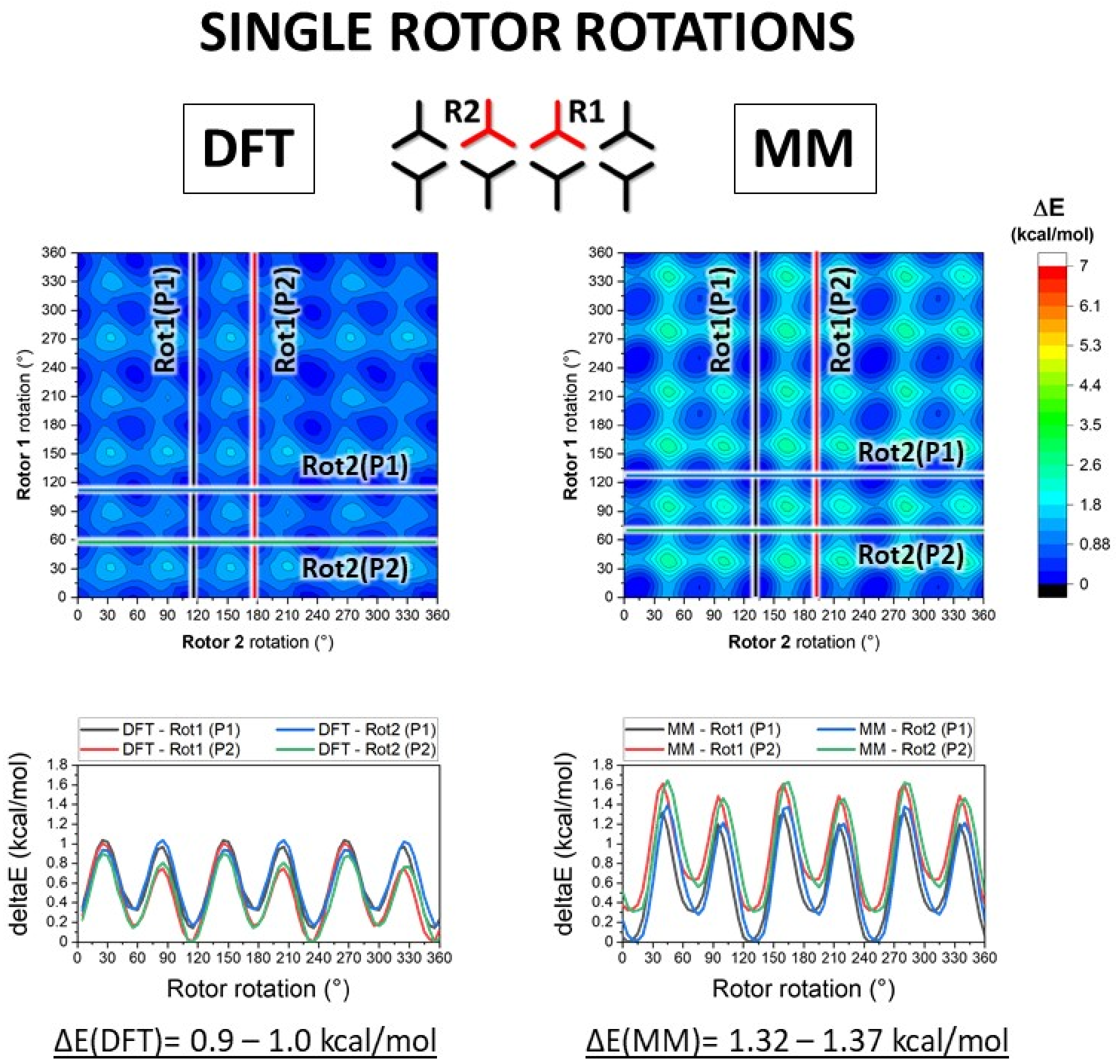

Figure S72. The 2D DFT scans results for **R1-R2** as calculated by molecular mechanics (2 x 2 supercell) and compared to the DFT calculations (reduced model comprising FTR rotors). Top: graphical illustration of the model. Middle: The 2D contour maps with the rotation of rotors pairs R1 and R2 on the Y- and X-axes with the colours indicating the ΔE. The colour scale ranges from 0 kcal/mol (blue) to 7 kcal/mol (red). This energy range was chosen for comparison to the **R1-R3** 2D scans. The lines indicate the most optimal single rotor rotation pathways on the energy surfaces. Bottom: The ΔE plot for the most optimal single rotor rotations. The DFT and MM ΔE (energy barrier) are shown per rotor. **Pathway 1 (P1):** The rotation rotor **R1** with R2 in the Global minimum position (0 - 15°) and the rotation rotor **R2** with R1 in the Global minimum position (0 - 15°). **Pathway 2 (P2):** The rotation rotor **R1** with R2 in the Local minimum position (60 - 75°) and the rotation rotor **R2** with R1 in the Local minimum position (60 - 75°).

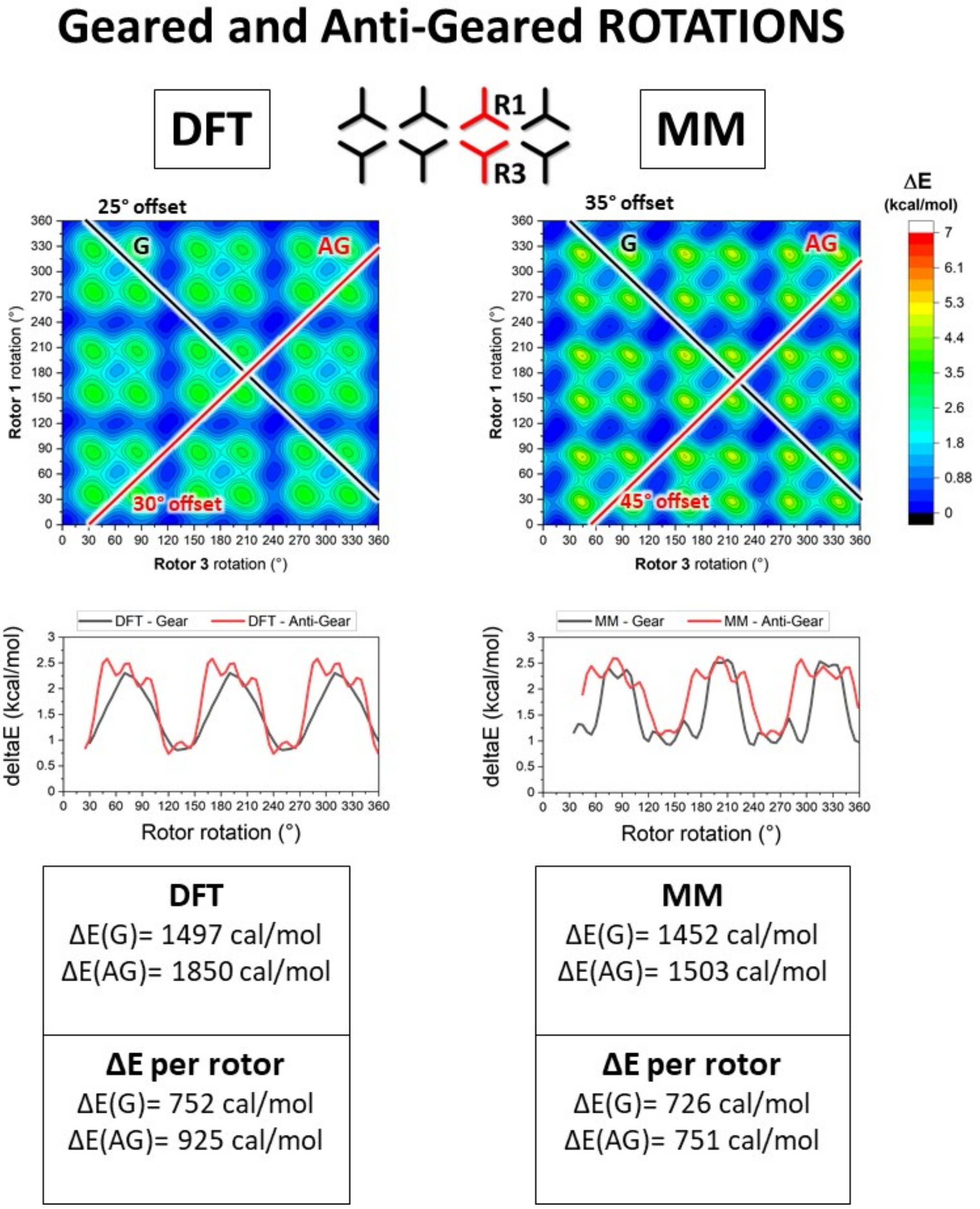

Figure S73. The 2D DFT scans results for **R1-R3** as calculated by molecular mechanics (2 x 2 supercell) and compared to the DFT calculations (reduced model comprising FTR rotors). Top: graphical illustration of the model. Middle: The 2D contour maps with the rotation of rotors pairs R1 and R2 on the Y- and X-axes with the colours indicating the ΔE. The colour scale ranges from 0 kcal/mol (blue) to 7 kcal/mol (red). The lines indicate the most optimal Gearing (G, clockwise and counterclockwise rotations, i.e., the rotors rotate in opposite directions) and Anti-gearing (AG, clockwise - clockwise and counterclockwise - counterclockwise rotations, i.e., the rotors rotate in the same directions) pathways on the energy surfaces, with the relative rotor rotational offset indicated for each. Bottom: The ΔE plot for the most optimal gearing and anti-gearing rotations. The DFT and MM ΔE (energy barrier) for G and Ag are shown per rotor.

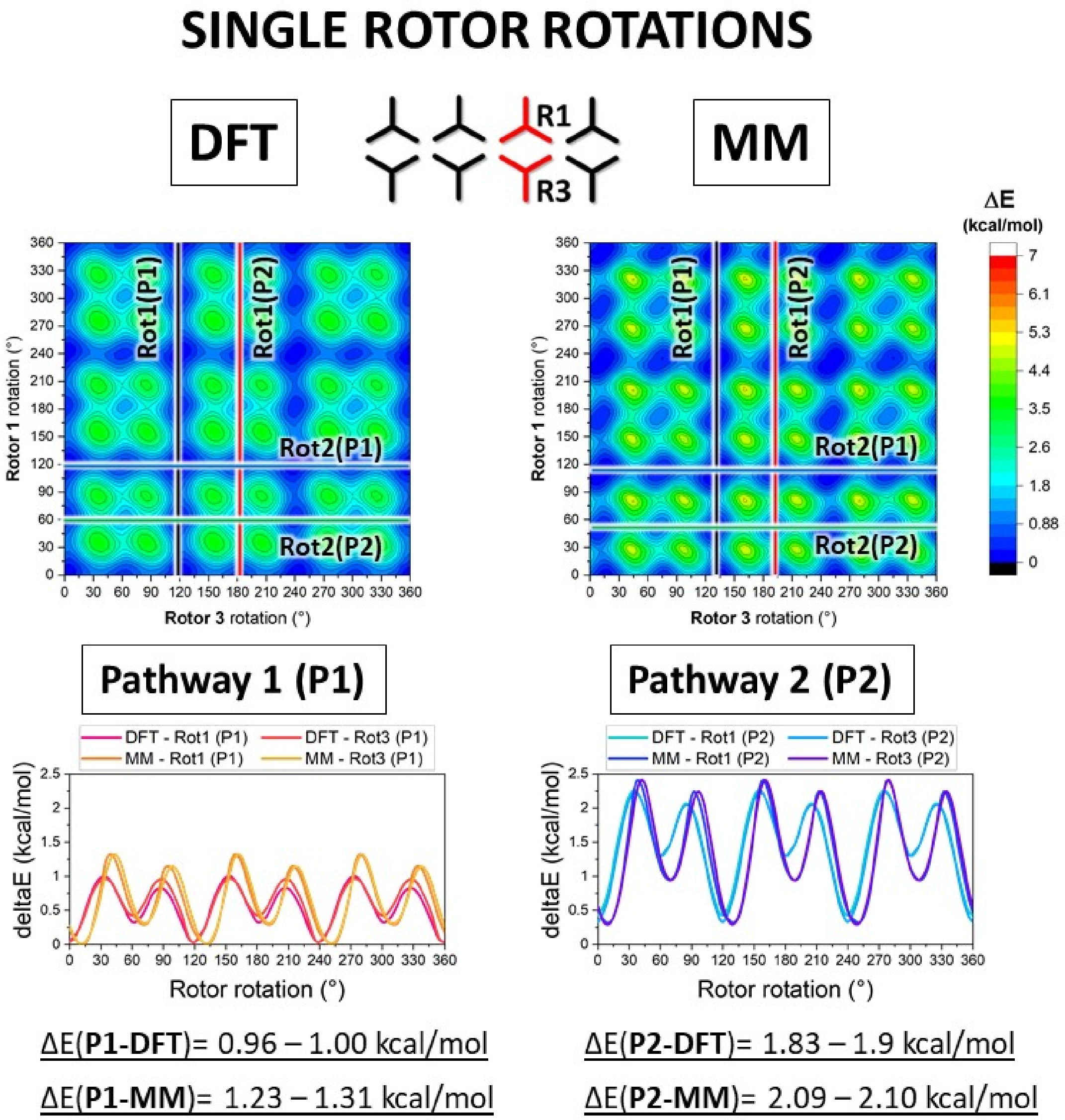

Figure S74. The 2D DFT scans results for **R1-R3** as calculated by molecular mechanics (2 x 2 supercell) and compared to the DFT calculations (reduced model comprising FTR rotors). Top: graphical illustration of the model. Middle: The 2D contour maps with the rotation of rotors pairs R1 and R2 on the Y- and X-axes with the colours indicating the ΔE. The colour scale ranges from 0 kcal/mol (blue) to 7 kcal/mol (red). The lines indicate the most optimal single rotor rotation pathways on the energy surfaces. Bottom: The ΔE plot for the most optimal single rotor rotations. The DFT and MM ΔE (energy barrier) are shown per rotor. **Pathway 1 (P1):** The rotation rotor **R1** with R2 in the Global minimum position (0 - 15°) and the rotation rotor **R3** with R1 in the Global minimum position (0 - 15°). **Pathway 2 (P2):** The rotation rotor **R1** with R2 in the Local minimum position (60 - 75°) and the rotation rotor **R3** with R1 in the Local minimum position (60 - 75°).

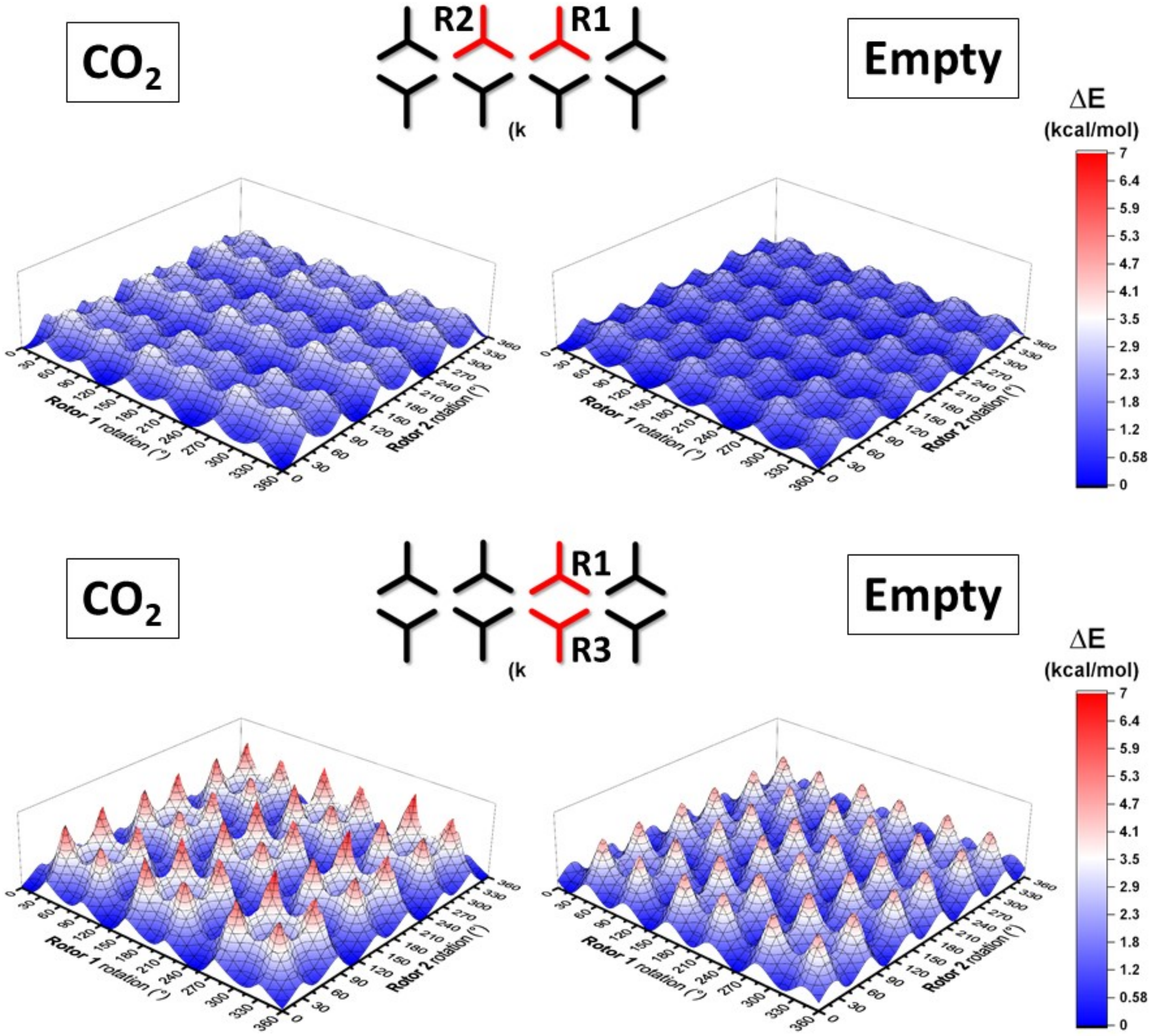

Figure S75. Comparison of **FTR-P1d** (Empty, α-phase) and **FTR-P1d·4CO₂** (CO_2 loaded, α'-phase) 2D scans for the rotor pairs **R1-R2** and **R1-R3**. The 2D surface maps are plotted on the same ΔE scale (0 – 7 kcal/mol) and contour levels for direct visual qualitative comparison.

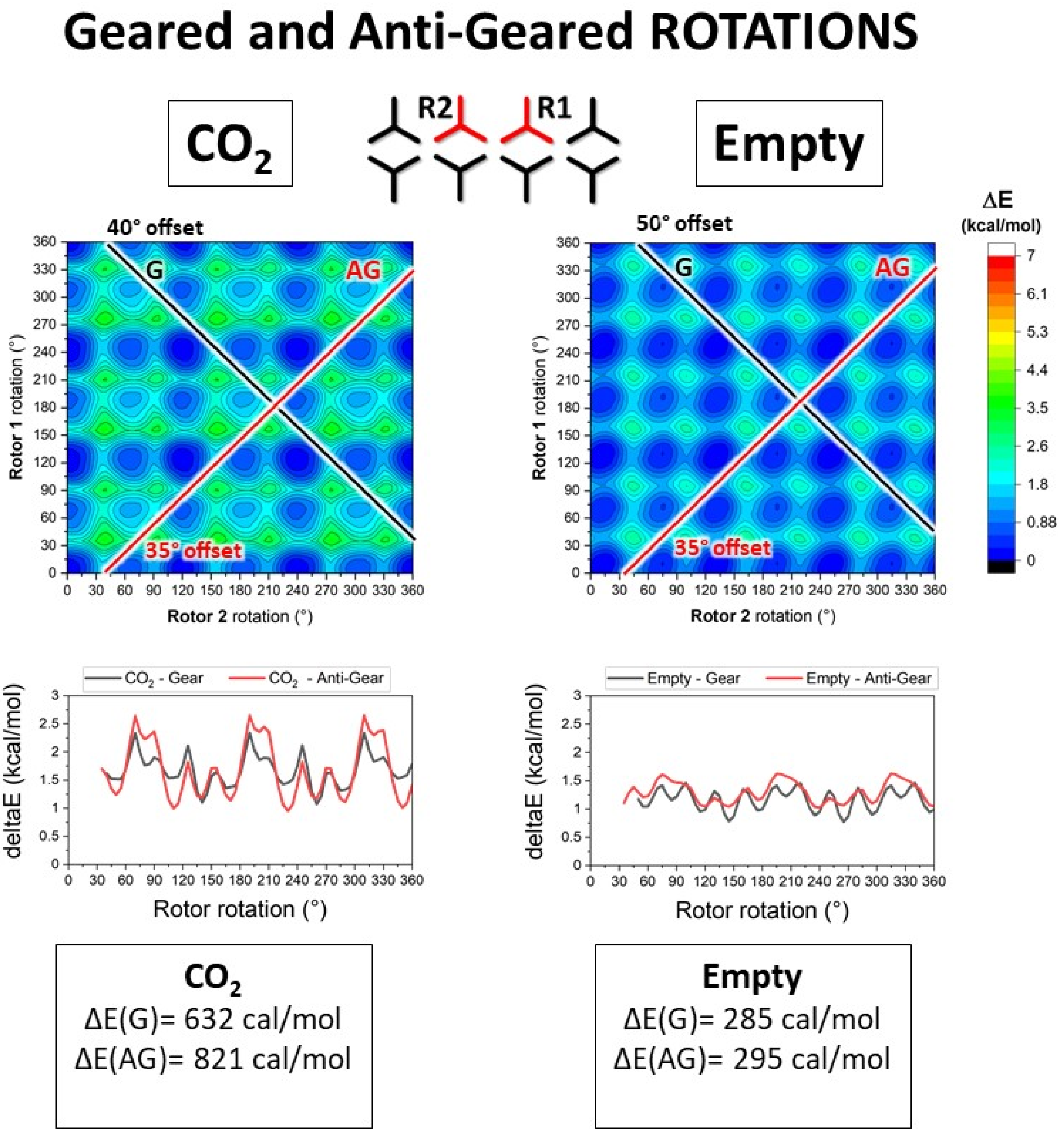

Figure S76. The 2D DFT scans results for **R1-R2** as calculated by molecular mechanics (2 x 2 supercell) for the empty (**FTR-P1d**, α-phase) and CO_2 loaded (**FTR-P1d·4CO$_2$**, α'-phase) structures. Top: graphical illustration of the model. Middle: The 2D contour maps with the rotation of rotors pairs **R1** and **R2** on the Y- and X-axes with the colours indicating the ΔE. The colour scale ranges from 0 kcal/mol (blue) to 7 kcal/mol (red). This energy range was chosen for comparison to the **R1-R3** 2D scans. The lines indicate the most optimal Gearing (G) and Anti-gearing (AG) pathways on the energy surfaces, with the relative rotor rotational offset indicated for each. Bottom: The ΔE plot for the most optimal gearing and anti-gearing rotations. The DFT and MM ΔE (energy barrier) for G and AG are shown per rotor.

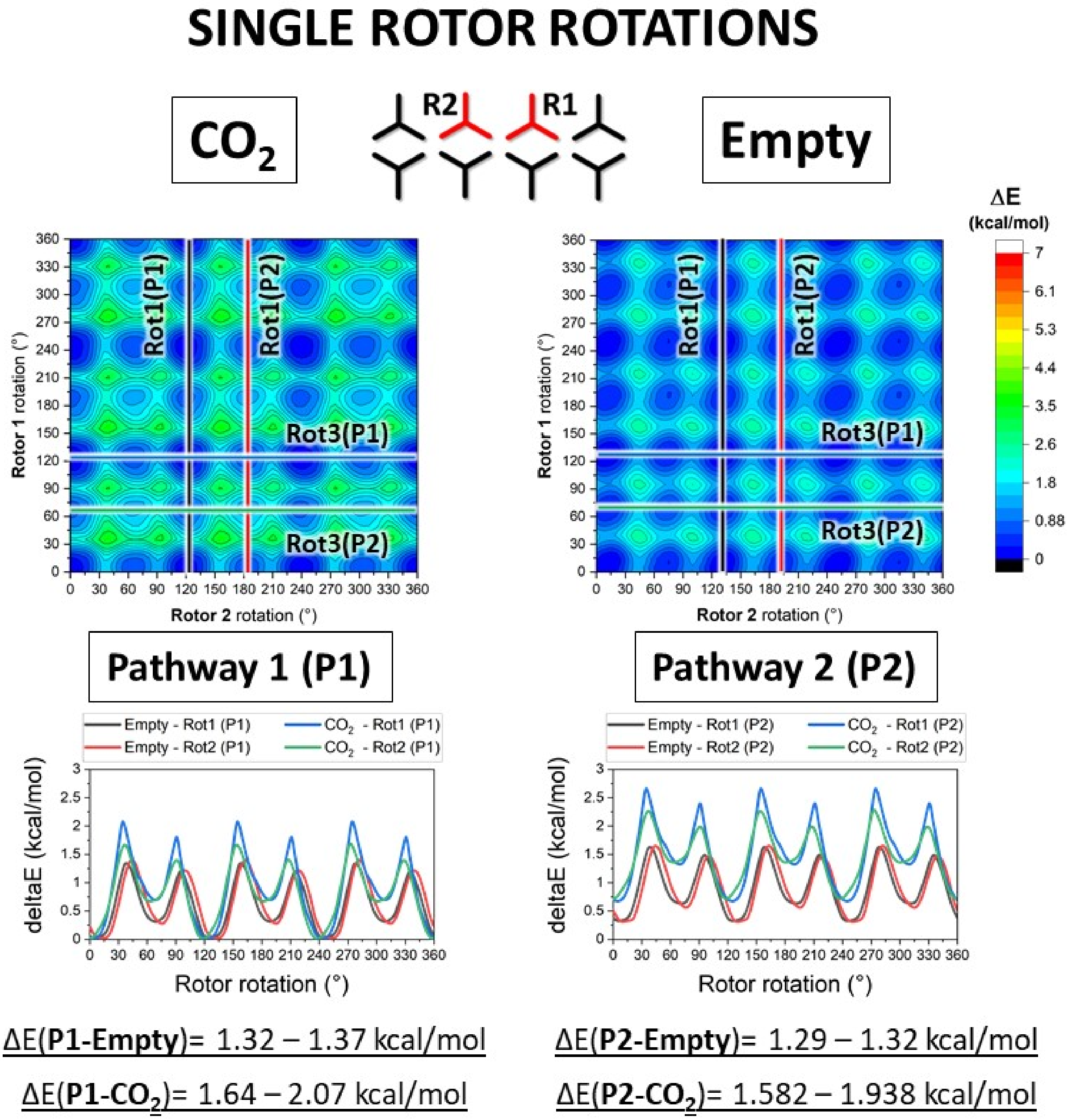

Figure S77. The 2D DFT scans results for **R1-R2** as calculated by molecular mechanics (2 x 2 supercell) for the empty (**FTR-P1d**, α-phase) and CO_2 loaded (**FTR-P1d·4CO₂**, α'-phase) structures. Top: graphical illustration of the model. Middle: The 2D contour maps with the rotation of rotors pairs **R1** and **R2** on the Y- and X-axes with the colours indicating the ΔE. The colour scale ranges from 0 kcal/mol (blue) to 7 kcal/mol (red). The lines indicate the most optimal single rotor rotation pathways on the energy surfaces. Bottom: The ΔE plot for the most optimal single rotor rotations. The **FTR-P1d·4CO₂** and **FTR-P1d** ΔE (energy barrier) are shown per rotor. **Pathway 1 (P1):** The rotation rotor **R1** with R2 in the Global minimum position (0 - 15°) and the rotation rotor **R2** with **R1** in the Global minimum position (0 - 15°). **Pathway 2 (P2):** The rotation rotor **R1** with **R2** in the Local minimum position (60 - 75°) and the rotation rotor **R2** with **R1** in the Local minimum position (60 - 75°).

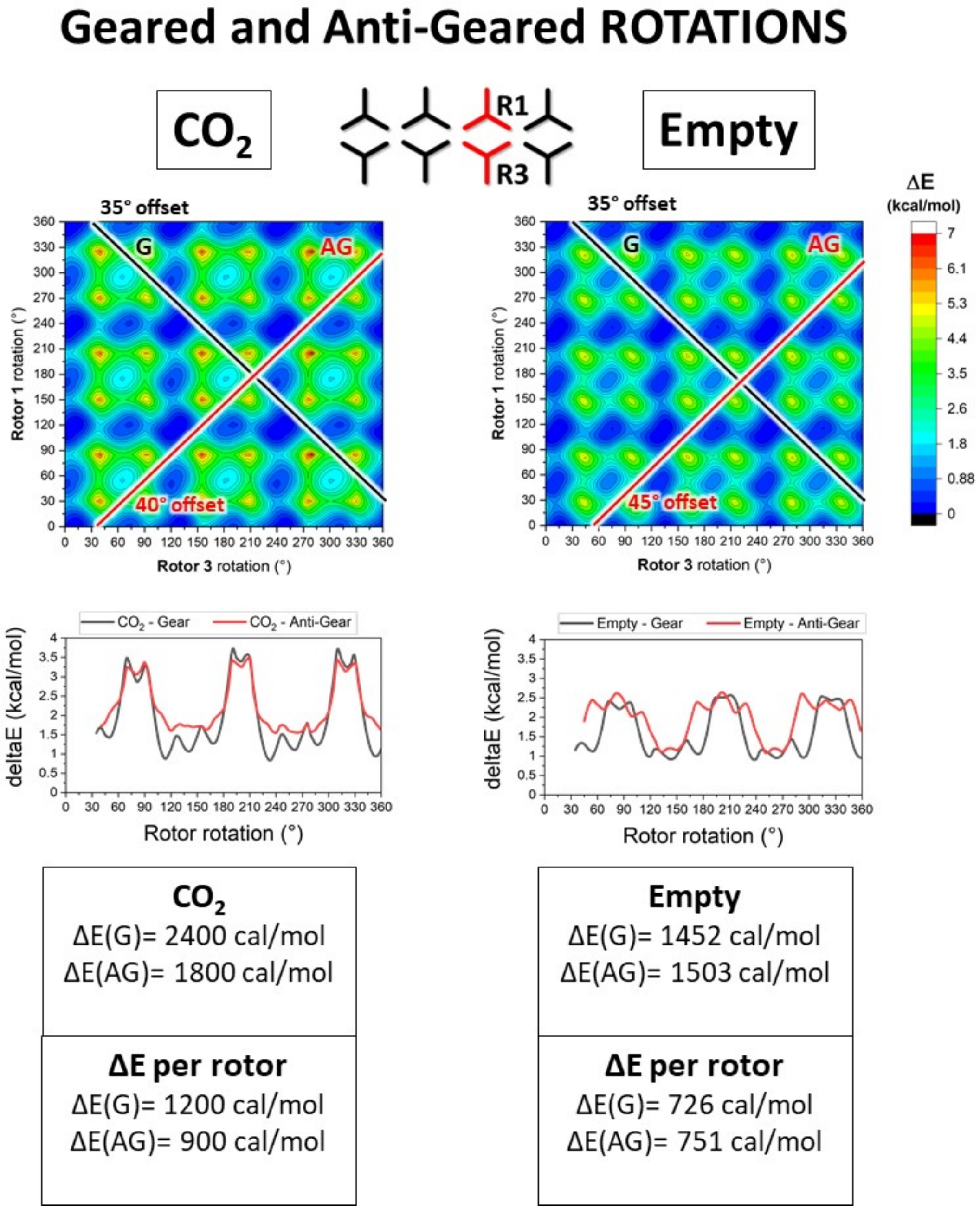

Figure S78. The 2D DFT scans results for **R1-R3** as calculated by molecular mechanics (2 x 2 supercell) for the empty (**FTR-P1d**, α-phase) and CO_2 loaded (**FTR-P1d·4CO$_2$**, α'-phase) structures. Top: graphical illustration of the model. Middle: The 2D contour maps with the rotation of rotors pairs **R1** and **R3** on the Y- and X-axes with the colours indicating the ΔE. The colour scale ranges from 0 kcal/mol (blue) to 7 kcal/mol (red). The lines indicate the most optimal Gearing (G) and Anti-gearing (AG) pathways on the energy surfaces, with the relative rotor rotational offset indicated for each. Bottom: The ΔE plot for the most optimal gearing and anti-gearing rotations. The DFT and MM ΔE (energy barrier) for G and AG are shown per rotor.

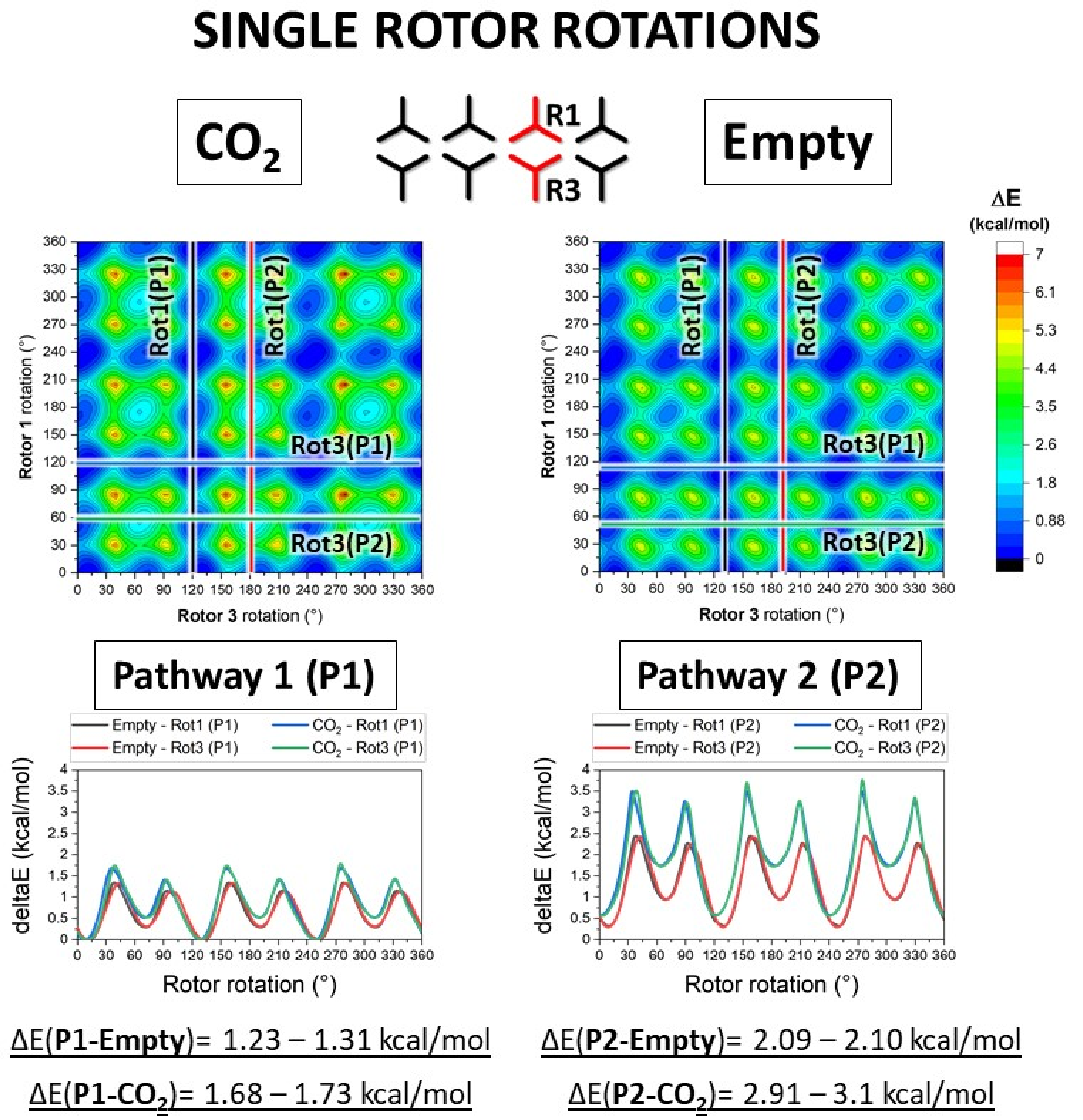

Figure S79. The 2D DFT scans results for **R1-R3** as calculated by molecular mechanics (2 x 2 supercell) for the empty (**FTR-P1d**, α-phase) and CO_2 loaded (**FTR-P1d·4CO₂** → **FTR-P1d·4CO_2**, α'-phase) structures. Top: graphical illustration of the model. Middle: The 2D contour maps with the rotation of rotors pairs **R1** and **R3** on the Y- and X-axes with the colours indicating the ΔE. The colour scale ranges from 0 kcal/mol (blue) to 7 kcal/mol (red). The lines indicate the most optimal single rotor rotation pathways on the energy surfaces. Bottom: The ΔE plot for the most optimal single rotor rotations. The **FTR-P1d·4CO_2** and **FTR-P1d** ΔE (energy barrier) are shown per rotor. **<u>Pathway 1 (P1):</u>** The rotation rotor **R1** with **R3** in the Global minimum position (0 - 15°) and the rotation rotor **R3** with **R1** in the Global minimum position (0 - 15°). **<u>Pathway 2 (P2):</u>** The rotation rotor **R1** with **R3** in the Local minimum position (60 - 75°) and the rotation rotor **R3** with **R1** in the Local minimum position (60 - 75°).

Table S21. Summary of the energy barriers for the different rotational pathways for rotors pairs **R1-R2** and **R1-R3**.

	**DFT**	**MM-Empty**	**MM-CO_2**
Geared **R1-R2**	72.5 cal/mol	285 cal/mol	632 cal/mol
Anti-Geared **R1-R2**	310 cal/mol	295 cal/mol	821 cal/mol
Geared **R1-R3**	752 cal/mol	726 cal/mol	1200 cal/mol
Anti-Geared **R1-R3**	925 cal/mol	751 cal/mol	900 cal/mol
**Single rotor rotations**			
**P1** (global min) **R1-R2**	0.9 – 1.0 kcal/mol	1.32 – 1.37 kcal/mol	1.64 – 2.07 kcal/mol
**P2** (local min) **R1-R2**	0.9 – 1.0 kcal/mol	1.29 – 1.32 kcal/mol	1.58 – 1.94 kcal/mol
**P1** (global min) **R1-R3**	0.96 – 1.0 kcal/mol	1.23 – 1.31 kcal/mol	1.68 – 1.73 kcal/mol
**P2** (local min) **R1-R3**	1.23 – 1.31 kcal/mol	2.09 – 2.10 kcal/mol	2.91 – 3.1 kcal/mol

RIETVELD REFINEMENT OF PXRD PROFILES

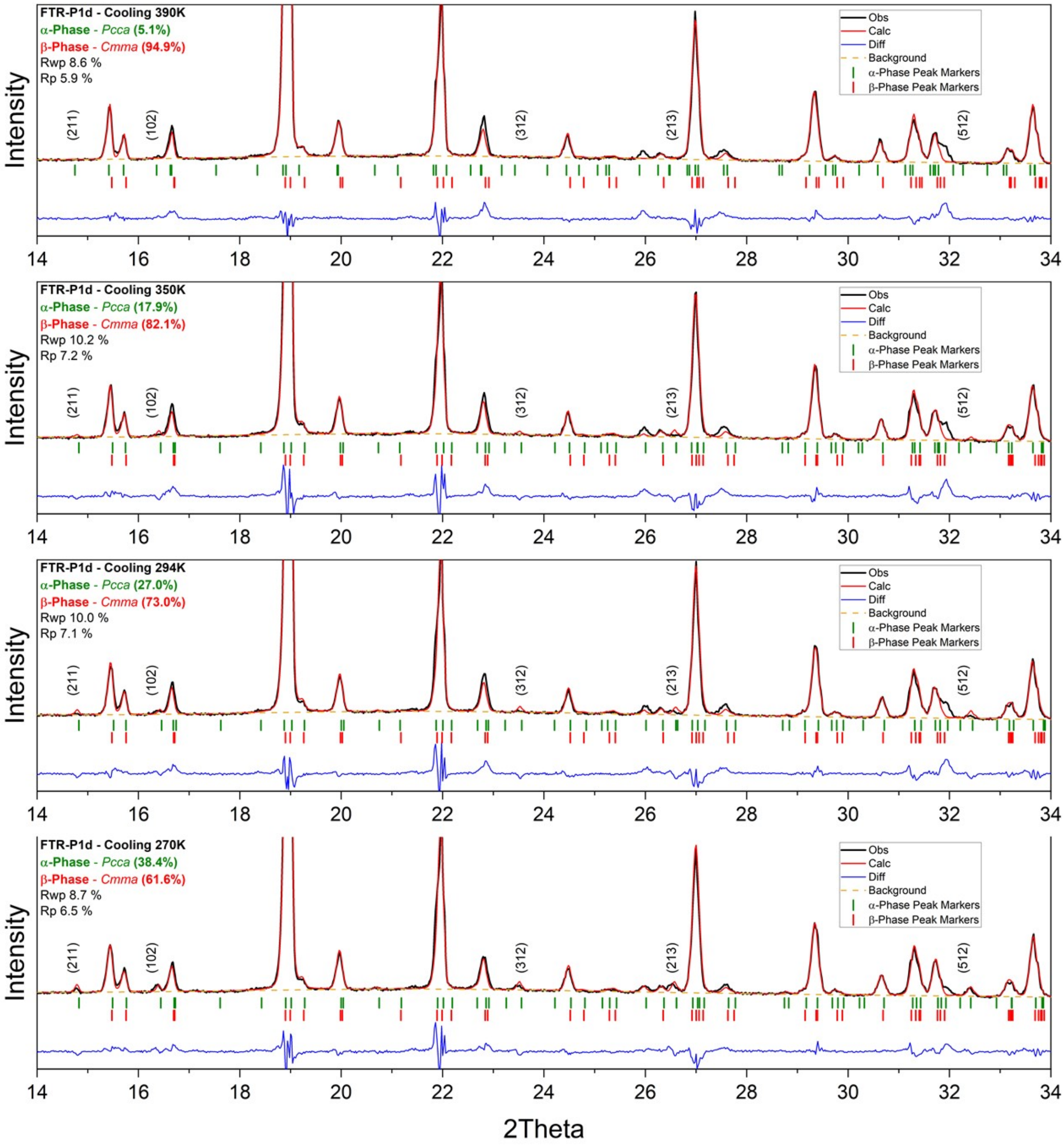

Figure S80. The 2-phase Rietveld refinement of FTR-P1d for the PXRD traces collected under vacuum and cooling from 390 K to 270 K. The miller indices indicated are for peaks only present in the α-phase.

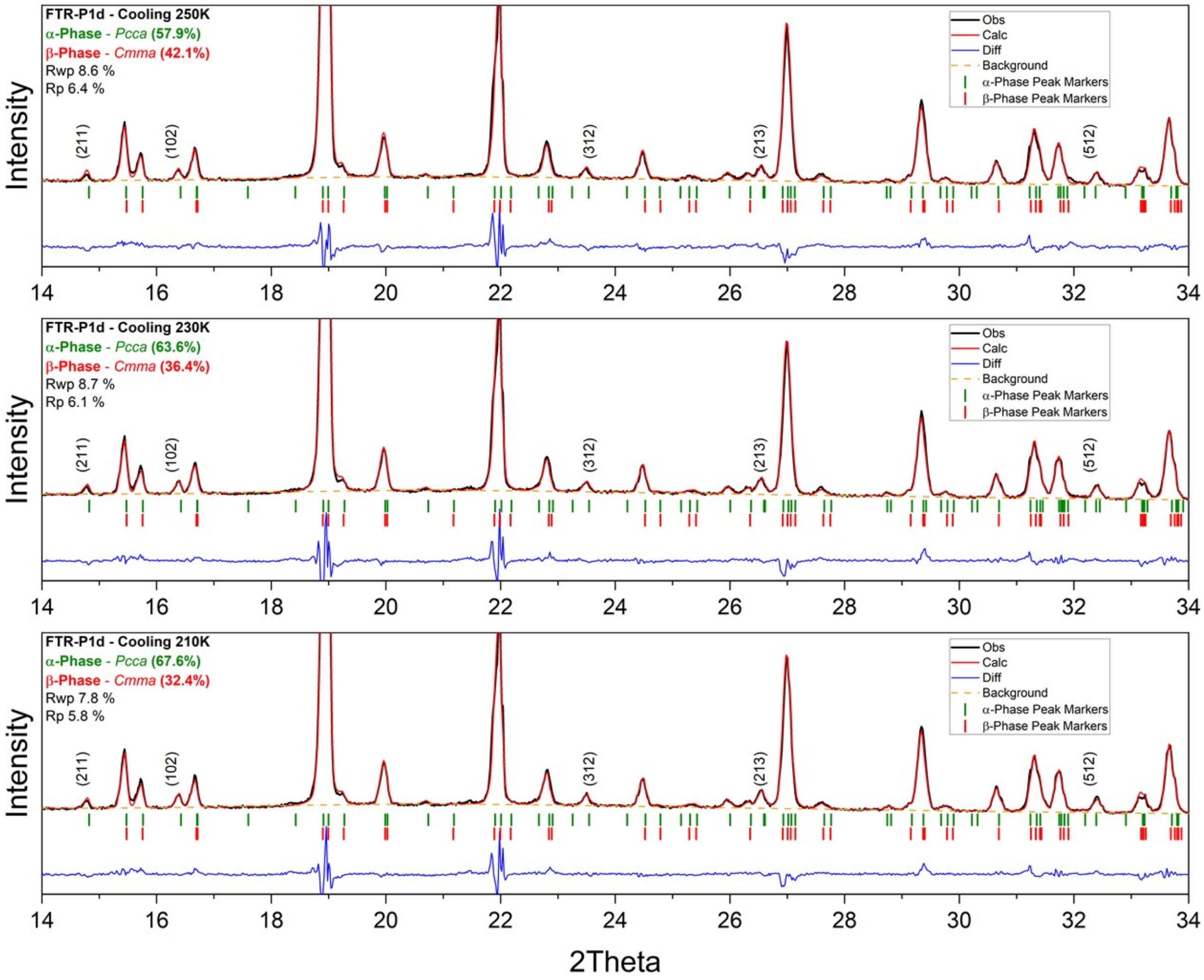

Figure S81. The 2-phase Rietveld refinement of FTR-P1d for the PXRD traces collected under vacuum and cooling from 250 K to 210 K. The miller indices indicated are for peaks only present in the α-phase.

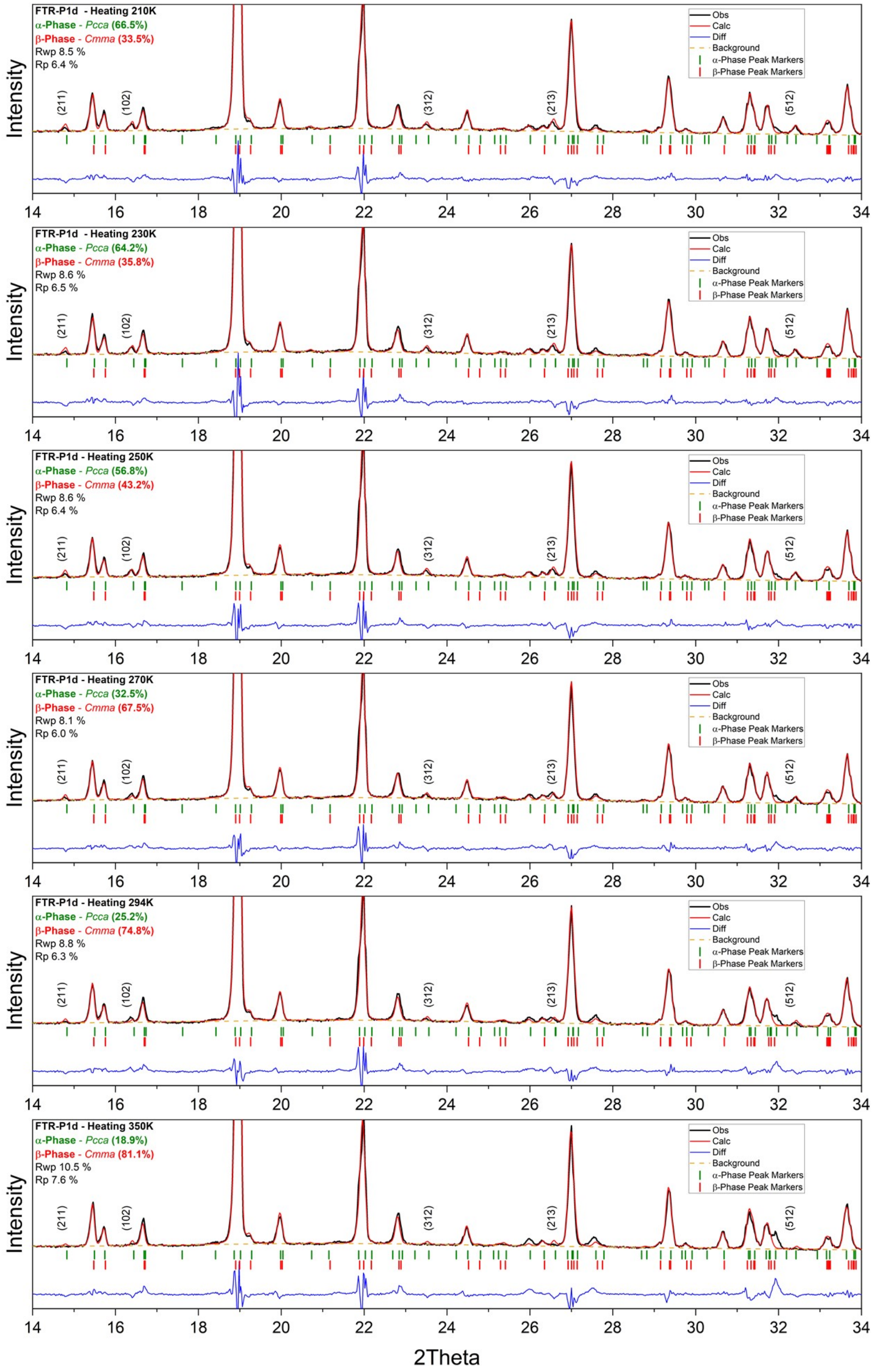

Figure S82. The 2-phase Rietveld refinement of FTR-P1d for the PXRD traces collected under vacuum and heating from 210 K to 350 K. The miller indices indicated are for peaks only present in the α-phase.

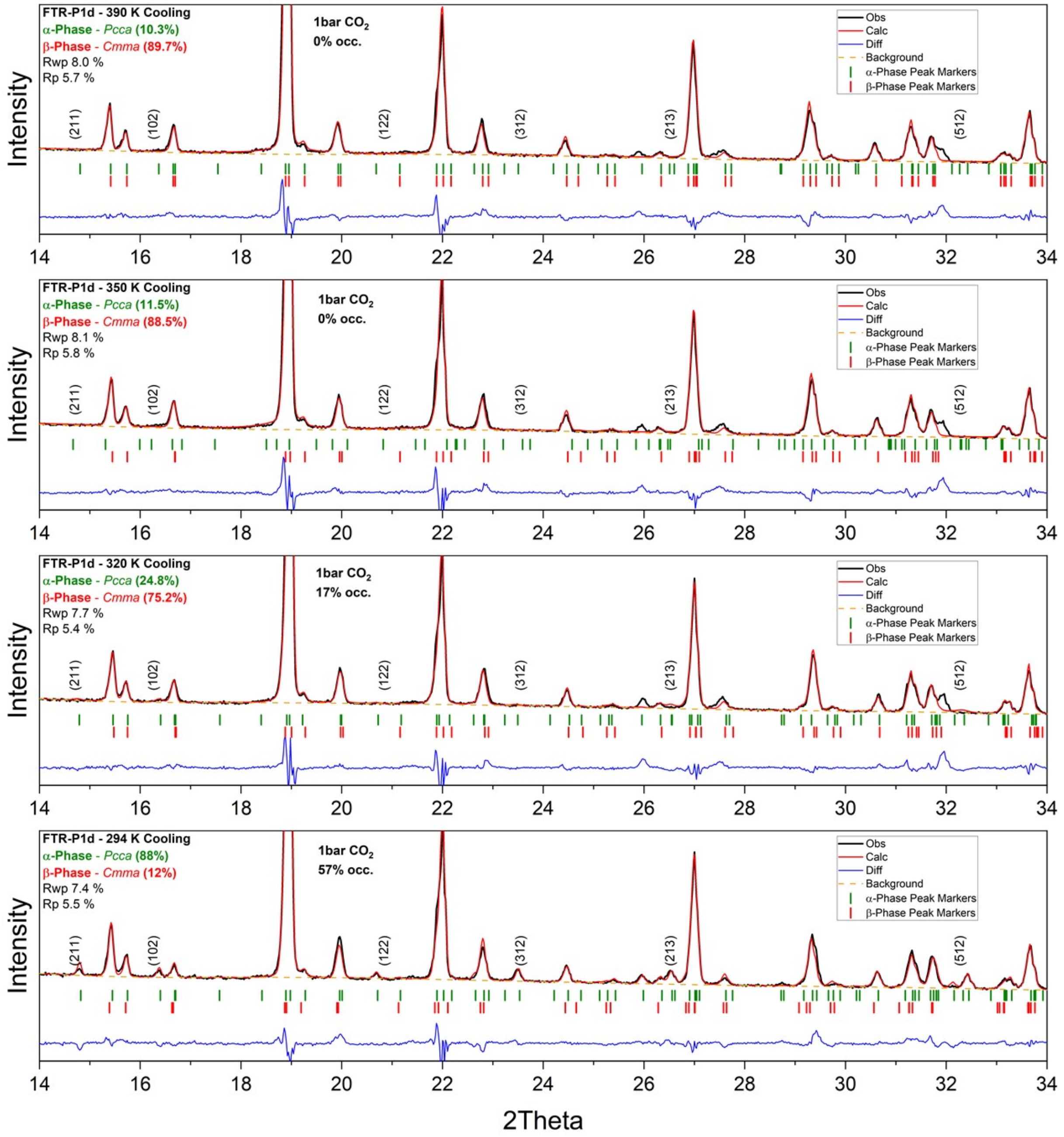

Figure S83. The 2-phase Rietveld refinement of FTR-P1d loaded with 1 bar CO_2 for the PXRD traces collected under vacuum and heating from 390 K to 294 K. The Miller indices indicated are for peaks only present in the α-phase.

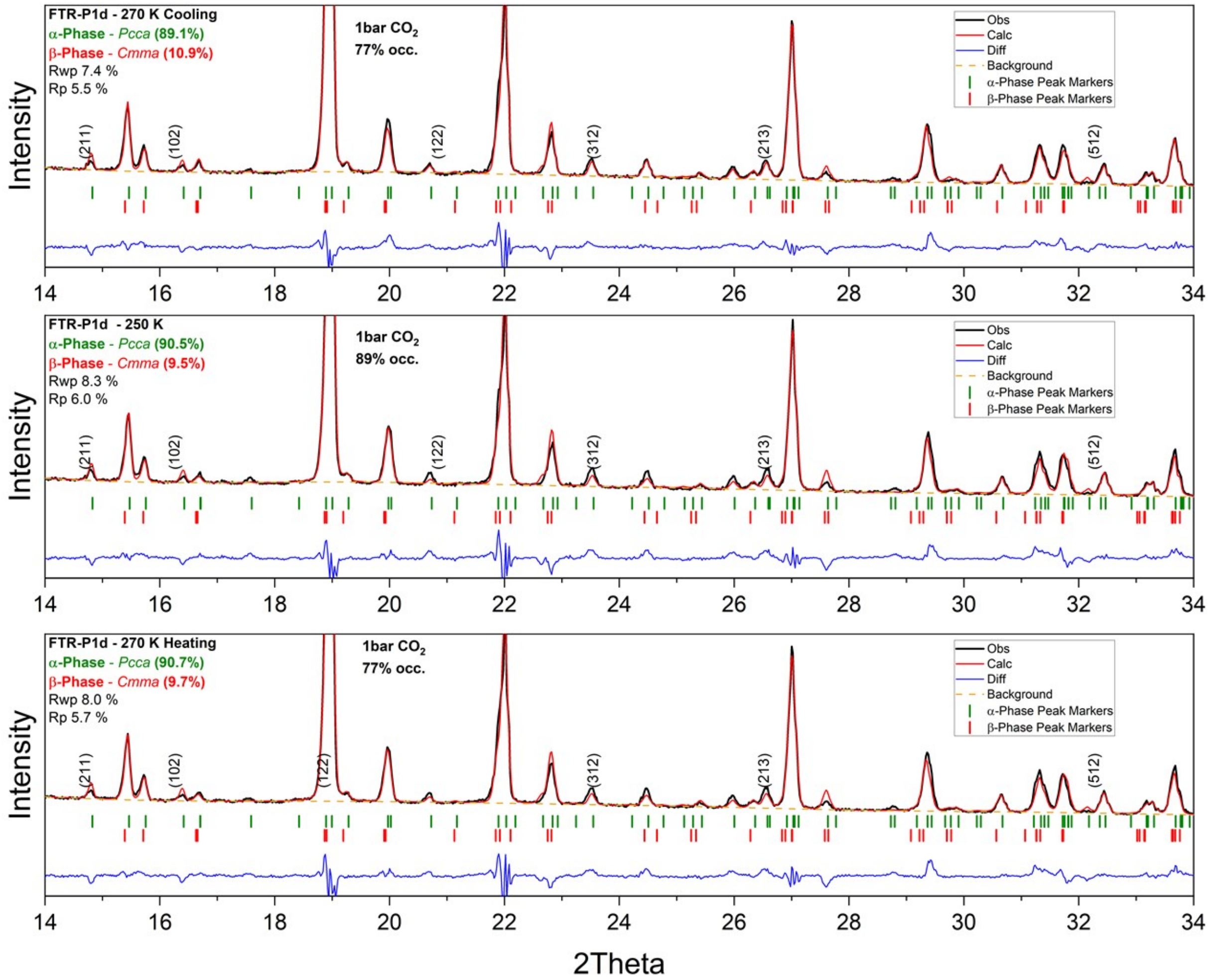

Figure S84. The 2-phase Rietveld refinement of FTR-P1d loaded with 1 bar CO_2 for the PXRD traces collected under vacuum and heating from 270 K - 250 K - 270 K. The Miller indices indicated are for peaks only present in the α-phase.

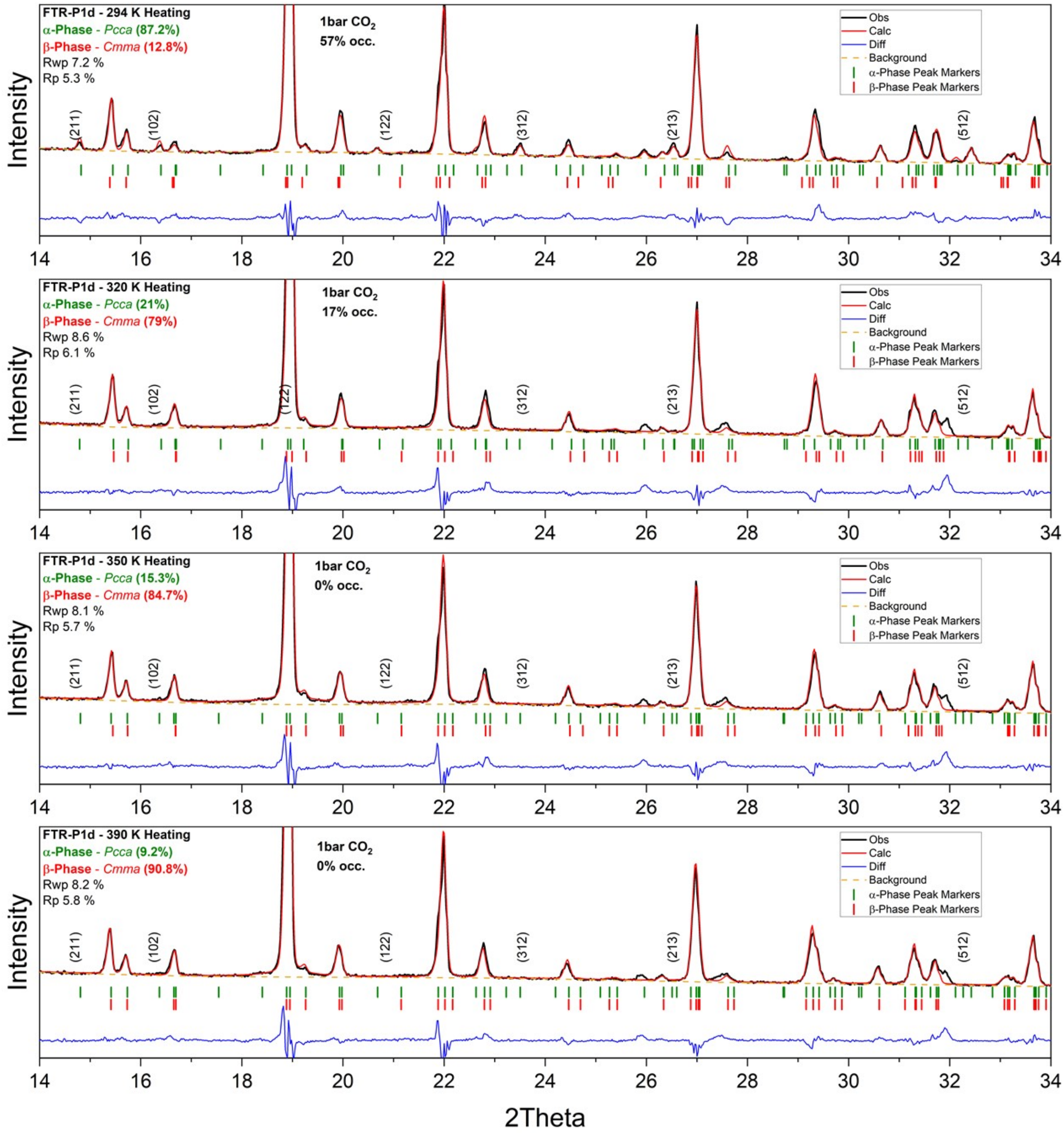

Figure S85. The 2-phase Rietveld refinement of FTR-P1d loaded with 1 bar CO_2 for the PXRD traces collected under vacuum and heating from 294 K to 390 K. The Miller indices indicated are for peaks only present in the α-phase.

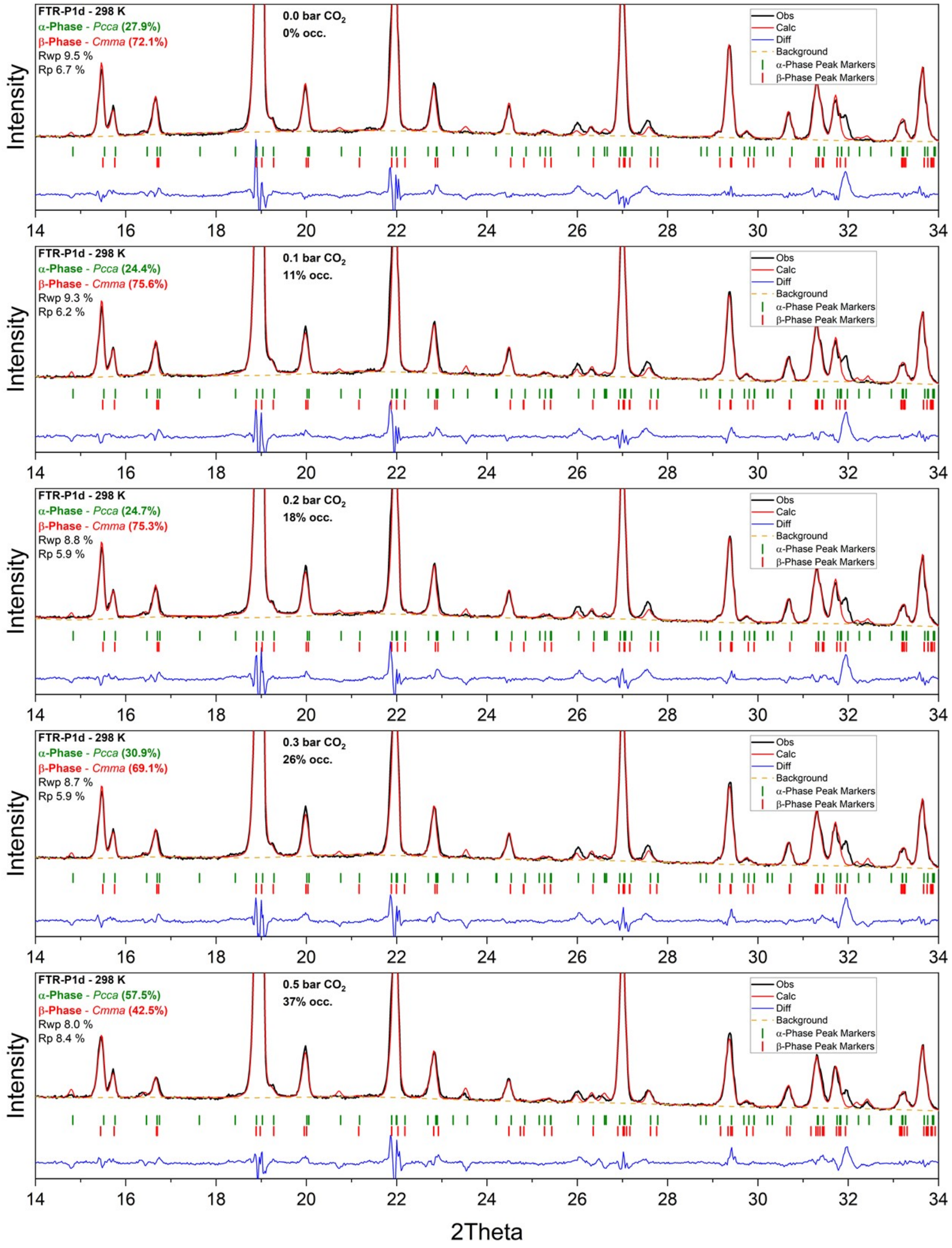

Figure S86. The 2-phase Rietveld refinement of FTR-P1d for the PXRD traces collected at 298 K under variable pressure from 0 bar – 0.5 bar.

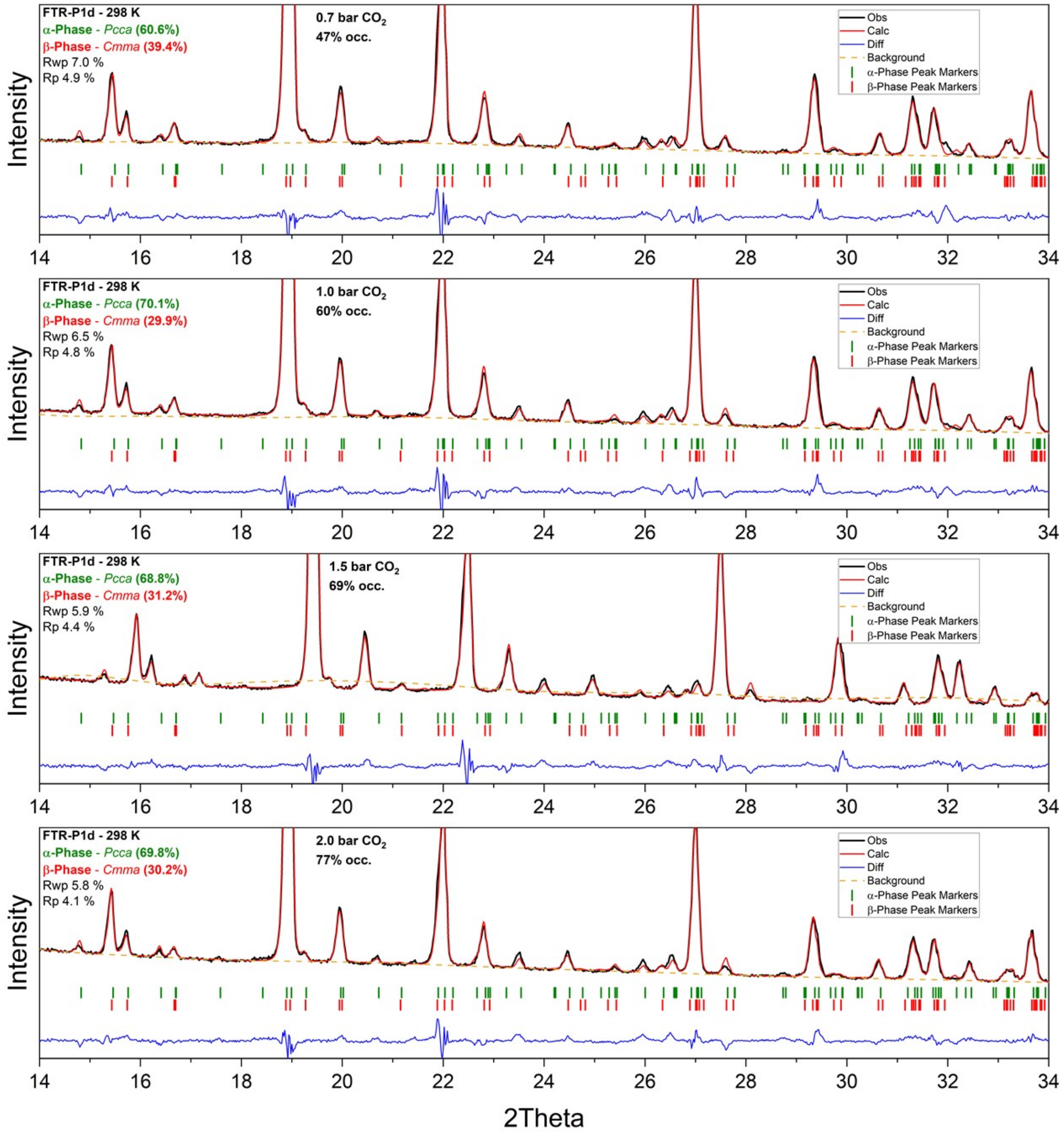

Figure S87. The 2-phase Rietveld refinement of FTR-P1d for the PXRD traces collected at 298 K under variable pressure from 0.7 bar – 2 bar.

References

1. Bruker (2012). *SMART*. Bruker AXS Inc., Madison, Wisconsin, USA.
2. L. Krause, R. Herbst-Irmer, G. M. Sheldrick, D. Stalke (2015). J. Appl. Cryst. 48, 3-10
3. G. M. Sheldrick (2015). SHELXT – Integrated space-group and crystal-structure determination. Acta Crystallographica Section A, 71, 3-8.
4. G. M. Sheldrick (2015). Crystal structure refinement with SHELXL. Acta Crystallographica Section C, 71, 3-8.

5. O. V. Dolomanov, L. J. Bourhis, R. J. Gildea, J. A. K. Howard, H. Puschmann, OLEX2: A complete structure solution, refinement and analysis program (2009). J. Appl. Cryst., 42, 339-341
6. (a) A. A. Coelho, Indexing of powder diffraction patterns by iterative use of singular value decomposition (2003). J. Appl. Cryst. 36, 86–95. (b) A. A.Coelho, A. Kern, Discussion of the indexing algorithms within TOPAS (2005). CPD Newsletter 32, 43-45.
7. Materials Studio: Materials Studio Modeling Environment v7.0.0; Accelrys Software Inc.: San Diego, (2015).
8. G. Metz, X. Wu, S. O. Smith (1994). J. Magn. Reson. A 110, 219-227.
9. a) E. Vinogradov, P. K. Madhu, S. Vega (1999). Chem. Phys. Lett. 314, 443-450; b) M. Leskes, P. K. Madhu, S. Vega (2007). Chem. Phys. Lett. 447, 370-374.
10. A. E.Bennet, C. M. Rienstra, M. Auger, K. V. Lakshmi, R. G. Griffin (1995). J. Chem. Phys. 103, 6951-6958.
11. R. L.Vold, G. L. Hoatson (2009). J. Magn. Reson. 198, 57– 72.
12. V. Macho, L. Brombacher, H.W. Spiess (2001). Appl. Magn. Reason. 20, 405-432.
13. D. M. Rice, R. J. Wittebort, R. G. Grifffin, E. Meirovitch, E. R. Stimson, Y. C. Meinwald, J. H. Freed, H. A. Scheraga, (1981) J. Am Chem. Soc. 103, 7707-7710.
14. R. Kubo, R., K. Tomita (1954). J. Phys. Soc. Jpn. 9, 888-919.
15. Gaussian 16, Revision B.01, M. J. Frisch, G. W. Trucks, H. B. Schlegel, G. E. Scuseria, M. A. Robb, J. R. Cheeseman, G. Scalmani, V. Barone, G. A. Petersson, H. Nakatsuji, X. Li, M. Caricato, A. V. Marenich, J. Bloino, B. G. Janesko, R. Gomperts, B. Mennucci, H. P. Hratchian, J. V. Ortiz, A. F. Izmaylov, J. L. Sonnenberg, D. Williams-Young, F. Ding, F. Lipparini, F. Egidi, J. Goings, B. Peng, A. Petrone, T. Henderson, D. Ranasinghe, V. G. Zakrzewski, J. Gao, N. Rega, G. Zheng, W. Liang, M. Hada, M. Ehara, K. Toyota, R. Fukuda, J. Hasegawa, M. Ishida, T. Nakajima, Y. Honda, O. Kitao, H. Nakai, T. Vreven, K. Throssell, J. A. Montgomery, Jr., J. E. Peralta, F. Ogliaro, M. J. Bearpark, J. J. Heyd, E. N. Brothers, K. N. Kudin, V. N. Staroverov, T. A. Keith, R. Kobayashi, J. Normand, K. Raghavachari, A. P. Rendell, J. C. Burant, S. S. Iyengar, J. Tomasi, M. Cossi, J. M. Millam, M. Klene, C. Adamo, R. Cammi, J. W. Ochterski, R. L. Martin, K. Morokuma, O. Farkas, J. B. Foresman, and D. J. Fox, Gaussian, Inc., Wallingford CT, 2016.
16. N. Mardirossian, M. Head-Gordon, Thirty years of density functional theory in computational chemistry: an overview and extensive assessment of 200 density functionals (2017). Molecular Physics 115, 2315-2372.
17. a) W. K. Feldmann, K.-A. White, C. X. Bezuidenhout, V. J. Smith, C. Esterhuysen, L. J. Barbour (2020). ChemSusChem. 13, 102-105. b) J. Perego, C. X. Bezuidenhout, A. Pedrini, S. Bracco, M. Negroni, A. Comotti, P. Sozzani (2020). J. Mater. Chem. A 8, 11406-11413.
